# Enhancement of sleep slow wave activity using transcranial electrical stimulation with temporal interference: an interim analysis of the STRENGTHEN study

**DOI:** 10.1038/s43856-026-01744-x

**Published:** 2026-07-01

**Authors:** Erin L. Schaeffer, Ido Haber, Zhiwei Fan, Simone Bruno, Beril Mat, Tariq Alauddin, Giulietta Vigueras, Lindsey Neumann, Richard Smith, Florian Missey, Adam Williamson, Peter Achermann, Stefan Beerli, Myles Capstick, Esra Neufeld, Niels Kuster, Robin I. Goldman, Richard J. Davidson, William Marshall, Larissa Albantakis, Stephanie G. Jones, Chiara Cirelli, Melanie Boly, Giulio Tononi

**Affiliations:** 1 Department of Psychiatry, University of Wisconsin-Madison, Madison, WI USA; 2 Medical Scientist Training Program, University of Wisconsin-Madison, Madison, WI USA; 3 Department of Biomedical Engineering, University of Wisconsin-Madison, Madison, WI USA; 4 International Institute for Integrative Sleep Medicine (WPI-IIIS), Tsukuba Institute for Advanced Research (TIAR), University of Tsukuba, Tsukuba, Japan; 5 International Clinical Research Center, St. Anne’s University Hospital Brno, Brno, Czechia; 6 Institute de Neurosciences des Systèmes (INS), INSERM, Aix-Marseille Université, Marseille, France; 7 Institute of Pharmacology and Toxicology, University of Zurich, Zurich, Switzerland; 8 Foundation for Research on Information Technologies in Society (IT’IS), Zurich, Switzerland; 9 Department of Information Technology and Electrical Engineering, Swiss Federal Institute of Technology (ETH) Zurich, Zurich, Switzerland; 10 Center for Healthy Minds, University of Wisconsin-Madison, Madison, WI USA; 11 Department of Psychology, University of Wisconsin-Madison, Madison, WI USA; 12 Department of Mathematics and Statistics, Brock University, St. Catharines, ON Canada; 13 Department of Neurology, University of Wisconsin-Madison, Madison, WI USA

**Keywords:** Circadian rhythms and sleep, Non-REM sleep

## Abstract

**Background:**

Slow waves are oscillations that reflect rhythmic alternation of neuronal activity and mediate key restorative functions of non-rapid eye movement (NREM) sleep. Left ventromedial prefrontal cortex regions are a “hot spot” for slow wave generation. The enhancement of slow wave activity (SWA, 0.5-4 Hz) has been shown to be beneficial, as in improving memory performance. To overcome limitations of current techniques, we assessed the ability of a non-invasive neuromodulatory tool, Transcranial Electrical Stimulation with Temporal Interference (TES-TI), to enhance SWA during NREM sleep overnight in healthy humans.

**Methods:**

The current study is an interim analysis focused on the effects of TES-TI during NREM sleep in a laboratory setting. Two high frequency carriers with 1 Hz difference (TES^15kHz^-TI^1Hz^) produced amplitude-modulated temporal interference at 1Hz. Data were collected at the University of Wisconsin-Madison as part of the STRENGTHEN study (ClinicalTrials.gov, NCT06267521, 02/12/2024, single-blind, non-random allocation). Eligible participants were healthy adults (ages 18–50) assigned in parallel to one of four groups: (Group 1) TES^15kHz^ (0 Hz difference frequency) 2 nights per week and meditation; (Group 2) TES^15kHz^-TI^1Hz^ (1 Hz difference frequency) 2 nights per week and sham meditation; (Group 3) TES^15kHz^-TI^1Hz^ 1 night per week and meditation; (Group 4) TES^15kHz^-TI^1Hz^ 2 nights per week and meditation. Stimulation targeting left ventromedial prefrontal cortex occurred during NREM sleep over repeated nights ( ~ 10 stimulation periods, 3-min each, first half of night, 1-2 nights/week, 4-week protocol). Twenty-one participants (Groups 2, 3, 4) received TES^15kHz^-TI^1Hz^ and seven participants received TES^15kHz^ (Group 1). SWA was measured using simultaneous high-density electroencephalography with polysomnography.

**Results:**

We show that SWA is increased with TES^15kHz^-TI^1Hz^ (*N* = 21 total; *N* = 5 Group 2, *N* = 10 Group 3, *N* = 6 Group 4), and the effect outlasts the stimulation period; higher frequencies (sigma and beta) decrease. During stimulation, SWA is greater in TES^15kHz^-TI^1Hz^ than pure TES^15kHz^ (*N* = 7 Group 1). The incremental effects of TES^15kHz^-TI^1Hz^ on SWA between first and last intervention night are positively associated with subjective ratings of restorative sleep.

**Conclusions:**

To our knowledge, this is the first study demonstrating that TES-TI enhances SWA and potentially its restorative function.

## Plain Language Summary

Slow waves are signature patterns of electrical activity indicative of deep sleep. They play an important role in the ability of sleep to be and feel restorative. Boosting slow wave activity has been shown to be beneficial for memory and cognition but the current techniques for doing so have limitations. Here, we use a non-invasive technique—Transcranial Electrical Stimulation with Temporal Interference (TES-TI)—that overcomes these limitations and allows for sensation-free modulation of deep brain regions that can be performed with simultaneous electroencephalography (EEG) to record brain activity during sleep. The main findings support the ability of TES-TI to enhance slow wave activity during non-rapid eye movement sleep in healthy humans. Future studies that build upon this work by investigating the TES-TI mechanism of action and optimizing parameters will serve as important steps toward the utilization of TES-TI for the improvement of sleep.

## Abstract (web summary)

Schaeffer et al. assess the use of Transcranial Electrical Stimulation with Temporal Interference (TES-TI) and high-density electroencephalography during overnight sleep in healthy humans. Findings show that TES-TI enhances slow wave activity during and after stimulation, where the former is associated with improved restorative sleep ratings.

## Introduction

During non-rapid eye movement (NREM) sleep, which comprises 75–80% of total time spent in sleep, individual slow waves are detected on electroencephalography (EEG) when many cortical neurons switch near-synchronously between ON periods of firing and OFF periods of silence^1^. Sleep slow waves are homeostatically regulated. Thus, slow wave activity (SWA) (EEG power in the 0.5–4 Hz range), a combined measure of the number and amplitude of slow waves, is high in early sleep, even higher after sleep deprivation, and declines in the course of sleep when sleep need is discharged^2–4^. SWA is maximal during the deepest stages of sleep. Slow waves are believed to mediate key restorative functions attributed to NREM sleep, including synaptic renormalization and memory consolidation^5–11^. Slow waves, in conjunction with spindles and sharp-wave ripples, may coordinate the reorganization of memories within the cortex^9–11^. Disrupted SWA is associated with worsened cognitive functioning, including poorer performance on visuomotor and perceptual learning tasks^12–14^. Conversely, the enhancement of SWA has been shown to improve cognitive performance in several domains, such as verbal fluency, working memory, and declarative memory^15–19^.

Two interventions commonly used to enhance SWA are acoustic stimulation and transcranial electrical stimulation (TES)^13,15–20^. However, both techniques have limitations. Acoustic stimulation strong enough to elicit SWA can be close to the threshold for awakening the participant^13^. Conventional TES, which typically employs frequencies below 1 kHz, results in electrical artifacts during stimulation that prevent adequate evaluation of simultaneous EEG recordings, particularly at the stimulation frequency^18^. In addition, conventional TES stimulates broadly, affecting superficial regions with diminishing efficacy over deeper brain regions when using parameters that do not produce scalp discomfort^21^.

An innovative neuromodulatory tool potentially able to overcome these limitations is transcranial electrical stimulation with temporal interference (TES-TI), also called temporal interference stimulation (TIS)^22^ or interferential current stimulation^23–26^. It uses two or more high-frequency alternating current carriers (e.g., 15 kHz and 15 kHz + 1 Hz) to produce “temporal interference,” that is, amplitude modulation at the difference frequency (e.g., 1 Hz) capable of modulating neural activity at a targeted location in an individual’s brain^22^. This approach achieves both depth of penetration and focality^22^. Moreover, TES-TI is steerable, in a way not easily attainable with other non-invasive neuromodulation techniques, by adjusting the current ratio of the two channel pairs^22^. Importantly, high-frequency carriers allow for effective stimulation intensities without the participant perceiving any peripheral stimulation that could cause awakening from sleep^27,28^. While the exact mechanism by which TES-TI modulates neural activity is an area of active investigation^22,26,29,30^, several studies have now implemented TES-TI with high frequency carriers (up to 20 kHz) in a safe and effective manner in humans^31–35^. For example, in healthy populations, reported effects include changes in connectivity^33,34,36^ and/or behavior on various tasks, including motor learning, episodic memory, and spatial navigation^34–38^. Currently, less is known in clinical populations; however, preliminary evidence suggests potential benefits for individuals with Parkinson’s disease or epilepsy^34,39^. To our knowledge, no study has reported on the use of TES-TI to promote sleep overnight, and more specifically, to enhance SWA.

The current study is an interim analysis of data from the STRENGTHEN study that focuses on the effects of TES-TI during NREM sleep and uses data from simultaneously recorded high-density EEG (hd-EEG) and TES-TI during overnight sleep in a laboratory setting in healthy humans. TES^15 kHz^-TI^1 Hz^ (15 kHz carrier, 1 Hz difference) targeted left ventromedial prefrontal cortical regions, given evidence from source modeling^40^ and invasive recordings^41^ suggesting that these areas are a “hot spot” for slow wave generation. With hardware filters, simultaneous hd-EEG recording was obtained while TES^15 kHz^-TI^1 Hz^ was delivered during periods of NREM sleep in the first half of the night. The main results show that SWA increases during and after TES^15 kHz^-TI^1 Hz^. Low frequencies (SWA, theta) increase and high frequencies (sigma and beta) decrease with TES^15 kHz^-TI^1 Hz^. During stimulation, SWA is greater in TES^15 kHz^-TI^1 Hz^ as compared to TES^15 kHz^ (15 kHz carrier only). Lastly, the increases in SWA with TES^15 kHz^-TI^1 Hz^ between first and last intervention nights are positively associated with improved ratings of restorative sleep quality. Altogether, these results provide evidence in support of the ability of TES^15 kHz^-TI^1 Hz^ to enhance sleep SWA and potentially its restorative functions.

## Methods

### Study design

**Fig. 1:**
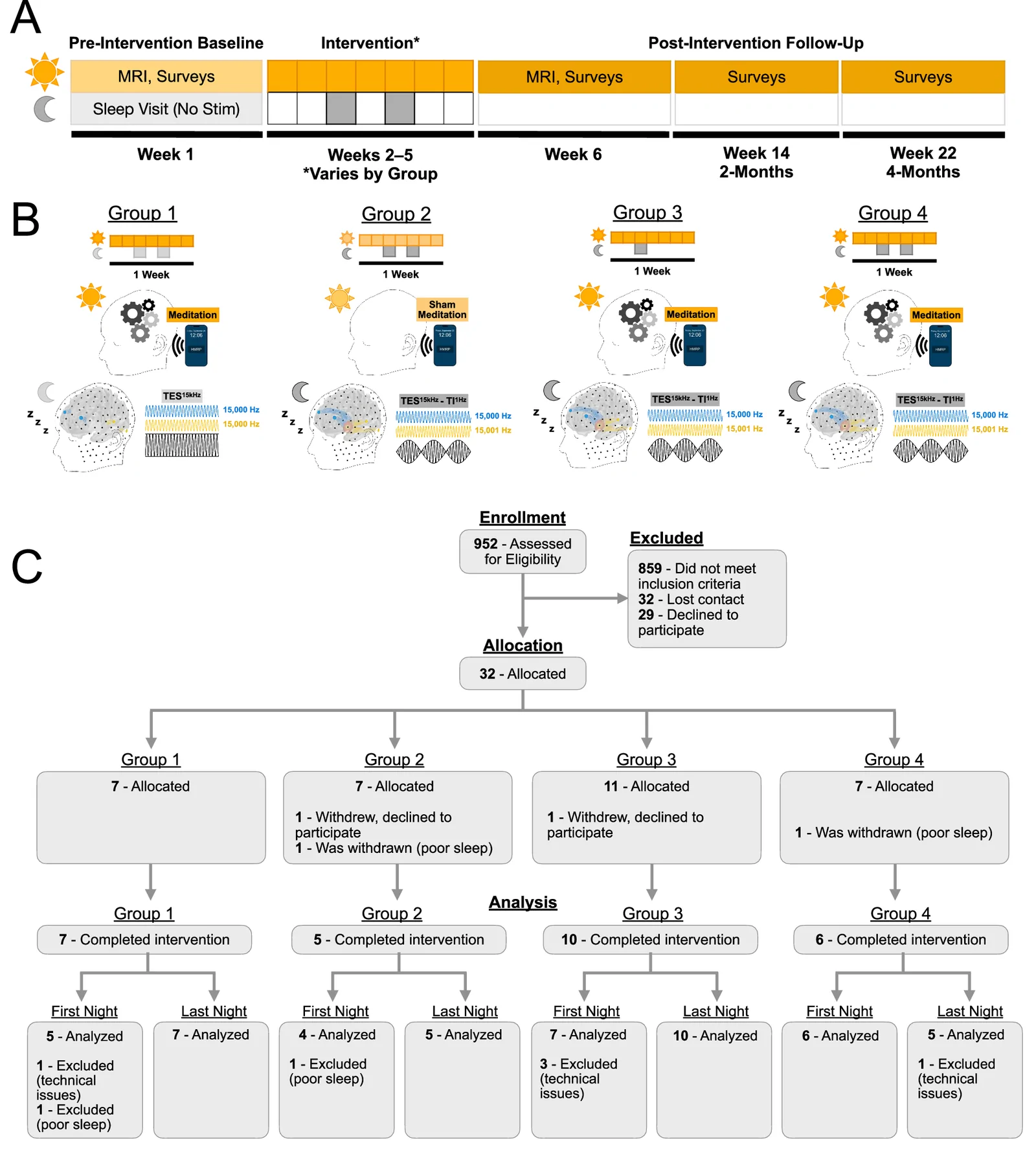
STRENGTHEN study overview. **A** Timeline of events for study participants, divided into daytime (top, orange) and nighttime (bottom, grey) events. Pre-intervention baseline events consisted of surveys, a magnetic resonance imaging (MRI) session, and overnight sleep visit (no stimulation performed). The intervention period lasted 4-weeks and consisted of interventions that varied according to group assignment. All groups performed meditation (or sham meditation) training daily; stimulation interventions occurred once or twice per week at night. Post-intervention follow-up events consisted of an MRI and surveys, which were repeated at 2 months and 4 months. **B** Interventions varied according to assigned group (non-random allocation, parallel assignment). Group 1 performed meditation training daily and received transcranial electrical stimulation (TES^15 kHz^) twice per week at night. Group 2 performed sham meditation daily and received transcranial electrical stimulation with temporal interference (TES^15 kHz^-TI^1 Hz^) stimulation twice per week at night. Group 3 performed meditation training daily and received TES^15 kHz^-TI^1 Hz^ stimulation once per week at night. Group 4 performed meditation training daily and received TES^15 kHz^-TI^1 Hz^ stimulation twice per week at night. TES^15 kHz^-TI^1 Hz^ used 15 kHz carrier frequency and 1 Hz difference frequency; TES^15 kHz^ used only a 15 kHz carrier frequency. More information about TES-TI can be found in Fig. 2. **C** Participant flow diagram for the data collection period from February 19, 2024, to December 31, 2024. Thirty-two participants met the inclusion criteria and were allocated to intervention groups. Two participants withdrew themselves from the study after their baseline assessments. Two participants were withdrawn by study personnel after evaluation of their baseline sleep visits showed poor sleep with concern for possible obstructive sleep apnea. All remaining allocated participants completed the intervention and follow-up activities. For the analysis, the number of participants in each group, for each night, are listed. Further information can be found in the “Datasets included” section of the “Methods” and Supplementary Fig. 1.

Data used in this study were acquired as part of the STRENGTHEN clinical trial (ClinicalTrials.gov, NCT06267521, registration date: 02/12/2024), a single-blind, longitudinal study aimed at enhancing cognitive flexibility and emotion regulation. The study design includes TES-TI delivered during sleep in the lab as well as brief, daily meditation training (Fig. 1A). Healthy participants (ages 18–50) were assigned in parallel to 1 of 4 groups: (Group 1) TES^15 kHz^ (0 Hz difference frequency) 2 nights per week and meditation; (Group 2) TES^15 kHz^-TI^1 Hz^ (1 Hz difference frequency) 2 nights per week and sham meditation; (Group 3) TES^15 kHz^-TI^1 Hz^ 1 night per week and meditation; (Group 4) TES^15 kHz^-TI^1 Hz^ 2 nights per week and meditation (Fig. 1B). For all participants, interventions during sleep in the lab occurred over four consecutive weeks. Participants were assigned to their treatment group following pre-intervention baseline assessment, with efforts made during non-random allocation to ensure group equivalence on key demographic variables, particularly age and sex. This matching approach mitigated potential confounds related to sex and age-based differences in sleep. Participants were blinded to their experimental group, i.e., they did not know whether they were receiving TES^15 kHz^ (Group 1) or TES^15 kHz^-TI^1 Hz^ (Groups 2, 3, and 4). Participants were reimbursed for their study participation. Neither participants nor the public were involved in the design, conduct, or reporting of this trial. All aspects of the study were approved by the University of Wisconsin-Madison Institutional Review Board.

The current study reflects a mechanistic sub-study within the broader STRENGTHEN trial; it is an interim analysis that focuses on the effects of TES^15 kHz^-TI^1 Hz^ during NREM sleep using data collected from the first five cohorts of the STRENGTHEN trial (from February 19, 2024, to December 31, 2024). Each cohort contained approximately 5–6 participants who, for logistical reasons, went through the various phases of the study as a group. The analyses presented here were not pre-specified in the formal clinical trial registration; however, they were part of the planned exploratory aims within the Institutional Review Board-approved protocol and were designed prior to data collection from the analyzed cohorts. Primary and secondary endpoints of the trial related to cognitive flexibility and emotion regulation will be the focus of later work; as such, data from post-intervention follow-up periods were not included in the current work. The analyses performed here were initiated after interim inspection of the data showed large effect sizes. To maximize statistical power, the current study used data from the first five cohorts to examine the effects of TES^15 kHz^-TI^1 Hz^ on NREM sleep (*N* = 21, 1 Hz, targeting left ventromedial prefrontal cortex during NREM sleep episodes in the first half of the night) by comparing the baseline night with the first and last intervention nights regardless of the group. A comparison between TES^15 kHz^-TI^1 Hz^ and TES^15 kHz^ (i.e., directly assessing the potential effects of amplitude modulation) was also performed utilizing Group 1 participants from the first five cohorts (*N* = 7). More detailed information about statistical power and participant grouping can be found in the “Statistics and reproducibility” section. Another goal of the STRENGTHEN clinical trial, to be reported in a subsequent publication, was the assessment of TES^15 kHz^-TI^6 Hz^ (6 Hz, targeting retrosplenial cortex) during REM sleep episodes later in the night.

### Participants

In total, twenty-eight participants (30.5 ± 9.9 years, 61.0% (*N* = 17) female, *N* = 21 TES^15 kHz^-TI^1 Hz^, *N* = 7 TES^15 kHz^) from the first five cohorts of the STRENGTHEN study were included in the current study (Fig. 1C). All participants were medically healthy, with no acute illness at the time of participation and no diagnosed chronic illness, including sleep disorders, or medications that may have influenced the outcome of the study. Participants had a baseline magnetic resonance imaging (MRI) scan and a sleep night in the lab. The baseline sleep night did not include overnight stimulation. Participants were to be excluded if they had any neuroradiologist-identified structural abnormalities of the brain; no participants in the current work were excluded for this reason. Participant recruitment began with an online survey. Potential participants were then further screened by phone call to see if they met eligibility criteria. They then underwent additional screening and an informed consent discussion in person prior to potential enrollment in the study. In general, screening consisted of questions regarding demographics, availability, medical and mental health, medications, and sleep. Participants with responses meeting exclusion criteria were not included in the study. As part of screening, for example, participants were asked about current or prior diagnoses of obstructive sleep apnea (OSA); if yes, the participant was excluded. Full eligibility criteria can be found on the ClinicalTrials.gov website. As part of the pre-intervention baseline sleep visit, sleep technicians monitored for sleep quality. During the baseline sleep visit, two participants had loud snoring, breath holding and gasping accompanied by periods of wake. Given concern for OSA and overall poor sleep that would preclude experiment completion, these two participants were withdrawn after their baseline visit (Fig. 1C). All participants gave written informed consent in accordance with the University of Wisconsin-Madison Institutional Review Board.

### Datasets included

Baseline, first, and last intervention nights were utilized here; a future publication will report on all the intervention nights. In total, twenty-two first nights and twenty-seven last nights were included in subsequent analyses that evaluated the effects of stimulation. Four first nights were excluded due to technical issues, and two first nights were excluded due to poor sleep that precluded experiment completion (e.g., fewer than five out of ten intended stimulation protocols were completed). One last night was excluded due to technical issues. Analyses comparing baseline, first, and/or last intervention nights were restricted to participants with usable data across all nights. Further details about which datasets were included in the analyses can be found in Supplementary Fig. 1.

### Daytime meditation training

Meditation and sham meditation interventions were delivered via the Healthy Minds Program smartphone app, which provides a comprehensive curriculum for well-being training based upon four pillars of well-being: awareness, connection, insight, and purpose^42,43^. The program includes didactic mini-podcasts that provide the background science underlying each of the four well-being pillars, along with specific mental exercises—forms of procedural learning—that complement the declarative scientific content^42,43^. Participants assigned to the meditation intervention were guided through brief, daily meditation practice (5–30 min) and provided regular reminders of key practice goals several times each day. Each week focused on one of the four pillars of well-being. Participants assigned to the sham meditation intervention listened to didactic material only, without meditation instruction.

### Nighttime stimulation procedure

Participants spent overnights in the sleep lab at the Wisconsin Sleep Center, where they were outfitted with a 256-channel hd-EEG net for recording brain activity and additional channels for recording polysomnography (PSG) data (electrooculography adjacent to lateral canthi, submental electromyography, and electrocardiography). Channels for performing TES-TI were embedded in the hd-EEG net, placed in lieu of recording channels. While the participant was awake, short stimulation protocols (15-s with 15-s ramping up/down periods) were performed with increasing current amplitudes (up to 5 mA zero-to-peak) to determine if the participant began to feel sensation on their scalp. The current amplitude was then lowered systematically and checked again, such that the final current value of 5 mA chosen was confirmed to not elicit any sensation. Then, the participant went to sleep, and a sleep technician performed stimulation protocols based on sleep features visible on the hd-EEG and PSG recordings (data visualization parameters in accordance with staging parameters found in the “Sleep staging” section). Specifically, when NREM sleep was identified and stable (≥3 min), stimulation was delivered in 3-min protocols with 15-s ramping up/down periods. Each protocol was separated by at least 6 min and repeated approximately ten times, in most cases confined to NREM sleep episodes during the first four hours after sleep onset. Participants were monitored throughout the night from a control room, and a sleep technician was available for communication at any time.

### Sleep quality surveys

After each overnight in the sleep lab, participants completed the 9-question restorative sleep questionnaire (REST-Q) survey, which was designed by a panel of expert sleep specialists to define and measure restorative sleep^44,45^ and chosen here for its potential to link the perceived restorative effects of sleep with the SWA that may underlie these restorative effects. They rated to what extent they felt tired, sleepy, in a good mood, rested, refreshed, ready to start the day, energetic, mentally alert, and grouchy on scales of 1–5 (1 = not at all, 2 = a little bit, 3 = some, 4 = very much, 5 = completely). An overall REST-Q score was calculated in line with^44^. Overall scores from after the first and last intervention nights were included in the analyses.

### TES-TI targeting

A standardized channel montage was used for all participants (channel 1: E20–E34, channel 2: E70–E95 of the hd-EEG net) targeting left ventromedial prefrontal cortical regions. Channel placement was first approximated using the Temporal Interference Planning Tool (TIP v1.0, IT’IS Foundation, Switzerland), in which an optimal channel configuration was chosen from the 10:10 system to target the left ventromedial prefrontal cortex with maximal intensity and minimal off-target stimulation^46,47^. TIP simulations were performed using the comprehensive MIDA head model, which details the electrical properties for different tissues in a 29-year-old healthy female volunteer^48^. For further confirmation, targeting was then verified using SimNIBS software (v4.1.0) that provided tools for electric field modeling with additional details including: an individual’s MRI, and channel material, geometry, and position in the 256 hd-EEG system^49–51^. Modeling began with the segmentation of an individual participant’s T1 and T2 scans to extract the electrical properties of different tissues. Segmentation and meshing were performed using the *charm* function. The participant’s head model was then aligned to a template 256-channel hd-EEG net^51^. Next, electric fields were simulated, accounting for channel properties (see “TES-TI” section), a current value of 5 mA, and location in the 256-channel hd-EEG net extrapolated from the 10:10 system locations used in TIP simulations. Simulation of the temporal interference field (V/m) was implemented using the function *get_maxTI* that calculates the maximal modulation amplitude of the difference frequency using the equation described in refs. ^22,51^. The interference field values obtained in grey matter were then extracted and plotted over the individual’s T1 scan as well as over a standardized atlas (MNI152) for visualization using SimNIBS and Analysis of Functional NeuroImages software^52,53^.

Overall, simulations performed in SimNIBS with individualized head models agreed with those performed in TIP using the standardized MIDA head model. Based on these simulations using individualized head models, across all TES^15 kHz^-TI^1 Hz^ participants (*N* = 21), maximal intensity values in left ventromedial prefrontal cortex grey matter were 0.82 ± 0.09 V/m, ranging 0.62–0.99 V/m, and average intensity values were 0.46 ± 0.05 V/m, ranging 0.36–0.52 V/m. Of note, the channel configuration used for two TES^15 kHz^-TI^1 Hz^ participants differed by one channel (E94 instead of E95); however, their data was included given that electric field simulations for both configurations were highly similar.

### TES-TI

Four silver-silver chloride stimulating channels (12.6 mm outer diameter, 8.0 mm inner diameter, 2.2 mm width) with 1.5-m cable length were embedded into the hd-EEG net, attached to the participant’s scalp with conductive paste (Ten20), and then connected to the TI Brain Stimulator for Research (TIBS-R v3.0, TI Solutions AG, Switzerland). Electrode impedances were monitored throughout the experiment and served as an indicator for the need to adjust electrodes; substantial increases in impedance (to greater than 6500 Ohm) indicated poor positioning and a need for adjustment. Adjustments were targeted for when a participant entered a light stage of sleep (i.e., N1) or was awake. Adjustments consisted of applying additional Ten20 conductive paste and/or measures to improve electrode adherence to the scalp. Overall, adjustments were infrequent, as out of the 49 stimulation nights analyzed, only 4 nights had adjustments, which occurred one or two times during periods of wake. Average impedances were 4590 ± 110 Ohm for channel 1 and 5020 ± 600 Ohm for channel 2. TES-TI protocols during NREM sleep had the following parameters: 15,000 Hz carrier frequency, 1 Hz difference frequency, sinusoidal waveform, 5 mA zero-to-peak, 3-min duration with 15-s ramping up/down periods. Channel 1 used a 15,000 Hz frequency and channel 2 used a 15,001 Hz frequency. As a higher carrier frequency lowered impedance at the electrode-scalp interface, a 15,000 Hz carrier frequency was chosen to mitigate the possibility of sensations being felt by participants during sleep. Actual, average current delivered was 5.21 ± 0.21 mA for channel 1 and 5.06 ± 0.19 mA for channel 2 as reported by the TIBS-R device. Programming of the TIBS-R device was performed using its Python software.

### hd-EEG data acquisition

The hd-EEG system from MagStim Electrical Geodesics, Inc. (EGI) consisted of a hd-EEG net, an amplifier (NA 400), and a physiological input box. Each net had 256 silver-silver chloride electrodes and was appropriately sized to each individual participant. In cases where the stimulating electrodes were very close to the hd-EEG recording electrodes, e.g., small net sizes, surrounding recording electrodes were not gelled to avoid the possibility of bridging between stimulating and recording electrodes. Nets were then connected to EGI amplifiers that interface with EGI’s Net Station Acquisition software (v5.4.2) for real-time data visualization. All recording electrode impedances were kept below 50,000 Ohm, with the reference electrode below 5,000 Ohm, as measured at the start of the recording. The physiological input box was used to collect polysomnography data (electrooculography, electromyography, and electrocardiography). In addition, data from the TIBS-R trigger was input into the physiological input box. The optical trigger box output a 5 V square wave signal with the onset and offset of stimulation and, when synchronized to hd-EEG recordings, allowed for offline identification of stimulation intervals. All data were collected at a 500 Hz sampling rate. The total number of hours of EEG data acquired across the baseline, first, and last nights utilized in these analyses was 7.99 ± 0.75 hours per night per participant.

Hardware filters specifically designed for the TIBS-R device allowed for simultaneous TES-TI and hd-EEG recording. A low-pass filter (7th order, 100 Hz cutoff) was placed in the path of the hd-EEG amplifier input such that signals from the hd-EEG net were filtered before they reached the amplifier. A high-pass filter (4th order, 250 Hz cutoff) was placed in the path of the TIBS-R output such that intermodulation products and low-frequency noise generated by the TIBS-R were filtered before they reached the stimulating electrodes. Both hardware filters were used to allow for simultaneous TES-TI stimulation and hd-EEG recording.

### MRI data acquisition

MRI data were collected using a 3 Tesla MAGNUS (Microstructure Anatomy Gradient for Neuroimaging with Ultrafast Scanning, GE Healthcare) head-only MRI scanner. The head coil used was a 32-channel head radio frequency (RF) array (Nova Medical, Wakefield, MA). Structural images were acquired using T1- and T2-weighted images, with 0.8 mm isotropic voxels, and the following parameters. T1-weighted: sequence =  MP-RAGE (magnetization-prepared rapid gradient-echo), repetition time (TR) = 2000 ms, echo time (TE) = 3 ms, inverse time (TI) = 1100 ms, flip angle = 8 degrees, field of view (FOV) = 256 × 256 mm^2^, matrix size = 320 × 320 pixels, resolution = 0.8 mm × 0.8 mm × 0.8 mm, number of slices = 240, acquisition time = 4 min. T2-weighted: sequence = CUBE-T2, TR = 2500 ms, TE = 90 ms, echo train length (ETL) = 120, FOV = 256 × 256 mm^2^, matrix size = 320 × 320 pixels, resolution = 0.8 mm × 0.8 mm × 0.8 mm, number of slices = 240, acquisition time = 4 min. The structural MRI data were converted into NIfTI format using the open-source tool *dcm2niix*. Both T1- and T2-weighted images were defaced for all participants using the open-source Python-based defacing utility tool, *pydeface*.

### Sleep staging

Sleep staging was performed manually in accordance with the American Academy of Sleep Medicine guidelines and as follows by a registered polysomnographic technician^54^. Electrooculography, electromyography, and 6 hd-EEG channels that approximate 10:20 locations F3, F4, C3, C4, O1, O2, were used for staging. Hd-EEG data were filtered using MATLAB’s *filtfilt* function with a design: 4th order infinite impulse response filter band-passed 1–30 Hz. Electromyography data were filtered using MATLAB’s *filtfilt* function with design: 4th order infinite impulse response filter band-passed 1–100 Hz followed by a notch filter: 4th order infinite impulse response filter stop band 59–61 Hz. CountingSheepPSG (v1.4.5) MATLAB software was then used to perform staging in 30-s epochs and calculate sleep metrics, such as total sleep time, sleep onset latency (to Stage 1), wake after sleep onset, and others. The number of arousals and arousal index (number of arousals per hour) were quantified in line with AASM guidelines^54^. Number of awakenings was also included and refers to the number of times the stage transitioned from sleep to wake. Nonparametric statistical tests were used to evaluate for potential changes in these metrics across participant groups (Wilcoxon rank sum) and within participants, across intervention nights (Friedman test).

### hd-EEG preprocessing

hd-EEG data preprocessing was performed using MATLAB (v2023b) and EEGLAB (v2025.0.0)^55^. hd-EEG data were band-pass filtered 0.5–40 Hz using EEGLAB’s finite impulse response filter function, *pop_eegfiltnew*. Only 30-s NREM epochs staged 2 or 3 were included in spectral power analyses.

### hd-EEG artifact rejection

Semi-automated artifact rejection was performed to remove channels and epochs containing artifactual activity (e.g., high-frequency noise, interrupted contact with the scalp)^56–58^. Specifically, a fast Fourier transform was used to calculate spectral power in low (0.5–4 Hz) and high (20–30 Hz) frequency ranges for 6-s NREM epochs. For each channel, automatic thresholds were calculated at the 99th percentile and then plotted and visually inspected. If epochs with substantially greater low- or high-frequency power did not exceed the automatic threshold, the threshold was lowered. All epochs exceeding the threshold were then removed. Removed epochs (due to artifact or a sleep stage that was not NREM 2 or 3) were replaced with NaN values to maintain the temporal structure of the data, but were not included in the spectral power calculations.

Channels in which artifacts affected most of the recording were removed. Additional spectral- and topographic-based procedures were used to identify and remove channels with distinctly greater power relative to neighboring channels. On average, 74% (189 ± 23) of the 256-hd EEG channels remained on intervention nights. A final visual review of the hd-EEG data was performed to remove any remaining channels and epochs with artifactual activity.

To increase the signal-to-noise ratio, analyses were restricted to 185 scalp channels (excluding neck and face channels). Any removed channels, including channels that were not gelled, within the scalp 185 channels were interpolated using spherical spline interpolation via the *eeg_interp* function. All channel data was then re-referenced to the average of the final 185 channels. For visualization only, topographies were plotted using EEGLAB’s *topoplot* function with a decreased radius (plotrad = 0.51); all 185 channels were included in analyses.

### hd-EEG alignment

Timing of TES-TI throughout the night was determined based on hardware trigger signals in agreement with timestamps recorded at the start and end of individual stimulation protocols. Time periods included a baseline PRE stimulation period, 3-min prior to the start of stimulation, a STIM period including a 15-s ramp up, 3-min stimulation and 15-s ramp down and a POST stimulation period, 3-min after the ramp down. Analyses comparing STIM and PRE or POST intervals excluded the ramping interval to allow for equal 3-min comparisons.

### hd-EEG spectral power analyses

For each hd-EEG channel, the power spectral density (PSD) was calculated with Welch’s method using 6-s windows with 90% overlap for a 0.167 Hz step size. PSD values were then averaged over frequencies to calculate band power (e.g., SWA, 0.5–4 Hz). Band power was normalized to account for differences between participants and night-to-night variability within participants^59^. For each participant and each night, the band power of interest (e.g., SWA) for each channel in PRE, STIM, and POST periods was divided by the average band power (e.g., SWA) during NREM sleep throughout the entire night to obtain a relative change (shown as a percent) from the mean overnight band power^59^. Canonical frequency bands were defined as follows: SWA: 0.5–4 Hz, theta: 4–8 Hz, alpha: 8–12 Hz, low sigma: 9–12 Hz, high sigma: 12–16 Hz, beta: 16–25 Hz, and gamma: 25–40 Hz. The main analyses were also repeated using a narrower frequency band surrounding SWA, 0.5–2 Hz, and the results were qualitatively similar to the broader 0.5–4 Hz band.

### Statistics and reproducibility

Summary statistics for the main figures, including sample sizes and effect sizes (Cohen’s *d*), can be found in Supplementary Table 4.

#### Hypothesis testing

Descriptive statistics in the text are reported as mean ± standard deviation. Prior to performing any statistical tests, data were evaluated for normality using the Shapiro–Wilk test (*swtest* in MATLAB, v2023b). If data distributions were normal, parametric tests were used; if data distributions were not normal, nonparametric tests were used. All statistical tests were two-sided.

#### Correction for multiple comparisons

To assess for statistical significance in the context of multiple comparisons, the Benjamini–Yekutieli procedure was used to correct families of comparisons to a false discovery rate of 5% (*fdr_bh* in MATLAB). Comparisons were grouped into families based on conceptual similarity, and corrections were applied within each family. *P*-values reported in the text are adjusted according to this procedure; if the adjustment resulted in a number greater than 1, *p*-values are reported as *p* > 0.999. Both the original unadjusted *p*-values and the reported adjusted *p*-values can be found in Supplementary Table 4. For analyses that do not report adjusted *p*-values (e.g., age, sex, number of stimulation protocols, etc.), tests were conceptually isolated and not considered to be part of a family requiring correction for multiple comparisons.

#### Statistical power

Statistical power calculations were performed using the *pwr* function in R (v4.5.2) for t-tests with a two-sided alpha at 0.05 and using effect sizes observed in the sample. The primary findings that used data from participants that received the TES^15 kHz^-TI^1 Hz^ intervention were highly powered (e.g., Fig. 3, STIM-PRE, *N* = 21, Cohen’s *d* = 1.45, *β* = 0.99, *α* = 0.05, two-sided paired samples t-test). The comparison performed between TES^15 kHz^-TI^1 Hz^ participants and TES^15 kHz^ participants was moderately powered, but a significant difference was nevertheless detected with a large effect size (Fig. 4A, B, TES^15 kHz^-TI^1 Hz^ *N* = 21, TES^15 kHz^ *N* = 7, Cohen’s *d* = 1.08, *β* = 0.66, *α* = 0.05, two-sided independent samples t-test). Preliminary findings pertaining to only the TES^15 kHz^ group, given the small sample size, were included in the Supplementary Information; the results of the TES^15 kHz^ group analyses should be considered exploratory and hypothesis-generating rather than confirmatory.

#### Cluster-based statistics for topographic analyses

For topographic comparisons, a nonparametric cluster-based statistical approach using a suprathreshold cluster analysis to control for multiple comparisons was implemented^60–62^. For each permutation (*N* = 5000), new datasets were generated with randomly relabeled conditions from the original data. Then, a selected statistical test (e.g., t-test) was performed, and clusters were determined based on neighboring channels exceeding the critical value for a given two-tailed test distribution, degrees of freedom, and alpha of 0.05. The maximal size of resulting clusters was used to build a cluster size distribution across all permutations. The critical cluster size threshold was defined as the 95th percentile of this distribution. For the empirical comparison, only channels that exceeded the critical value and were located within a cluster larger than the critical cluster size threshold were considered significant^60,61^. The number of significant clusters, total number of significant electrodes, and mean *p*-value ($\bar{{{\rm{p}}}}$) of those significant electrodes are reported; channels with significant responses are shown as white dots in figures. If no significant clusters were identified, the mean *p*-value ($\bar{{{\rm{p}}}}$) across all electrodes is reported.

#### Linear mixed effects (LME) model for participant grouping

A linear mixed effects (LME) model was used to assess for any differences across subgroups that received the TES^15 kHz^-TI^1 Hz^ intervention. As described in ref. ^63^, the LME was fit with numerical maximum likelihood estimation using the *lmer* function of the *lme4* package in R. In general, the model related the response variable (*y*) to experiment and the covariate of interest (*x*) as fixed effects with an interaction term, and participant as a random effect (that is, “M1 = *y* ~ *x* × experiment + (1|participant)”). We then tested the hypothesis that there was no interaction between *x* and the experiment by fitting a model with the interaction term removed (that is, “M2 = *y* ~ *x* + experiment + (1|participant)”) and performing an asymptotic likelihood ratio test to compare the full and reduced models. In all cases, residual plots were examined for evidence against the assumptions of constant variance and normality. If no significant interaction was found, we tested for a main effect of the primary covariate by fitting a model with that term removed (that is, “M3 = *y* ~ experiment + (1|participant)”) and performed an asymptotic likelihood ratio test to compare this model with the one including the covariate (“M2”). Cohen’s *f* ^2^ was used as a measure of effect size and was calculated as in ref. ^63^.

Specifically, for these analyses, SWA was the response variable, and TES^15 kHz^-TI^1 Hz^ subgroup (Groups 2, 3, or 4) and period (PRE, STIM, and POST) were fixed effects. Two separate models were applied, one for the first night and one for the last night.

#### Least absolute shrinkage and selection operator (LASSO) model for sleep quality

A predictive modeling approach was used to identify potential factors associated with REST-Q sleep quality ratings. Specifically, a regression analysis was performed using least absolute shrinkage and selection operator (LASSO) estimation with 5-fold cross-validation to select model hyperparameters (*glmnet* function in R). The LASSO is a regularization technique that drives coefficient estimates to zero for marginal inputs, resulting in a sparse regression that identifies which model inputs improve the prediction of the model outputs. Power averaged across all channels in SWA, theta, beta, low sigma, and high sigma bands during STIM-PRE and POST-PRE periods (independent variables) were used as predictors for overall REST-Q ratings (dependent variable). Models were fit on first and last night data as well as the difference between last and first nights to assess for longitudinal changes within-participant. For the models, *λ*_min values are reported as they represent the level of regularization that minimized cross-validated prediction error. Mean squared error (MSE) for the held-out test folds is also reported. To visualize potential predictive relationships, linear regression models were used, and the line of best fit (*y* = *β*_1_ × *x* + *β*_0_) and *R*^2^ values are reported. To explore whether the predictive power in the observed data was spatially localized, for descriptive purposes, *R*^2^ values from the linear regression models were plotted topographically. The predictive modeling approach does not use the null hypothesis testing framework; instead, a training-testing split of the data via cross-validation plays the role of preventing overfitting and ensuring that the results for each model cannot be explained by random variation. Nevertheless, in the context of multiple models, findings should be considered preliminary rather than confirmatory.

## Results

**Fig. 2:**
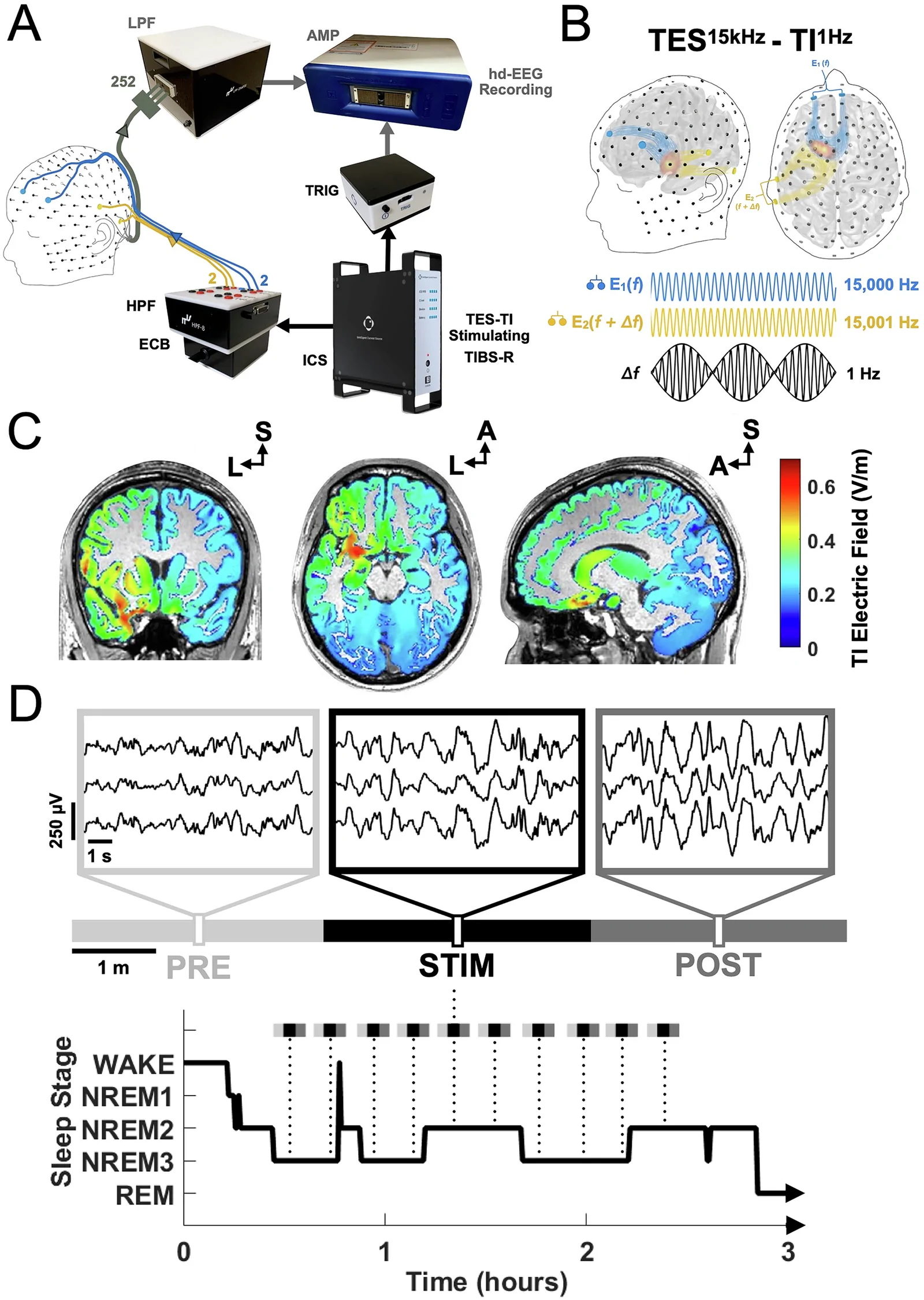
TES-TI overview. **A** The transcranial electrical stimulation with temporal interference (TES-TI) system was comprised of the temporal interference brain stimulator for research (TIBS-R) with components including the intelligent current source (ICS), electrode connector box (ECB), and high-pass filter (HPF). Current was delivered through two pairs of stimulating electrodes (four electrodes total) embedded in the hd-EEG net. All other hd-EEG recording channels (252) passed through a low-pass filter (LPF) before reaching the recording amplifier (AMP). The dual HPF and LPF system allowed for simultaneous TES-TI and hd-EEG recording. Trigger (TRIG) outputs from the TIBS-R allowed for the identification of when stimulation occurred. **B** TES^15 kHz^-TI^1 Hz^ consisted of one pair of electrodes delivering high frequency alternating current (*E*_1_: 15,000 Hz) and the other pair delivering high frequency alternating current with an offset (*E*_2_: 15,001 Hz) to create amplitude modulation at the difference frequency (1 Hz) deep in the brain. **C** Simulated temporal interference (TI) electric field in grey matter for an individual participant. Maximum TI electric field values occurred in deep, left ventromedial prefrontal cortical regions with values exceeding 0.7 V/m. **D** (Top) A single stimulation protocol with three-minute periods before (PRE), during (STIM), and after (POST) stimulation. Three channels of example EEG data during 10 s within each 3-min period shown. (Bottom) Representative example of the set of stimulations during NREM sleep, with ten stimulations occurring within the first hours after sleep onset.

Twenty-eight participants (30.5 ± 9.9 years of age, 61% (*N* = 17) female, *N* = 21 TES^15 kHz^-TI^1 Hz^, *N* = 7 TES^15 kHz^) from the first five cohorts of the STRENGTHEN clinical trial were included in the current study. As described in the “Methods,” participants were assigned to one of four groups, receiving interventions over the course of 4 weeks (Fig. 1A, B). Twenty-one participants (Groups 2, 3, and 4) received TES^15 kHz^-TI^1 Hz,^ and seven other participants (Group 1) received TES^15 kHz^ (Fig. 1B, C, and Supplementary Fig. 1). All participants had a baseline overnight with polysomnography (PSG) and hd-EEG recordings. Afterwards, overnight interventions consisted of PSG, hd-EEG, and stimulation. Hardware filters allowed for simultaneous hd-EEG recordings and stimulation (Fig. 2A). TES^15 kHz^-TI^1 Hz^ protocols performed during NREM sleep had the following parameters: 15,000 Hz carrier frequency, 1 Hz difference frequency, sinusoidal waveform, 5 mA zero-to-peak, 3-min duration with 15-s ramping up/down periods (Fig. 2B, D). Electric field modeling was used to target the left ventromedial prefrontal cortical region. The montage chosen (channel 1: E34–E20, channel 2: E70–E95) generated a theoretical electric field in which the amplitude-modulated (temporal interference) field exceeded 0.7 V/m in the target region when simulated for a representative participant (Fig. 2C). Across all TES^15 kHz^-TI^1 Hz^ participants (*N* = 21), maximal intensity values in left ventromedial prefrontal cortex grey matter were 0.82 ± 0.09 V/m, ranging 0.62–0.99 V/m. Average intensity values were 0.46 ± 0.05 V/m, ranging 0.36–0.52 V/m. Stimulations occurred in three-minute protocols repeated ten times during NREM sleep, identified online by sleep technicians (Fig. 2D). For most nights, stimulations were completed within the first four hours from sleep onset, as endogenous SWA is typically high during this timeframe (Fig. 2D). Stimulation parameters were the same across groups except that TES^15 kHz^ had a difference of 0 Hz.

Baseline, first, and last intervention nights were included in the current study. In total, twenty-two first nights (17 TES^15 kHz^-TI^1 Hz^, 5 TES^15 kHz^) and twenty-seven last nights (20 TES^15 kHz^-TI^1 Hz^, 7 TES^15 kHz^) were used in these analyses (Supplementary Fig. 1A). Analyses comparing baseline, first, and last intervention nights were restricted to only participants with usable data on all nights (Supplementary Fig. 1B, 16 TES^15 kHz^-TI^1 Hz^, 5 TES^15 kHz^).

Across all participants, the age range was 19–49 years, race was 85.7% White, 14.3% Asian, and ethnicity was 96.4% non-Hispanic, 3.6% Hispanic, with no statistically significant differences in age or sex between TES^15 kHz^-TI^1 Hz^ and TES^15 kHz^ (Table 1). Sleep characteristics during the baseline night also did not differ between TES^15 kHz^-TI^1 Hz^ and TES^15 kHz^, indicating adequate allocation of participants between groups (Table 2).

**Table 1:** Basic demographics

	TES^15 kHz^ - TI^1 Hz^	TES^15 kHz^	Test statistic (*W* or *T*), *p*-value
Number of participants	21	7	
Age (yrs)	31.5 ± 10.0	27.4 ± 9.8	(*W*) 319.5, 0.441
Sex (% Female)	57.1	71.4	(*X*^2^) 0.45, 0.503

TES^15 kHz^-TI^1 Hz^ and TES^15 kHz^ participants show no statistically significant differences in age (two-sided Wilcoxon rank sum test) or sex (two-sided chi-squared test). Mean ± standard deviation.

**Table 2:** Baseline sleep characteristics

	TES^15 kHz^ - TI^1 Hz^	TES^15 kHz^	Test statistic (*W* or *T*)	*p*-value
Total sleep-wake period (hr)	8.18 ± 0.94	7.62 ± 1.10	(*W*) 328.0	0.222
Total sleep time (hr)	6.83 ± 0.87	6.52 ± 1.25	(*W*) 311.5	0.730
Sleep onset latency (min)	23.10 ± 11.92	25.93 ± 14.00	(*W*) 293.5	0.577
REM latency (hr)	1.97 ± 0.70	2.42 ± 0.53	(*W*) 272.0	0.089
Sleep efficiency (%)	83.81 ± 8.58	85.49 ± 10.85	(*W*) 286.0	0.340
Number of arousals	64 ± 36	62 ± 53	(*W*) 320.0	0.426
Arousal index (arousals/hr)	9.29 ± 4.98	9.46 ± 7.28	(*W*) 314.0	0.633
Number of awakenings	21 ± 9	17 ± 13	(*W*) 332.0	0.151
Wake after sleep onset (min)	57.71 ± 45.11	39.64 ± 37.23	(*W*) 326.0	0.265
Stage N1 (%)	4.63 ± 2.89	4.39 ± 4.52	(*W*) 320.0	0.426
Stage N2 (%)	60.58 ± 9.00	61.11 ± 7.03	(*W*) 298.0	0.750
Stage N3 (%)	14.00 ± 6.03	14.33 ± 5.64	(*W*) 301.0	0.874
Stage REM (%)	20.79 ± 5.34	20.17 ± 3.89	(*W*) 312.0	0.710
Whole-night SWA (dB/Hz)	12.28 ± 2.41	11.72 ± 2.37	(*T*) 0.53	0.598
First 4 hr SWA (dB/Hz)	12.17 ± 2.69	11.74 ± 2.55	(*T*) 0.37	0.717

TES^15 kHz^-TI^1 Hz^ (*N* = 21) and TES^15 kHz^ (*N* = 7) participants show no statistically significant differences in any sleep characteristics during the baseline night (two-sided Wilcoxon rank sum test). Mean ± standard deviation. Unadjusted *p*-values shown; when corrected for multiple comparisons using the Benjamini–Yekutieli procedure, all adjusted *p*-values were > 0.999.

*REM* rapid eye movement, *SWA* slow wave activity.

### TES^15 kHz^-TI^1 Hz^ preserves sleep architecture

We compared sleep architecture metrics across baseline, first, and last intervention nights for those TES^15 kHz^-TI^1 Hz^ participants with usable data on all nights (Table 3, TES^15 kHz^-TI^1 Hz^ *N* = 16, Friedman tests, *X*^2^, d*f* = 2). There is a weak main effect of night on total sleep time; however, it is not significant after correction for multiple comparisons (*X*^2^ = 6.12, unadjusted *p* = 0.047, adjusted *p* > 0.999). There are no significant differences in any other sleep characteristics. Notably, there are no differences in metrics related to wake, including the number of arousals, arousal index, number of awakenings, or time spent awake after sleep onset. In addition, participants did not report feeling the stimulation during the night.

**Table 3:** Sleep characteristics across nights in TES^15 kHz^-TI^1 Hz^

	Baseline night	First night	Last night	Test statistic (*X*^2^ or *F*)	*p*-value
Total sleep-wake period (hr)	8.06 ± 0.99	8.25 ± 0.85	8.12 ± 0.78	(*X*^2^) 0.88	0.646
Total sleep time (hr)	6.70 ± 0.83	7.04 ± 0.69	6.92 ± 0.79	(*X*^2^) 6.12	0.047^†^
Sleep onset latency (min)	22.97 ± 11.89	23.44 ± 13.38	21.50 ± 15.39	(*X*^2^) 2.51	0.285
REM latency (hr)	1.86 ± 0.70	2.06 ± 0.76	1.93 ± 0.77	(*X*^2^) 3.50	0.174
Sleep efficiency (%)	83.62 ± 8.89	85.65 ± 6.65	85.33 ± 7.27	(*X*^2^) 1.12	0.570
Number of arousals	57 ± 26	69 ± 34	64 ± 31	(*X*^2^) 1.24	0.538
Arousal index (arousals/hr)	8.54 ± 3.95	9.80 ± 4.60	9.42 ± 5.12	(*X*^2^) 0.88	0.646
Number of awakenings	21 ± 7	21 ± 8	20 ± 8	(*X*^2^) 1.76	0.414
Wake after sleep onset (min)	58.19 ± 47.17	48.56 ± 36.60	50.13 ± 36.78	(*X*^2^) 2.13	0.345
Stage N1 (%)	4.07 ± 1.85	3.96 ± 2.94	4.07 ± 3.71	(*X*^2^) 0.38	0.829
Stage N2 (%)	61.49 ± 9.83	61.88 ± 5.76	60.03 ± 5.65	(*X*^2^) 0.38	0.829
Stage N3 (%)	13.75 ± 6.40	13.50 ± 6.35	12.62 ± 6.31	(*X*^2^) 0.88	0.646
Stage REM (%)	20.70 ± 5.73	20.66 ± 2.91	23.28 ± 3.79	(*X*^2^) 1.62	0.444
Whole-night SWA (dB/Hz)	12.34 ± 2.71	12.12 ± 2.66	12.21 ± 2.69	(*F*) 1.16	0.327
First 4 hr SWA (dB/Hz)	12.54 ± 2.69	12.37 ± 2.75	12.45 ± 2.90	(*F*) 0.50	0.611

Across baseline, first, and last nights, TES^15 kHz^-TI^1 Hz^ (*N* = 16) participants show no significant differences in any sleep characteristics after correction for multiple comparisons (Friedman test or one-way repeated measures ANOVA, Benjamini–Yekutieli procedure to correct for multiple comparisons). Mean ± standard deviation. Unadjusted *p*-values shown; when corrected for multiple comparisons, all adjusted *p*-values were > 0.999.

*REM* rapid eye movement, *SWA* slow wave activity.

^†^Total sleep time is not significant after correction for multiple comparisons (*X*^2^ = 6.12, unadjusted *p* = 0.047, adjusted *p* > 0.999).

SWA (power spectral density, PSD in 0.5–4 Hz range) was quantified throughout each entire night, then averaged across channels for each participant. There is no main effect of night (baseline, first, or last) on whole-night SWA (Table 3, TES^15 kHz^-TI^1 Hz^: *N* = 16, one-way repeated measures ANOVA, F, d*f* = 2). Similar results were found when SWA was measured during the first four hours after sleep onset; this early SWA did not differ between baseline, first, or last nights (Table 3). Additionally, the number of stimulation protocols completed during NREM sleep did not differ between first and last nights (first = 10 ± 2, last = 9 ± 1, *W* = 29.0, *p* = 0.910; two-sided Wilcoxon signed rank test). These comparisons were also repeated for the TES^15 kHz^ group, but given the small sample size, results should be considered exploratory and hypothesis-generating (Supplementary Table 1).

### SWA increases during and after TES^15 kHz^-TI^1 Hz^

**Fig. 3:**
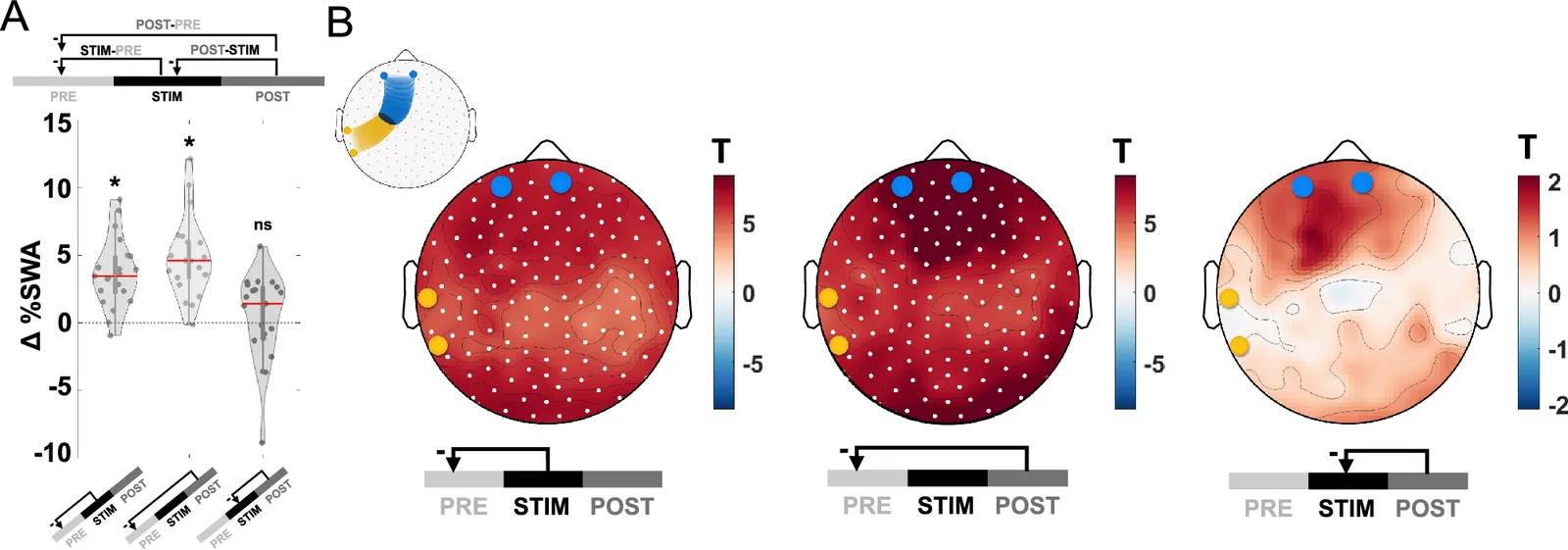
TES^15 kHz^-TI^1 Hz^ effects on SWA. **A** Across all channels, normalized SWA (%SWA) increases during and after TES^15 kHz^-TI^1 Hz^ compared to before (*N* = 21; STIM-PRE, *t* = 6.64, *p* = 4.97 × 10^‒6^, *d* = 1.45; POST-PRE, *t* = 7.46, *p* = 1.86 × 10^‒6^, *d* = 1.63). %SWA does not change after stimulation compared to during stimulation (POST-STIM, *t* = 0.63, *p* = 0.982, *d* = 0.14). Two-sided paired samples t-tests, Benjamini–Yekutieli procedure to correct for multiple comparisons. Median shown as a solid red line. **B** Increases in %SWA during and after stimulation are global. No channels have statistically significant changes in %SWA after stimulation compared to during stimulation. Topography of T-statistic shown; channels with statistically significant changes are indicated by white dots. Approximate locations of stimulating electrodes are shown in blue (*E*_1_) and yellow (*E*_2_).

We measured SWA in the immediate 3-min before (PRE), during (STIM), and after (POST) TES^15 kHz^-TI^1 Hz^ stimulation and calculated the differences between time periods (Fig. 3A). SWA was normalized (%SWA) to account for SWA differences between participants and night-to-night variability within participants (see “Methods,” hd-EEG spectral power analyses). Since SWA changes were consistent on the first and last intervention nights (Supplementary Fig. 2), we averaged $\Delta \%$SWA across the two nights for each participant (Supplementary Fig. 1). We also included participants (*N* = 5) with only one intervention night of usable data to increase statistical power.

Overall, we find a significant difference in the all-channel average SWA across PRE, STIM, and POST periods (*N* = 21, d*f* = 2, *F* = 30.95, *p* = 7.54 × 10^‒9^, one-way repeated measures ANOVA). We find a global (across all channels) increase in SWA during the stimulation as compared to before stimulation (Fig. 3A, STIM-PRE, *N* = 21, *t* = 6.64, *p* = 4.97 × 10^‒6^, *d* = 1.45; two-sided paired samples t-test). A global increase in SWA is also present after stimulation compared to before stimulation (Fig. 3A, POST-PRE, *N* = 21, *t* = 7.46, *p* = 1.86 × 10^‒6^, *d* = 1.63; two-sided paired samples t-test). There are no significant differences between the periods after (POST) stimulation and those during (STIM) stimulation in the average across all channels (Fig. 3A, POST-STIM, *N* = 21, *t* = 0.63, *p* = 0.982, *d* = 0.14, two-sided paired samples t-test). To identify statistically significant topographic changes at the level of individual channels, we implemented a nonparametric cluster-based statistical approach using a suprathreshold cluster analysis to control for multiple comparisons. The results of this topographical analysis confirm the results at the global level, i.e., SWA increases across all channels during stimulation and the increase persists to a similar degree after, with no significant differences between the STIM and POST periods, despite a higher T-statistic in frontal regions in the POST period (Fig. 2B, STIM-PRE: 1 positive cluster, 185 significant electrodes, $\bar{{{\rm{p}}}}$ = 1.92 × 10^‒5^; POST-PRE: 1 positive cluster, 185 significant electrodes, $\bar{{{\rm{p}}}}$ = 6.98 × 10^‒6^; POST-STIM: 0 clusters, 0 significant electrodes, $\bar{{{\rm{p}}}}$ = 0.538). A similar pattern of increases during and after stimulation are found when using a narrower frequency band surrounding SWA, 0.5–2 Hz (Supplementary Fig. 3A). Analyses were also repeated for the TES^15 kHz^ group, but given the small sample size, results should be considered exploratory and hypothesis-generating (Supplementary Fig. 4).

To investigate potential differences across subgroups that received the TES^15 kHz^-TI^1 Hz^ intervention (Groups 2, 3, and 4), a linear mixed effects model (LME) was applied for the first and last nights separately. SWA was the response variable, TES^15 kHz^-TI^1 Hz^ subgroup, and period (PRE, STIM, and POST) were fixed effects. We find no significant interaction between subgroup and period (First: *f* ^2^ = 0.16, *p* = 0.079; Last: *f* ^2^ = 0.07, *p* = 0.571) and find no main effect of subgroup (first: *f* ^2^ = 0.23, *p* = 0.482; last: *f* ^2^ = 0.11, *p* = 0.709). Importantly, effects are moderate and nonsignificant on the last night, when subgroup assignment may have theoretically had the greatest impact. Overall, these results indicate that changes in SWA during and after stimulation are similar across subgroups and support the decision to pool their data. Further supporting this approach is that a main effect of period was found for both first and last nights (first: *f* ^2^ = 0.90, *p* = 1.42 × 10^‒5^; last: *f* ^2^ = 0.84, *p* = 2.66 × 10^‒6^). Thus, the qualitative conclusions are the same—that SWA increases during and after TES^15 kHz^-TI^1 Hz^ stimulation—whether the subgroups are factored in with the LME or whether the subgroups are aggregated.

### TES^15 kHz^-TI^1 Hz^ effects on SWA during stimulation

**Fig. 4:**
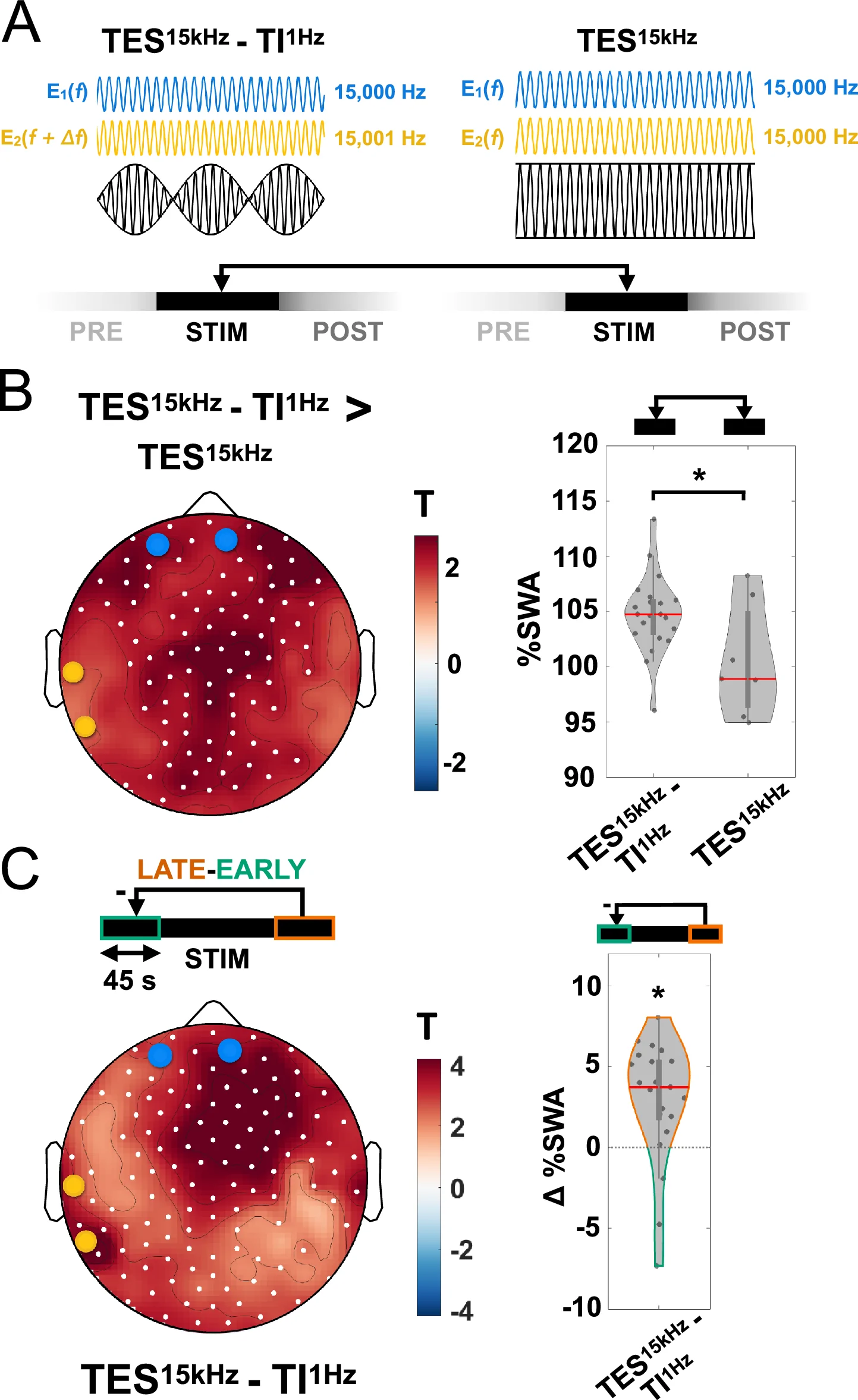
TES^15 kHz^-TI^1 Hz^ effects on SWA during stimulation. **A** In TES^15 kHz^-TI^1 Hz^, one pair of electrodes delivers high frequency alternating current with an offset (15,001 Hz) to create temporal interference (amplitude modulation) at 1 Hz. In TES^15 kHz^, both pairs of electrodes deliver the same high frequency alternating current, with no offset, i.e., there is no amplitude modulation (difference is 0 Hz). Normalized SWA (%SWA) during the stimulation period (STIM) is directly compared between TES^15 kHz^-TI^1 Hz^ and TES^15 kHz^. **B** (Left) During stimulation, %SWA is increased across widespread channels in TES^15 kHz^-TI^1 Hz^ as compared to TES^15 kHz^. (Right) Average %SWA across channels with statistically significant differences between TES^15 kHz^-TI^1 Hz^ and TES^15 kHz^ is shown. %SWA is higher during TES^15 kHz^-TI^1 Hz^ as compared to TES^15 kHz^ (TES^15 kHz^-TI^1 Hz^ *N* = 21, TES^15 kHz^ *N* = 7, *t* = 2.47, *p* = 0.020, *d* = 1.08, two-sided independent samples t-test). Median shown as a solid red line. **C** Within the 3-min period during stimulation, EARLY and LATE 45 s intervals are considered; the difference in %SWA between the LATE and EARLY stimulation intervals are compared during TES^15 kHz^-TI^1 Hz^. (Left) %SWA is greater in the LATE interval compared to the EARLY interval during TES^15 kHz^-TI^1 Hz^. Increases in %SWA later in stimulation are global. (Right) Averaged across all channels with statistically significant increases, %SWA is greater in the LATE interval (*N* = 21, *t* = 3.47, *p* = 2.41 × 10^‒3^, *d* = 0.76, two-sided paired samples t-tests). Topography of T-statistic shown; channels with statistically significant changes are indicated by white dots. Approximate locations of stimulating electrodes are shown in blue (*E*_1_) and yellow (*E*_2_).

We then performed a comparison of the effects on SWA of TES with amplitude modulation (TES^15 kHz^-TI^1 Hz^, 1 Hz difference) and without (TES^15 kHz^, 0 Hz difference) during stimulation (Fig. 4A). To do so, we directly compared normalized SWA (%SWA) in the STIM period between TES^15 kHz^-TI^1 Hz^ and TES^15 kHz^. Using nonparametric cluster-based statistics, we find a broad set of channels with SWA that is significantly greater in TES^15 kHz^-TI^1 Hz^ as compared to TES^15 kHz^ (Fig. 4B, left, 1 positive cluster, 116 significant electrodes, $\bar{{{\rm{p}}}}$ = 0.028). In the average across these channels, SWA during TES^15 kHz^-TI^1 Hz^ is greater than SWA during TES^15 kHz^ (Fig. 4B, right, TES^15 kHz^-TI^1 Hz^ *N* = 21, TES^15 kHz^ *N* = 7, *t* = 2.47, *p* = 0.020, *d* = 1.08, two-sided independent samples t-test).

We also tested whether the effects on SWA changed throughout the STIM period during TES^15 kHz^-TI^1 Hz^. For this analysis, we compared the last 45 seconds (LATE) of the STIM period with the first 45 seconds (EARLY), including the 15-second ramping interval (Fig. 4C). During TES^15 kHz^-TI^1 Hz^, SWA is higher in LATE compared to EARLY, and these increases in LATE SWA are global and strongest in the frontal region (Fig. 4C, left, 1 positive cluster, 168 significant electrodes, $\bar{{{\rm{p}}}}$ = 9.31 × 10^‒3^; Fig. 4C, right, *N* = 21, *t* = 3.47, *p* = 2.41 × 10^‒3^, *d* = 0.76; two-sided paired samples t-test). Similar patterns suggesting increased SWA during TES^15 kHz^-TI^1 Hz^ as compared to TES^15 kHz^, and during LATE TES^15 kHz^-TI^1 Hz^ as compared to EARLY, are found when using a narrower frequency band surrounding SWA, 0.5–2 Hz (Supplementary Fig. 3B, C).

### TES^15 kHz^-TI^1 Hz^ effects on other frequency bands

**Fig. 5:**
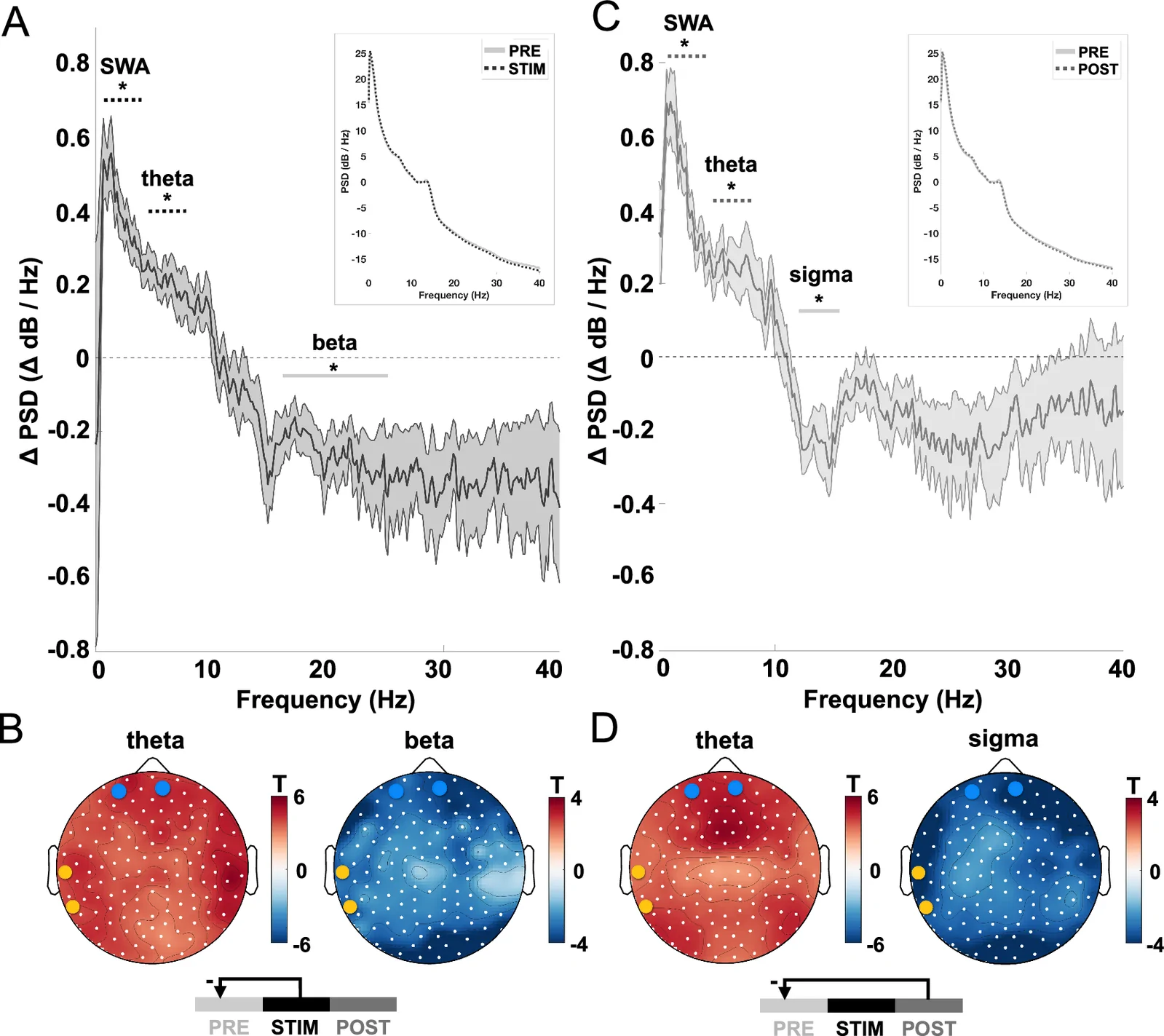
TES^15 kHz^-TI^1 Hz^ effects on other frequency bands. **A** Differences in power spectral density (PSD) during (STIM) compared to before (PRE) stimulation for individual frequencies (*N* = 240, 0.167 Hz steps). PSD is averaged across channels, then across participants (dark line), with the standard error of the mean across participants (light lines) shown. In addition to SWA (Fig. 2), increased theta and decreased beta power are found during stimulation (*N* = 21; theta: *t* = 4.28, *p* = 0.011, *d* = 0.93; beta: *t* = −3.30, *p* = 0.034, *d* = −0.72). Two-sided paired samples t-tests, Benjamini–Yekutieli procedure to correct for multiple comparisons. (Top right, inset) PSD for individual frequencies before (PRE) and during (STIM) stimulation. **B** Changes in normalized theta and normalized beta power are global. **C** Differences in PSD after (POST) compared to before (PRE) stimulation. In addition to SWA (Fig. 2), increased theta and decreased sigma power are found after stimulation (*N* = 21; theta: *t* = 3.89, *p* = 0.011, *d* = 0.85; sigma: *t* = −3.95, *p* = 0.011, *d* = −0.86). Two-sided paired samples t-tests, Benjamini–Yekutieli procedure to correct for multiple comparisons. (Top right, inset) PSD for individual frequencies before (PRE) and after (POST) stimulation. **D** Changes in normalized theta and normalized sigma power are global. Topography of T-statistic shown; channels with statistically significant changes are indicated by white dots. Approximate locations of stimulating electrodes are shown in blue (*E*_1_) and yellow (*E*_2_).

Next, we tested whether TES^15 kHz^-TI^1 Hz^ had additional effects on the EEG power spectrum. PSD values were averaged for each PRE, STIM, and POST period, then across electrodes, then across first and last intervention night, to yield one spectrum per participant (*N* = 21). Spectra were then averaged across participants (Fig. 5). For each frequency bin (*N* = 240, 0.167 Hz steps), the STIM-PRE difference, as well as the POST-PRE difference, were calculated. For statistical comparisons, for each canonical band (e.g., theta, alpha), PSD values were averaged across the frequencies in that band for each participant, then STIM-PRE and POST-PRE comparisons were performed using paired samples t-tests with correction for multiple comparisons (Supplementary Table 2). For selected significant topographical analyses, as was done with SWA, band power was normalized (% band power) by the average band power during NREM sleep throughout the entire night to give a relative change from the mean overnight band power. Then, the difference between time periods (STIM-PRE, POST-PRE) was evaluated with nonparametric cluster-based statistics.

We find that, as compared to before, during stimulation, TES^15 kHz^-TI^1 Hz^ increases power in the theta (4–8 Hz) range and decreases power in the beta (16–25 Hz) range (Fig. 5A, STIM-PRE, *N* = 21, theta: *t* = 4.28, *p* = 0.011, *d* = 0.93; beta: *t* = −3.30, *p* = 0.034, *d* = −0.72; two-sided paired samples t-tests). Both changes are global (Fig. 5B, theta: 1 positive cluster, 185 significant electrodes, $\bar{{{\rm{p}}}}$ = 8.97 × 10^‒4^; beta: 1 negative cluster, 173 significant electrodes, $\bar{{{\rm{p}}}}$ = 7.67 × 10^‒3^). As compared to before stimulation, after TES^15 kHz^-TI^1 Hz^, we find an increase in power in the theta range and a decrease in the sigma (12–16 Hz) range (Fig. 5C, POST-PRE, *N* = 21, theta: *t* = 3.90, *p* = 8.92 × 10^‒4^, *d* = 0.85; sigma: *t* = −3.95, *p* = 7.95 × 10^‒4^, *d* = −0.86; two-tailed paired samples t-tests). All these changes are also global (Fig. 5D, theta: 1 positive cluster, 185 significant electrodes, *p* = 1.35 × 10^‒3^; sigma: 1 negative cluster, 185 significant electrodes, $\bar{{{\rm{p}}}}$ = 4.44 × 10^‒3^). Analyses were also repeated for the TES^15 kHz^ group, but given the small sample size, results should be considered exploratory and hypothesis-generating (Supplementary Table 3).

### Changes in SWA during TES^15 kHz^-TI^1 Hz^ are associated with improved sleep quality ratings following the TES^15 kHz^-TI^1 Hz^ intervention

Finally, we examined potential relationships between stimulation-related changes in normalized SWA (%SWA) and participants’ perceptions of how they slept. For each participant in the TES^15 kHz^-TI^1 Hz^ group, we compared the difference in SWA during (STIM-PRE) and after (POST-PRE) TES^15 kHz^-TI^1 Hz^ with subjective sleep quality ratings from the restorative sleep questionnaire (REST-Q)^44,45^, assessed the morning after each intervention night. In line with Robbins and colleagues^44^, an overall REST-Q score was calculated for each participant and each intervention night.

To identify potential factors associated with overall REST-Q scores, we used a predictive modeling approach, specifically, LASSO estimation with 5-fold cross-validation. Power across all channels in SWA, theta, beta, low sigma, and high sigma bands during STIM-PRE and POST-PRE periods (independent variables) were used as predictors for overall REST-Q ratings (dependent variable). The model was fit on first and last night data as well as the difference between last and first nights to assess for longitudinal changes over the course of the intervention in the context of inter-individual variability.

**Fig. 6:**
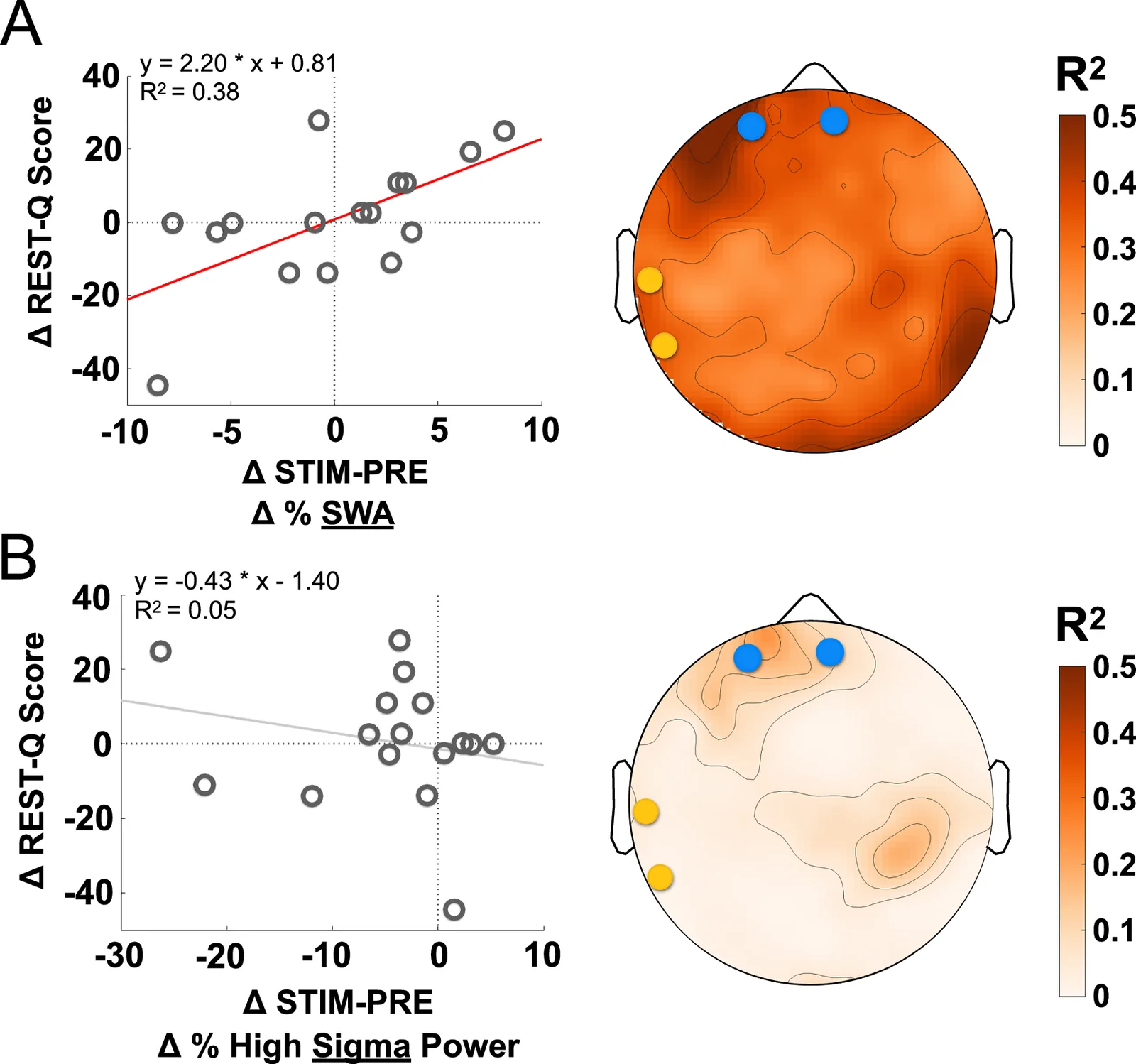
Relationship between changes in SWA during TES^15 kHz^-TI^1 Hz^ and changes in sleep quality following TES^15 kHz^-TI^1 Hz^ intervention. Greater increases in normalized SWA (%SWA), averaged across all channels, are related to improved sleep quality in TES^15 kHz^-TI^1 Hz^. **A** (Left) Difference in %SWA during stimulation (STIM-PRE, x-axis) is positively associated with the difference in overall REST-Q scores on the last intervention night as compared to the first intervention night (y-axis) (*N* = 16, *β*_1_ = 2.20, *R*^2^ = 0.38). (Right) Strongest relationships are present in the left frontal areas. The topography of *R*^2^ values is shown. **B** (Left) Difference in normalized 12–16 Hz (high sigma) power during stimulation (STIM-PRE, x-axis) is not associated with the difference in overall REST-Q scores on the last intervention night as compared to the first intervention night (y-axis) (*N* = 16, *β*_1_ = −0.43, *R*^2^ = 0.05). (Right) On topography, most areas have *R*^2^ values near zero. Approximate locations of stimulating electrodes are shown in blue (*E*_1_) and yellow (*E*_2_).

For first and last night data (*λ*_min = 2.98, cross-validated MSE = 313.0), no factors improve predictive performance. For the difference between last and first nights (*λ*_min = 9.44, cross-validated MSE = 298.6), only one factor improves predictive performance: the change in SWA during the STIM-PRE period. Specifically, this factor has a positive regression coefficient, indicating that greater increases in SWA during TES^15 kHz^-TI^1 Hz^ (STIM-PRE) on the last night compared to the first night are associated with higher overall REST-Q scores on the last night compared to the first night. To visualize the predictive model, we fit a simple linear regression model and extracted the line of best fit and *R*^2^ values (Fig. 6A, Left, *β*_1_ = 2.20, *R*^2^ = 0.38). To further investigate whether the predictive power in the observed data was spatially localized, *R*^2^ values are plotted topographically (Fig. 6A, right). We find relatively high *R*^2^ values globally, with the greatest values in the left frontal region. However, we note that these topographies are purely descriptive. Changes in sigma power (STIM-PRE) are shown as an example of a factor that does not improve predictive performance of the model (Fig. 6B, high sigma: 12–16 Hz, *N* = 16, *β*_1_ = −0.43, *R*^2^ = 0.05).

## Discussion

We performed simultaneous hd-EEG recording and TES-TI (15 kHz, 1 Hz difference) during overnight sleep in a laboratory setting in healthy humans with the goal of enhancing SWA during NREM sleep. Baseline, first, and last nights over the course of the 4-week STRENGTHEN study protocol were included in this interim report. The results show that TES^15 kHz^-TI^1 Hz^ targeting left ventromedial prefrontal cortical regions increased SWA during the stimulation period, and the effect outlasted the stimulation. TES^15 kHz^-TI^1 Hz^ during the stimulation period also increased SWA compared to TES^15 kHz^ without TI (15 kHz, 0 Hz difference), suggesting that the effect may be specifically attributable to TI. During TES^15 kHz^-TI^1 Hz^ stimulation, increases in SWA were greater in the late part of the stimulation interval. Effects on spectral power in other frequency bands were also found, pointing to an overall increase in low frequencies (SWA and theta range) and a decrease in higher frequencies (sigma and beta range) during and/or after stimulation. Lastly, participants who showed the largest incremental effects on SWA between the first and the last intervention night of the 4-week protocol also showed the largest improvement in restorative sleep ratings. Together, these findings support the ability of TES^15 kHz^-TI^1 Hz^ to enhance SWA and, more generally, the potential for TES-TI as an intervention to improve sleep.

TES-TI offers several potential advantages compared to other techniques for neuromodulation during sleep. While acoustic stimulation increases SWA^13,19,20,64^, the closed-loop titration of the intensity of stimuli to effectively trigger slow waves without awakening subjects can be problematic. This is likely because the triggering of slow waves is mediated by the non-lemniscal auditory pathway, which is associated with the ascending reticular activating system (ARAS) in the brainstem^13^. By directly modulating slow wave generation within their primary cortical origination sites, TES-TI circumvents unintended ARAS activation. Indeed, when comparing general sleep architecture metrics, which were comparable to^65^, across baseline, first, and last intervention nights, we found that TES^15 kHz^-TI^1 Hz^ did not affect the sleep architecture and, more specifically, did not increase the number of arousals, arousal index, number of awakenings, or the time spent awake after sleep onset. Notably, the percent of time spent in slow wave sleep (Stage N3) and SWA (both throughout the entire night and in the first 4 hours of sleep) did not change across baseline, first, and last intervention nights for TES^15 kHz^-TI^1 Hz^ participants. This is in line with several studies using an ON/OFF block design to enhance SWA with other methods^18^. It is possible that more numerous stimulation protocols and/or continuous stimulation might expand the increases in SWA observed in the immediate 3-minutes during and after TES^15 kHz^-TI^1 Hz^ to a larger timescale, over several hours. Also consistent with the preservation of sleep architecture was that the power spectra before, during, and after stimulation all maintained a profile consistent with NREM sleep, including a large, broad peak in the low frequencies corresponding to SWA and a smaller peak in the higher frequencies corresponding to sigma power.

Conventional TES, which typically employs frequencies below 1 kHz, has also been used to increase sleep SWA^15,19,66,67^. However, conventional TES is not well suited for reaching deep brain regions, and to do so focally, it produces scalp discomfort and potential awakenings at relatively low stimulation intensities (e.g., 1.5 mA). Also, electrical artifacts prevent the analysis of concurrent EEG recordings^18^. By contrast, TES^15 kHz^-TI^1 Hz^, as performed in this study, can reach deep brain regions with relatively high focality and field intensity, as indicated by electric field modeling of the interference field for individual subjects. Even at the relatively high intensities employed in this study (5 mA), TES^15 kHz^-TI^1 Hz^ did not produce any scalp sensation and did not awaken subjects. In addition, given the high carrier frequency (15 kHz, much higher than the EEG signal) and the use of dedicated hardware filters, it was possible to conduct simultaneous TES^15 kHz^-TI^1 Hz^ and hd-EEG recordings, avoiding stimulation artifacts (there were no narrow peaks at 1 Hz or its harmonics superimposed on the smooth power spectra typical of NREM sleep).

TES^15 kHz^-TI^1 Hz^ was intentionally targeted to a deep brain region—left ventromedial prefrontal cortical regions that we had previously identified as a “hot spot” for the origination of slow waves through source modeling of all-night hd-EEG recordings^40^. Because slow waves travel across the cortical surface^68^, we nevertheless suspected that the effects of targeted neuromodulation might extend to broader cortical areas. In fact, the increases in SWA during and after TES^15 kHz^-TI^1 Hz^ were global, extending across all channels.

An interesting aspect of the present results is that the increase in SWA produced by TES^15 kHz^-TI^1 Hz^ was incremental—increasing from the beginning to the end of the 3-min stimulation period, as SWA was higher in the last 45-seconds of stimulation compared to the first. Increases in SWA also outlasted the stimulation, extending to the subsequent 3 minutes. These findings suggest that TES^15 kHz^-TI^1 Hz^ at 1 Hz may not simply boost, synchronize, or entrain neuronal slow oscillations, but may produce plastic changes that underlie a cumulative effect. The extent of these changes may be more formally assessed by future studies that systematically vary the duration of the stimulation period. Overall, these findings clearly warrant further investigation of stimulation protocols that may maximize the effectiveness of SWA enhancement by lengthening stimulation periods, increasing the number of stimulation periods, and/or repeating them over subsequent nights.

An important part of this study was the direct comparison of TES^15 kHz^-TI^1 Hz^ and TES^15 kHz^. A separate group of participants (*N* = 7) received high frequency stimulation (15 kHz) with the same intensity and duration as the TES^15 kHz^-TI^1 Hz^ group, but with 0 Hz frequency difference between the two stimulation channels (TES^15 kHz^). High frequency TES is often assumed to produce minimal effects on the brain because of the dampening of the field intensity by the skull, resulting in subthreshold intensities, but this assumption has not yet been tested fully^39,69^. The present results showed that TES^15 kHz^-TI^1 Hz^, compared to TES^15 kHz^, increased SWA during the stimulation period. This initial finding supports the notion that the focal amplitude modulation at 1 Hz obtained through TES^15 kHz^-TI^1 Hz^ has specific effects on brain activity that are not observed by mere high frequency TES^15 kHz^, consistent with data from intracranial recordings^39^.

Besides increasing sleep SWA, the TES^15 kHz^-TI^1 Hz^ intervention employed here produced changes in the sleep power spectrum that were consistent with an overall deepening of sleep. In addition to increased power in the SWA (delta) band, we observed a global increase in theta power during and after TES^15 kHz^-TI^1 Hz^ stimulation. Sleep onset is typically accompanied by an increase in both delta and theta power^70,71^ and recovery sleep is characterized by increases in low frequency power that extend into the theta band^72–75^. An increase in theta activity continuous with an increase in SWA is an expected consequence of increased sleep pressure^2,3,72–75^. Similarly, the global decrease in beta power concomitant with increased SWA during TES^15 kHz^-TI^1 Hz^ was consistent with the inverse relationship between beta and delta power and the higher delta:beta ratio associated with reduced arousal levels^71,76^. The decrease in power in the sigma range (12–16 Hz) after TES^15 kHz^-TI^1 Hz^ (and a trend during TES^15 kHz^-TI^1 Hz^) may also reflect a deepening of sleep. In general, a reduction in spindles is seen with the transition to slow wave sleep (Stage N3), in recovery sleep after total sleep deprivation^72,75^ and, in particular, fast spindle power has dynamics that run in the opposite direction of those of SWA^77–79^. In addition, as supported by intracranial EEG in humans, maximal SWA accompanied by decreases in spindle density and frequency are most evident in earlier NREM sleep, consistent with when most stimulation protocols were performed in this study^80,81^.

A promising, albeit preliminary, indication of this study was the improvement in the restorative quality of sleep when comparing the first and last intervention nights of TES^15 kHz^-TI^1 Hz^, as reported by subjects through REST-Q scores. Specifically, participants with greater increases in SWA during TES^15 kHz^-TI^1 Hz^ from the first to the last night also reported better overall restorative sleep ratings. This relationship was specific to sleep SWA, as it did not extend to the other frequency bands tested (theta, beta, sigma). The ability of TES^15 kHz^-TI^1 Hz^ to produce effects on neuronal activity during sleep that outlast the stimulation and may be associated with plastic changes, as identified here, will be further investigated in the STRENGTHEN study by assessing long-lasting modifications in brain anatomical and functional connectivity, as well as in behavioral indicators of cognitive flexibility and emotional regulation^82,83^.

This study of TES-TI during sleep had several limitations. We targeted the left ventromedial prefrontal cortex using a standardized montage for TES^15 kHz^-TI^1 Hz^. Future work may determine whether more precise, individualized TES-TI targeting may offer superior efficacy^51,84^. Our initial analyses focused on the effects of TES^15 kHz^-TI^1 Hz^ during NREM sleep on first and last intervention nights. Future work within the STRENGHTEN framework will evaluate additional intervention nights as well as the effects of TES^15 kHz^-TI^6 Hz^ during REM sleep. Further enrollment will have increased the number of subjects receiving TES^15 kHz^ and possibly added a within-participant component. On the other hand, few clinical trials of TES-TI have provided a direct comparison with TES^39^, as was done here.

SWA varies with age, and the age range in this study is relatively wide (19–49 years). However, we assessed the effect of TES^15 kHz^-TI^1 Hz^ with an intra-subject design, by comparing adjacent 3-minute periods and normalizing power to the entire night^59,71,85,86^. While fluctuations of SWA during NREM episodes can present a challenge, the use of multiple stimulation blocks (repeated ten times per night) primarily throughout the first 4 hours of NREM sleep was intended to avoid spurious effects associated with infra-slow oscillations or the natural deepening of sleep. Future studies will aim at identifying the optimal parameters for TES-TI stimulation, including duration and timing, informed by increasing knowledge about its mechanism of action^22,26,29,30^. Moreover, the ability of TES-TI to modulate other features of sleep, from spindles to sawtooth waves to sharp-wave ripples, are also ready for exploration, with potential applications in health and disease.

## Conclusions

To our knowledge, this interim analysis of data from the STRENGTHEN study provides the first evidence in support of the ability of TES-TI (15 kHz, 1 Hz difference) targeting the left ventromedial prefrontal cortex to increase SWA during and after stimulation. Participants with the greatest longitudinal increases in SWA during TES-TI also have the greatest improvements in ratings of restorative sleep, potentially indicating beneficial effects of TES-TI on sleep.

## Supplementary information

Transparent Peer Review File

Supplementary Information

Description of Additional Supplementary Files

Supplementary Data 1

Supplementary Data 2

Supplementary Data 3

Supplementary Data 4

Supplementary Data 5

## Data Availability

Data needed to reproduce tables and figures for the main results are provided as Supplementary Data files. Specifically, Source data for Tables 1–3 are in Supplementary Data 1. Source data for Fig. 3 are in Supplementary Data 2. Source data for Fig. 4 are in Supplementary Data 3. Source data for Fig. 5 are in Supplementary Data 4. Source data for Fig. 6 are in Supplementary Data 5. All source data for Figs. 3–6, Tables, and Supplementary Tables and Figures are also publicly available via a generalist repository (https://doi.org/10.5281/zenodo.18653921) ref. ^87^. Access to the unprocessed data underlying this article is available upon reasonable request to the corresponding authors.

## Code availability

Customized codes used to create the main figures and tables have been made publicly available via a generalist repository (https://doi.org/10.5281/zenodo.18653921) ref. ^87^.
