# Supplementary material for: Enhancement of sleep slow wave activity using transcranial electrical stimulation with temporal interference: an interim analysis of the STRENGTHEN study
Source: Commun Med (Lond). 2026 Jul 1;6:549. doi: 10.1038/s43856-026-01744-x (PMC13687101; doi:10.1038/s43856-026-01744-x)
Supplement: Supplementary file 1 [file 43856_2026_1744_MOESM1_ESM.pdf]

nature portfolio

Peer Review File

Enhancement of Sleep Slow Wave Activity using Transcranial Electrical Stimulation with Temporal Interference: An Interim Analysis of the STRENGTHEN Study

Corresponding Author: Professor Giulio Tononi

This file contains all reviewer reports in order by version, followed by all author rebuttals in order by version.

Version 1:

Reviewer comments:

Reviewer #1

(Remarks to the Author)
Schaeffer et al. present an innovative study using temporal interference (TI) to enhance slow waves in 21 healthy participants.

TI is a novel technique, previously explored in areas such as epilepsy but not yet applied to sleep research. Its introduction to the field of sleep represents a highly exciting and important development. The study is carefully designed, well executed, and demonstrates robust results with a creative and rigorous methodology. I congratulate the authors on bringing this important method into the sleep domain and providing the first evidence of its positive effects.

Comments

The analysis focuses on delta power, but slow waves have additional defining features (e.g., peak-to-peak amplitude, negative peak amplitude, duration, and slope). Exploring these characteristics could provide further insight. Were such analyses performed, or was this beyond the scope of the current work?

The authors report that the post-stimulation period shows an increase in slow-wave activity comparable to the pre- and during-stimulation phases. In the discussion, they state that the study design does not allow them to determine how long this effect persisted. However, since full-night recordings were obtained, it is not entirely clear why the duration of the effect could not be further characterized. Could the authors clarify this point?

The observed reduction in sigma power is intriguing. Could the authors expand on this finding? Specifically, is this reduction related to spindle activity, and if so, were spindle counts or characteristics quantified?

The manuscript mentions screening participants, but it is not clear what was screened for. Were subjects evaluated for sleep disorders, including high apnea–hypopnea index (AHI)? Clarification on the screening process would be helpful.

The choice of a 15 kHz carrier frequency is briefly noted but not fully explained. Could the authors elaborate on the rationale for this selection and whether alternative frequencies were considered?

Minor Comment

The manuscript has some inconsistencies in font styles and sizes that should be corrected for uniformity.

Reviewer #2

(Remarks to the Author)
This manuscript evaluates transcranial electrical stimulation with temporal interference (TES-TI) using a 1-Hz offset to modulate slow-wave activity (SWA) during NREM sleep. Sleep architecture appears unchanged, whereas TES-TI transiently increases SWA and modulates other sleep-related frequency bands beyond stimulation periods. The approach is promising as a potentially more focal, deeper-reaching alternative to existing non-invasive methods, with fewer perceptual sensations that might disturb sleep. The study is thoughtfully designed and the results are very promising with clear implications for basic and clinical research. However, several aspects of the sample size justification and statistical analysis currently limit the strength of the conclusions and require additional clarification and robustness checks.

Major comments:
1. The control group size is justified using power calculations for paired, one-sided t-tests, yet the manuscript reports between-group comparisons (Fig. 3B). Power based on a paired, one-tailed test seems not appropriate for a between-subject contrast and likely underestimates the required sample size of the TES control group. One-sided testing also presupposes a clear directional hypothesis not specified in the preregistration (NCT06267521). I suggest to recalculate power based on the full TES-TI sample using two-sided α.
2. The correlation analyses shown in Fig. 5 raise some questions. Only changes from STIM–PRE between first and last intervention nights are correlated with REST-Q, whereas POST–PRE effects, that were reported for the two nights separately, are omitted but seem equally or more relevant as they are free of potential stimulation-period artifacts. Please, also specify whether multiple comparison corrections were applied. A single model could be applied with REST-Q change as the dependent variable, predictors including SWA and sigma power (low/high), Window (STIM–PRE vs POST–PRE), and relevant interactions.
3. The TES-TI sample was created by pooling three subgroups (Groups 2,3,4) that received heterogenous interventions. Some TES-TI participants practiced meditation while others did not. Given known associations between meditation and sleep quality (e.g., doi: 10.1016/j.smrv.2015.12.001), the authors consider including a Group factor (meditation vs no meditation) as predictor and test interactions with stimulation effects. If underpowered for full interaction testing, descriptive subgroup summaries may be provided and a sensitivity analysis acknowledging the limitation.
4. To further support the authors' main claim that temporal interference is the relevant component for SWA modulation, it may help to present POST–PRE effects for the TES-only group in parallel to Fig. 2 for TES-TI as supplementary information.
5. SWA is defined as 0.5–4 Hz, whereas TES-TI targets a 1-Hz envelope difference. Specifically strong effects confined to slow oscillations (SOs; ~0.5–1.25 Hz) would be theoretically expected. Please provide analyses for SOs (0.5–1.25 Hz) alongside SWA. If feasible, the authors may consider an event-level SO detection and to test whether SO characteristics change from PRE to POST.

Minor:
6. Fig. 3D suggests SWA increases from early to late STIM in TES-TI but not in TES.
Please also test the Group × (Early vs Late STIM) interaction directly.

7. The methods state that impedances were monitored and electrodes adjusted as needed. Please specify how adjustments were performed during sleep (criteria, timing relative to sleep stage, extent of manipulation) and whether any epochs were excluded due to adjustments.

8. It could strengthen the mechanistic interpretation to relate stimulation-specific metrics (electrode impedances, delivered current or simulated vmPFC field strengths) to physiological effects (SWA changes STIM–PRE and POST–PRE).

Reviewer #3

(Remarks to the Author)
This manuscript investigates the effects of Transcranial Electrical Stimulation with Temporal Interference (TES-TI) on sleep slow wave activity (SWA). The research question is both timely and of significant interest. However, I have substantial concerns regarding the analytical methodology employed in the study. These methodological flaws are significant enough to undermine the validity of the results and the conclusions drawn. Therefore, despite the novelty of the topic, I cannot recommend this manuscript for publication in its current form and must advise rejection.
The TES15kHz-TI1Hz condition in the manuscript combines subjects from three distinct groups: (Group 2) TES15kHz-TI1Hz (1 Hz difference frequency) administered 2 nights per week with meditation; (Group 3) TES15kHz-TI1Hz administered 1 night per week with meditation; and (Group 4) TES15kHz-TI1Hz administered 2 nights per week with sham meditation. When comparing this combined condition to Group 1 (TES15kHz administered 2 nights per week with meditation), the heterogeneity within the TES15kHz-TI1Hz group is substantial, which prevents isolating the pure effects of TES-TI. To address the research question more effectively, the authors should limit the analysis to only Groups 1 and 2.
The interventions described for each group in the manuscript differ from those listed in the clinical trial registration. The registration specifies four groups as follows: (1) meditation practice alone; (2) meditation practice combined with high-dose TES-TI; (3) meditation practice combined with low-dose TES-TI; and (4) TES-TI alone. Please clarify these inconsistencies and explain any deviations from the registered protocol.
The introduction should elaborate on the importance of slow-wave activity (SWA), discussing its relationships with sleep architecture and cognitive performance. Additionally, clarify whether the primary aim is to enhance sleep quality to improve cognitive outcomes.
As TES-TI is a relatively novel technique, the authors should provide a comprehensive yet accessible background, including its general effects, the rationale for selecting the carrier frequency, and the mechanism underlying the difference frequency.

The writing lacks academic rigor in several places. For example, in the first sentence of the main text, "EEG" appears without its full name (electroencephalography) on first use. In the abstract, it states that 21 subjects received TES-TI, whereas the study includes 28 participants overall. These issues should be corrected for clarity and consistency.
The manuscript reports multiple outcomes with varying sample sizes for comparisons, without applying corrections for multiple comparisons. This raises concerns about statistical validity and potential Type I errors. Please justify the approach or implement appropriate adjustments.
Provide a clear timeline indicating when baseline assessments were conducted relative to the interventions and follow-ups.
Different outcomes are analyzed using different sample sizes. Please explicitly detail these variations, including the number of participants from each of the four groups for every outcome measure.
The authors mention that data were derived from five cohorts, but the meaning of "cohorts" is unclear. Please define and explain what these cohorts represent, how they were recruited, and their relevance to the study design.

Reviewer #4

(Remarks to the Author)
In this high-density EEG (hd-EEG) sleep study, Schaeffer and colleagues investigated the effects of TES15 kHz-TI1Hz on sleep slow wave activity (SWA) in healthy participants, comparing their responses to those of healthy control subjects who underwent TES alone. The TES-TI group showed a significant increase in both sleep slow wave and theta activity, whereas the TES-only group did not. The authors suggest that TES15 kHz-TI1Hz can enhance sleep SWA and theta activity, while TES alone does not.

General comments:
The manuscript has many strengths. The study is well-motivated and theoretically sound, and the combination of hd-EEG with transcranial electric stimulation is highly sophisticated. The findings are interesting, novel, and contribute meaningfully to the existing literature.

I have some comments and suggestions, most of which are minor or moderate:

Introduction:
The introduction provides a strong theoretical background. No specific comments.

Sleep scoring:
1. Awakenings: According to AASM (2023), arousals should be reported as both the number of arousals and as the arousal index (number of arousals × 60/TST). It is unclear what is meant by “Number of awakenings” in Tables 2, 3, and Supplementary Table 1. Please use AASM-recommended terminology.
2. Sleep stage percentages: The percentages of sleep stages in Tables 2, 3, and Supplementary Table 1 deviate slightly from typical values reported in the literature (see e.g., Boulos et al., 2019, The Lancet Respiratory Medicine, 7(6), 533–543. https://doi.org/10.1016/S2213-2600(19)30057-8) and from clinical experience. The N1 stage appears reduced, and N2 increased, compared to typical values; N3 is on the lower side. Additionally, the number of awakenings (if this refers to arousals) is relatively high in Tables 2 and 3. These atypicalities raise questions about the scoring and suggest a need to clarify the quality of sleep scoring and the effects of TES-TI/TES on sleep architecture. Do the authors have an explanation for these discrepancies?
3. REM latency: REM latency is missing from Tables 2, 3, and Supplementary Table 1. If possible, please include this parameter, as it would be interesting for readers to see whether the stimulations (which partially occurred during the first NREM phase) delayed or advanced the onset of REM sleep.
4. Slow wave activity and arousals: How did the authors ensure that slow wave activity related to possible hypnopompic hypersynchrony (caused by brief arousals related to stimulation) did not confound the results?

Figure 1 / Experimental overview / Methods section:
The study presents data from baseline, first, and last intervention nights, with the first and last nights separated by a 4-week intervention protocol.
1. It would be helpful for readers if the durations of the recording (in minutes) for the pre-, during-, and post-stimulation periods were indicated in Figure 1.
2. What does the meditation intervention for 3out of 4 groups signify, and could it have affected the results?

Results / Number of subjects:
There was no main effect of night (baseline, first, or last) for SWA or for the first four hours in either group. Instead, SWA increased during and after TES-TI, but not in the TES-only condition. The TES-only group included 7 subjects (5 completed all three nights). While the statistical results for the TES-TI group are convincing, the authors should present calculations to justify the low number of subjects in the TES-only group, to assure readers that the sample size is sufficient to exclude false negative results.

Discussion:
The authors interpret the increase in theta power similarly to the increase in SWA. However, theta power is typical of N1 sleep, and this finding, together with changes in sigma power (i.e., sleep spindles, typical for N2 sleep), could also suggest lighter sleep (N2 → N1). Did the authors consider or wish to discuss the possibility that increases in SWA (deep sleep) were accompanied with some findings that might reflect lightening of sleep?

Reviewer #5

(Remarks to the Author)
Authors present a study that aims to improve slow wave activity (SWA) by using Transcranial Electrical Stimulation with Temporal Interference (TES-TI, 15 kHz, 1 Hz difference). They performed overnight studies in laboratory, and recorded high density electroencephalography (EEG) to measure SWA and other frequency bands. Findings reveal that SWA increase in left ventromedial prefrontal cortex during TES-TI stimulation period and 3-minutes after, compared to baseline and traditional TES. Largest SWA effect correlated to sleep improvement (questionnaires).

Overall, the study addresses an important question, if SWA could be promoted by using novel technology. Although TES has longer history, TES-TI provides new insights for the field, as authors repurpose it to promote SWA. The study was interesting indeed, and I read it with high curiosity. Somewhat I lost the red line during the reading, and did not follow, especially when thinking about the actual protocol and the reasons why it was chosen. Also high amount of numbers and p-values in the text were not convenient for reader. In general, I liked the figures a lot.

Key problems concern the presenting of the methods (how many laboratory nights, final subjects in the analysis, EEG processing, analysis, amount of data, starting and ending the recording, was there technician for the whole night following the data quality). During result and discussion parts of the text I had problems to follow, but also in the Methods chapter more details should be added.
Please, see my detailed comments below.

Abstract:
Please, define how many nights instead of “several nights”.
Introduction:
Authors could refer to novel suggestions that SWA enhancement could promote clearance of the brain. See review Hattem et al. 2025 doi: 10.1152/physiol.00019.2024
This would provide a direct link, that SWA improvement (also using TES-TI) could in future be tested as a therapy for prevention of Alzheimer’s or Parkinson’s disease.
Authors could open that TES-TI is the same as TIS (the term used in some studies) to make the terminology clear.
Results page 3:
Before figure 1, there should be a figure where a reader can clearly see the difference between groups, amount of laboratory nights and the full protocol in larger perspective than only one stimulation night as now presented in figure 1. Now it is really difficult to follow and try to understand the full picture of the study.
61% Female: Would provide this information as actual amount how many.
Why did you choose the protocols for groups 1-4? Why did you end up with the subject amounts of 21 and only 7 in TES group?
Page 4:
Please, explain the reason for the following and the reason why for some subjects it was not like that. “in most of the participants, stimulation were completed within the first four hours from sleep onset” As naturally there is more SWA during the first half of the night, this makes sense, to boost it but now it is not clear in the text, if this is the reason.
I still don’t get what happened between first and last nights: how many nights there actually were and why?
Fig1. Very nice!
Page 5:
It is hard to follow how many datasets were included in the what analysis and why.
Really hard to read statistical analyses and their results in the text.
I would use TES-TI and TES for abbreviations, to be more readable. As the frequencies are presented, and are always the same, this would be fine.
Page 7:
You say “SWA was normalized”. Please, open in the text what you mean e.g. total SWA power/total of all frequency powers?
“Overall, we found a significant difference in the average SWA” please, be clear with these, average about what in SWA? Also did you looked at the whole data, or cut the data? I realise that you got a lot of data in this study, but I think in the text, it should be more clear what part of the data you took, what channels you used, did you look at the same parts of the data for each subjects and so on, basically how did end up e.g. with the figures and the numbers that you got). As you were measuring the EEG for the whole nights, and for several nights, it was difficult to understand from which parts the actual figures were created.
Discussion:
Overall, I liked discussion a lot. In the first chapter of discussion, please refer to baseline, first night and last night, and wider look to the protocol. It should be clear that there were many laboratory nights per subject.
Study design page 16:
You mention here “daily meditation training” but I cannot see any other mention during the text about meditation. Please, provide detailed information about meditation training in this study.
Participants page 16:
“Participants were excluded if they had any neuroradiologist-identified structural abnormalities of the brain.” Did you exclude any? You say 28 participants were included, but did you exclude some before ending up to this amount?
Although supplementary material includes detailed information about about inclusion and exclusion criteria, manuscript would benefit if you add shortly what you mean by “Medically healthy”. I’m not also sure, will the supplementary material be published as it is or is it openly available. If yes, authors should refer to The Strengthen study protocol, so that readers can find the details there.

Excluding the sleep disorders with overnight laboratory setting was good. However, it was unclear whether this is the same night as baseline night for the subjects. And were some subjects excluded based on the exclusion night?
Figure about subject recruitment and exclusion would be helpful (could be the same chart where you include the excluded subjects based on the technical artifacts of the data).
Sleep quality surveys:
You could provide the information, why did you choose this REST-Q questionnaire.
hd-EEG page 18:
Please, provide detailed information about your EEG system (version), settings (e.g. was 0.1 Hz high-pass filtering on in recordings, electrode impedance limit). Also, you should mention somewhere how many hours of EEG-data was totally acquired (e.g. average+-std / night over subjects).
MRI Data Acquisition page 19:
Please, provide information what head coil was used.
Sleep staging page 19:
Why did you filter the data starting from 1 Hz and not 0.5 Hz as it is in American Academy of Sleep Medicine Guidelines for stage III delta waves? Please, shortly open in the text the criteria that used (e.g frequency ranges for different stages).
Hd-EEG preprocessing page 20:
How did you include “only” stage 2 and stage 3 epochs. Did you cut the data or do some other processing? Sometimes one stage can last only very short time (<30 s), so did you have some criteria that you only chose longer periods of the same sleep stage for analysis or what?

Reviewer #6

(Remarks to the Author)
In this paper, TES-TI is applied for the first time with the aim of enhancing SWA. This approach offers several advantages over previous work using TES: stimulation can be delivered without being consciously perceived, it allows for more precise targeting of specific brain regions, and it enables concurrent EEG analysis during stimulation, which was not feasible with TES. The authors report that TES-TI increases SWA both during the 3-minute stimulation period and in the subsequent 3-minute post-stimulation window compared to the pre-stimulation baseline. While these findings are interesting and the methodological innovation is noteworthy, I am not fully convinced by some of the reported effects, particularly the claim that these findings translate directly to sleep-restorative processes. My major concerns are outlined below.

1) Concerns regarding the control group (15 kHz stimulation group):
If I understand correctly, certain effects are not reported for this group:
• The increase in SWA during and after stimulation relative to the pre-stimulation window is described for the 15 kHz–1 Hz group, but it remains unclear whether this effect was also present (or absent) in the 15 kHz group.
• similarly, the reported correlation between the change in SWA increase (T1 to T2) and changes in sleep quality ratings (T1 to T2) is only mentioned for the 15 kHz–1 Hz group. Was this relationship tested for the 15 kHz group?

In addition, the 15 kHz group has a very limited sample size. Although the authors note that a power analysis suggested that 5–9 participants would suffice, I am not convinced that such a small group can reliably demonstrate the presence or absence of effects. And if such a small sample size is expected to yield meaningful effects, it would be important to clarify whether the effects reported for the full 15 kHz–1 Hz stimulation group are also observed when the original subgroups (2/meditation, 1/meditation, 2/sham meditation) are analyzed separately. Since these subgroups appear to have sample sizes comparable to the 15 kHz group, such an analysis would considerably strengthen the interpretation.

2) Potential confounding by original subgroup assignment:
In line with the previous point, I understand that for the baseline night and the first stimulation night (assuming neither meditation nor sham meditation had yet been administered) subgroup assignment is not relevant. However, since the majority of participants in the 15 kHz–1 Hz group appear to have undergone meditation training, I am concerned that effects observed on the last night may be substantially confounded. At a minimum, the statistical models should control for (a) whether participants received meditation training or not, and (b) whether they received 15 kHz–1 Hz stimulation once or twice per week.

3) Specificity of the correlation with subjective sleep ratings:
You report that the correlation with subjective sleep quality is specific to SWA, as no effect was observed for sigma power.
• How do the observed increases in theta power and decreases in beta power fit into this picture?
• I also find the rationale behind the change-score correlations somewhat unclear. What is the underlying hypothesis about what happens between the first and the last night (given that the night-intervention is the same) that would explain a change in how participants rate their sleep quality? Wouldn’t it also be expected that simple change scores of other sleep parameters - such as overall SWA, SWA in the first half of the night etc. - would correlate with changes in subjective sleep quality ratings? Clarifying whether such analyses were performed would help in evaluating the specificity of the reported effect.

4) Wording in the abstract:
The final sentence of the abstract should be phrased more cautiously. The reported effects on SWA did not extend beyond the stimulation window and the immediate post-stimulation period; therefore, it is not justified to conclude that sleep itself was enhanced. Similarly, it cannot be claimed that 15 kHz–1 Hz stimulation increases restorative processes, as the evidence is primarily based on correlations.

Minor:
- In the Methods section you state that all reported p-values are unadjusted unless otherwise specified. Could you please elaborate on the rationale for this choice?

Version 2:

Reviewer comments:

Reviewer #1

(Remarks to the Author)
No further comments.

Reviewer #2

(Remarks to the Author)
The authors have satisfactorily addressed all of my comments. As to my comment #2, they may optimize the revision concerning the LASSO regression, by reporting R^2 or MSE on the held-out test folds (not the full dataset) to assess whether SWA predicts sleep quality in new data, which is the purpose of the CV approach. Running the model 3 times (PRE,STIM,STIM-PRE) does reintroduce a multiplicity problem, just in a different form. Also, if I understand correctly, the authors are now topographically mapping the R2 values which is another implicit form of multiple comparisons (across electrodes). Or are the R^2 values in the topographic maps derived from the cross-validated model? Please clarify.

Reviewer #3

(Remarks to the Author)
The authors has addressed my comments effecttively. I have on further comments.

Reviewer #4

(Remarks to the Author)
I thank the authors for their thorough responses to all the points I raised. I have no further comments or concerns.

Reviewer #6

(Remarks to the Author)
The authors have addressed all my concerns appropriately and revised the manuscript accordingly. I recommend the manuscript for publication in its current form.

Version 3:

Reviewer comments:

Reviewer #2

(Remarks to the Author)
All points have been carefully addressed. In my view, the ms is ready for publication!

Open Access This Peer Review File is licensed under a Creative Commons Attribution 4.0 International License, which permits use, sharing, adaptation, distribution and reproduction in any medium or format, as long as you give appropriate credit to the original author(s) and the source, provide a link to the Creative Commons license, and indicate if changes were made.
In cases where reviewers are anonymous, credit should be given to 'Anonymous Referee' and the source.
The images or other third party material in this Peer Review File are included in the article's Creative Commons license, unless indicated otherwise in a credit line to the material. If material is not included in the article's Creative Commons license and your intended use is not permitted by statutory regulation or exceeds the permitted use, you will need to obtain permission directly from the copyright holder.
To view a copy of this license, visit https://creativecommons.org/licenses/by/4.0/

We thank the Reviewers for their time and careful consideration of our work. We have made substantial improvements based on your feedback, including concerns related to statistics by consulting with a statistician (additional author Dr. William Marshall). In line with his suggestions and those of the Reviewers, we have more clearly acknowledged the limitations of this work and tempered our conclusions accordingly.

In line with Reviewers' suggestions, we added one figure and three supplementary figures. In the following responses, we will denote the new figure # followed by the original figure # if applicable; for example, Figure 2. (original Figure 1.) refers to the TES-TI overview figure.

We would like to note a few issues we identified and corrected:

1. We found and corrected a minor technical issue that impacted the topography of band power in a subset of participants (N=7). The corrected figures and statistics have not meaningfully changed any of the original results.
2. The percentages listed in Table 1. Demographics for sex of TES-TI and TES participants were accidentally swapped. The values in the table have been correctly re-positioned. Please note this does not change the associated test statistic or p-value.
3. The Group numbering for which interventions were received by which groups did not match what was listed on the ClinicalTrials.gov website, as Groups 2 and 4 were swapped. We have changed the text so that the numbers align; in addition, we have added a new Figure 1. STRENGTHEN study overview that clarifies the groups. In the following responses, we refer to groups using the corrected numbers.
4. We decided to incorporate "TI (Temporal Interference)" into the "Electric Field (V/m)" label and legend for Figure 2. (original Figure 1.) to clarify that the electric field shown pertains only to the temporal interference component of stimulation.
5. The left panel of Figure 4B. (original Figure 3B.) now shows topographic differences that were previously reported only in text.

Lastly, additional changes in line with the Journal's checklist and CONSORT 2025 checklist have been incorporated.

Referee expertise:

Referee #1: Sleep Electrophysiology

Referee #2: Sleep; Transcranial Electrical Stimulation; Trial statistics

Referee #3: Neurostimulation

Referee #4: Clinical neurophysiology

Referee #5: Sleep dynamics

Referee #6: Sleep and stimulation

Reviewers' comments:

Reviewer #1 (Remarks to the Author):

Schaeffer et al. present an innovative study using temporal interference (TI) to enhance slow waves in 21 healthy participants.

TI is a novel technique, previously explored in areas such as epilepsy but not yet applied to sleep research. Its introduction to the field of sleep represents a highly exciting and important development. The study is carefully designed, well executed, and demonstrates robust results with a creative and rigorous methodology. I congratulate the authors on bringing this important method into the sleep domain and providing the first evidence of its positive effects.

Comments

The analysis focuses on delta power, but slow waves have additional defining features (e.g., peak-to-peak amplitude, negative peak amplitude, duration, and slope). Exploring these characteristics could provide further insight. Were such analyses performed, or was this beyond the scope of the current work?
We agree that the exploration of additional characteristics of slow waves is an exciting avenue that could provide further insight; however, as the Reviewer alluded to, performing such analyses with respect to the effects of our TES-TI paradigm is non-trivial and beyond the scope of the current work. However, we have started to do so and plan to present any results in future work.

The authors report that the post-stimulation period shows an increase in slow-wave activity comparable to the pre- and during-stimulation phases. In the discussion, they state that the study design does not allow them to determine how long this effect persisted. However, since full-night recordings were obtained, it is not entirely clear why the duration of the effect could not be further characterized. Could the authors clarify this point?
Thank you for raising this point. The sentence mentioned, that "because of the experimental design, we could not properly assess how long the enhancement may have lasted," is indeed not worded clearly. We meant that the duration of the effect was not a parameter directly assessed by this experimental design (i.e., that the 3-minute stimulation duration was a fixed parameter). We have removed the original sentence and more clearly stated need for future studies to directly address this parameter, as seen below.

- An interesting aspect of the present results is that the increase in SWA produced by TES^{15kHz}-TI^{1Hz} was incremental—increasing from the beginning to the end of the 3-minute stimulation period, as SWA was higher in the last 45-seconds of stimulation compared to the first. Increases in SWA also outlasted the stimulation, extending to the subsequent 3 minutes. ~~Because of the experimental design, we could not properly assess how long the enhancement may have lasted. Nevertheless,~~ These findings suggest that TES^{15kHz}-TI^{1Hz} at 1 Hz may not simply boost, synchronize, or entrain neuronal slow oscillations, but may produce plastic changes that underlie a cumulative effect. The extent of these changes may be more formally assessed by future studies that systematically vary the duration of the stimulation period. Overall, these findings clearly warrant further investigation of stimulation protocols that may maximize the effectiveness of SWA enhancement by lengthening stimulation periods, increasing the number of stimulation periods, and/or repeating them over subsequent nights.

The observed reduction in sigma power is intriguing. Could the authors expand on this finding? Specifically, is this reduction related to spindle activity, and if so, were spindle counts or characteristics quantified?
We agree that the reduction in sigma power is interesting. Our current interpretation is that the finding is in line with deepening of sleep, specifically the transition from N2 to N3, in which there is lower sigma power, spindle density, and spindle frequency as well as higher SWA. As identified from human intracranial EEG recordings, these dynamics are especially evident in

earlier NREM sleep [79-81], which is when most of our stimulation protocols were performed. While at the level of the thalamus, SWA and sigma power have been shown to follow a parallel time course, at the cortical level, as has been reported in many EEG studies, the two frequency bands demonstrate an inverse relationship within and across NREM episodes [79-81].
The reduction found was for sigma power, a proxy for spindle quantity and dynamics [a]; neither spindle counts nor characteristics were quantified. Like our rationale for slow wave detection, we found the analyses on individual spindles to add substantial time and be beyond the scope of the current work that emphasized SWA. We agree that analyzing spindle features will be an interesting area for future work.
We have added the following sentence to the Discussion and provided additional references.

- The decrease in power in the sigma range (12–16 Hz) after TES^{15kHz}-TI^{1Hz} (and a trend during TES^{15kHz}-TI^{1Hz}) may also reflect a deepening of sleep. In general, a reduction in spindles is seen with the transition to slow wave sleep (Stage N3), in recovery sleep after total sleep deprivation [72,75] and, in particular, fast spindle power has dynamics that run in the opposite direction of those of SWA [77-79]. In addition, as supported by intracranial EEG in humans, maximal SWA accompanied by decreases in spindle density and frequency are most evident in earlier NREM sleep, consistent with when most stimulation protocols were performed in this study [80,81].

79. Uchida, S., *et al.* Sigma (12-15 Hz) and delta (0.3-3 Hz) EEG oscillate reciprocally within NREM sleep. *Brain research bulletin* **27,** 93–96 (1991).
80. Andrillon, T., *et al*. Sleep spindles in humans: insights from intrancranial EEG and unit recordings. *J. Neurosci.* **31**, 17821-17834 (2011).
81. Sarasso, S., *et al*. Thalamic and neocortical differences in the relationship between the time course of delta and sigma power during NREM sleep in humans. *Journal of sleep research* **30**, e13166 (2021).
a. Fernandez, L. M. J., & Lüthi, A. (2020). Sleep Spindles: Mechanisms and Functions. *Physiological reviews*, *100*(2), 805–868.

The manuscript mentions screening participants, but it is not clear what was screened for. Were subjects evaluated for sleep disorders, including high apnea–hypopnea index (AHI)? Clarification on the screening process would be helpful.
Generally, screening consisted of questions regarding demographics, availability, medical and mental health, medications, and sleep. Participants with responses meeting exclusion criteria were not included in the study. As part of screening, for example, participants were asked about current or prior diagnoses of Obstructive Sleep Apnea (OSA); if yes, the participant was excluded. Full eligibility criteria can be found on the ClinicalTrials.gov website. As part of the pre-intervention baseline sleep visit, sleep technicians monitored for sleep quality. During the baseline sleep visit, two participants had loud snoring, breath holding and gasping accompanied by periods of wake. Given concern for OSA and overall poor sleep that would preclude experiment completion, these two participants were withdrawn after their baseline visit. While these concerns were not formally evaluated using the apnea-hypopnea index, we erred on the side of caution by removing participants with indications of possible OSA. We have added the following information to the Participants section of the Methods to clarify screening procedures:

- Participant recruitment began with an online survey. Potential participants were then further screened by phone call to see if they met eligibility criteria. They then underwent additional screening and an informed consent discussion in person prior to potential enrollment in the study. In general, screening consisted of questions regarding demographics, availability, medical and mental health, medications, and sleep. Participants with responses meeting exclusion criteria were not included in the study. As part of screening, for example, participants were asked about current or prior diagnoses

of Obstructive Sleep Apnea (OSA); if yes, the participant was excluded. Full eligibility criteria can be found on the ClinicalTrials.gov website. As part of the pre-intervention baseline sleep visit, sleep technicians monitored for sleep quality. During the baseline sleep visit, two participants had loud snoring, breath holding and gasping accompanied by periods of wake. Given concern for OSA and overall poor sleep that would preclude experiment completion, these two participants were withdrawn after their baseline visit (**Figure 1C**).

The choice of a 15 kHz carrier frequency is briefly noted but not fully explained. Could the authors elaborate on the rationale for this selection and whether alternative frequencies were considered?
We agree that additional context for the choice of 15 kHz carrier frequency is helpful. The rationale for 15 kHz stems from the relationship between carrier frequency and impedance. A higher carrier frequency lowers the impedance at the electrode-scalp interface such that higher currents can be used without the participant feeling sensations. It was very important that TES-TI be sensation-free for participants to not risk waking them up from sleep. In addition, during an initial piloting phase for the study, several carrier frequencies were utilized, and 15 kHz had the greatest and most consistent effect.
We have added the following information to the TES-TI section of the Methods to clarify choice of 15 kHz carrier frequency:

- TES-TI protocols during NREM sleep had the following parameters: 15,000 Hz carrier frequency, 1 Hz difference frequency, sinusoidal waveform, 5 mA peak to peak, 3-minute duration with 15-second ramping up/down periods. Channel 1 used a 15,000 Hz frequency and channel 2 used a 15,001 Hz frequency. As a higher carrier frequency lowered impedance at the electrode-scalp interface, a 15,000 Hz carrier frequency was chosen to mitigate the possibility of sensations being felt by participants during sleep.

Minor Comment

The manuscript has some inconsistencies in font styles and sizes that should be corrected for uniformity.
We have edited the manuscript to try to remove inconsistencies in font styles and sizes.

Reviewer #2 (Remarks to the Author):

This manuscript evaluates transcranial electrical stimulation with temporal interference (TES-TI) using a 1-Hz offset to modulate slow-wave activity (SWA) during NREM sleep. Sleep architecture appears unchanged, whereas TES-TI transiently increases SWA and modulates other sleep-related frequency bands beyond stimulation periods. The approach is promising as a potentially more focal, deeper-reaching alternative to existing non-invasive methods, with fewer perceptual sensations that might disturb sleep. The study is thoughtfully designed and the results are very promising with clear implications for basic and clinical research. However, several aspects of the sample size justification and statistical analysis currently limit the strength of the conclusions and require additional clarification and robustness checks.

Major comments:
1. The control group size is justified using power calculations for paired, one-sided t-tests, yet the manuscript reports between-group comparisons (Fig. 3B). Power based on a paired, one-tailed test seems not appropriate for a between-subject contrast and likely underestimates the required sample size of the TES control group. One-sided testing also presupposes a clear

directional hypothesis not specified in the preregistration (NCT06267521). I suggest to recalculate power based on the full TES-TI sample using two-sided α.

In consultation with Dr. Marshall (a statistician now added to the author list), and based on the Reviewer's comment, we fully acknowledge the issue in reporting statistical power analyses using one-sided tests when there was no preregistered directional hypothesis. We apologize for the improper usage of this analysis.

As the Reviewer suggested, we calculated power based on the observed data using the full TES-TI sample with a two-sided alpha. We removed the prior analysis. For the main findings pertaining to the TES-TI group, our analyses were highly powered (e.g., Figure 3, STIM-PRE, N=21, Cohen's d = 1.45, β = 0.99, α = 0.05, two-sided paired samples t-test). For the comparison between TES-TI and TES groups, our analysis was moderately powered (Figure 4A, B, TES^{15kHz}-TI^{1Hz} N=21, TES^{15kHz} N=7, Cohen's d=1.08, β = 0.66, α = 0.05, two-sided independent samples t-test). Although statistical power for this comparison was moderate (β = 0.66), a significant group difference was nevertheless detected with a large effect size (Cohen's d = 1.08). Reduced power primarily increases the likelihood of a Type II error (failure to detect a true effect) rather than a Type I error (false positive). The presence of a significant, large effect is not negated by the moderately powered test. Given that potential effects of high-frequency-only TES are an area of active investigation, but still rarely reported in comparison to TES-TI, we viewed this comparison as a valuable addition to our work. For these reasons, we decided to keep these results as part of the main findings. Analyses pertaining to only the TES^{15kHz} group were statistically underpowered and mostly showed no differences; therefore, Type II errors could not be reliable discounted. For these reasons, the results for these analyses have been moved to the Supplementary Information, they have been clearly qualified as preliminary (i.e., should be interpreted as exploratory and hypothesis-generating rather than confirmatory), and any previous conclusions drawn from these data have been removed from main text. In general, we have made changes to the text centered on more transparency about statistical power and more cautious phrasing about our conclusions. Below are a few examples of these numerous changes.

Figure 4C. (original Figure 3C-E.) now reports the early v. late comparison during stimulation for only the TES-TI group; the TES group results have been moved to Supplementary Figure 4. The Results section has been modified accordingly.

Added to Methods Statistics and Reproducibility

- Statistical power calculations were performed using the *pwr* functions in R (v4.5.2) for t-tests with a two-sided alpha at 0.05. The primary findings that used the observed data from participants that received the TES^{15kHz}-TI^{1Hz} intervention were highly powered (e.g., Figure 3, STIM-PRE, N=21, Cohen's d = 1.45, β = 0.99, α = 0.05, two-sided paired samples t-test). The comparison performed between TES^{15kHz}-TI^{1Hz} participants and TES^{15kHz} participants was moderately powered, but a significant difference was nevertheless detected with a large effect size (Figure 4A, B, TES^{15kHz}-TI^{1Hz} N=21, TES^{15kHz} N=7, Cohen's d=1.08, β = 0.66, α = 0.05, two-sided independent samples t-test). Preliminary findings pertaining to only the TES^{15kHz} group, given the small sample size, were included in the Supplementary Information; the results of TES^{15kHz} group analyses should be considered exploratory and hypothesis-generating rather than confirmatory. Summary statistics for the main figures, including sample sizes and effect sizes (Cohen's d), can be found in Supplementary Table 4.

Added to Results, in several places

- Analyses were also repeated for the TES^{15kHz} group, but given the small samples size, results should be considered exploratory and hypothesis-generating (**Supplementary Table 3**).

2. The correlation analyses shown in Fig. 5 raise some questions. Only changes from STIM–

PRE between first and last intervention nights are correlated with REST-Q, whereas POST–PRE effects, that were reported for the two nights separately, are omitted but seem equally or more relevant as they are free of potential stimulation-period artifacts. Please, also specify whether multiple comparison corrections were applied. A single model could be applied with REST-Q change as the dependent variable, predictors including SWA and sigma power (low/high), Window (STIM–PRE vs POST–PRE), and relevant interactions.

Thank you for raising these concerns. We agree with the Reviewer that a model including multiple predictors provides a more comprehensive approach for these analyses. We performed a regression analysis using LASSO (Least Absolute Shrinkage and Selection Operator) estimation with 5-fold cross-validation to select model hyperparameters. Specifically, we used change in power in SWA, theta, beta, low sigma, and high sigma bands during STIM-PRE and POST-PRE windows as predictors (10 independent variables) for REST-Q sleep quality ratings (dependent variable). We ran this model for first and last nights as well as the difference between last and first nights. We found that only SWA during the STIM-PRE window improved predictive performance for the difference between last and first nights in the TES-TI group. In line with our previous finding, increases in SWA during STIM-PRE on the last night relative to the first night were positively associated with increases in sleep quality ratings.

With respect to the issue of correction for multiple comparisons, the new predictive modeling approach does not use the null hypothesis testing framework and thus does not require correction for multiple comparisons. In the predictive modelling approach, training-testing split of the data via cross-validation plays the role of preventing overfitting and ensuring that results cannot be explained by random variation.

In the original text, we did not apply a correction for multiple comparisons with respect to the analyses relating SWA to changes in sleep quality ratings; we apologize for this oversight. However, for all other analyses, the Benjamini-Yekutieli procedure was used to correct families of comparisons to a false discovery rate of 5%. Associated adjusted p-values and original un-adjusted p-values can be found in Supplementary Table 4.

Lastly, we would like to reassure the Reviewer that the usage of hardware filters and procedures for thorough data review ensured that the STIM-PRE window did not contain any stimulation-period artifacts that would compromise the results.

We have added these details about the predictive modeling approach to the Methods and Results sections of the text.

We have also modified Figure 6. (original Figure 5.) and the associated Results to include SWA because it was the only predictor to improve performance and high sigma as an example of a predictor that did not improve performance. Axes were swapped to show overall REST-Q ratings on the y-axis versus change in SWA or sigma on the x-axis. The line of best fit and R^2 values were reported, where R^2 values were also plotted topographically to qualitatively assess for any regional differences in the strength of the relationship.

3. The TES-TI sample was created by pooling three subgroups (Groups 2,3,4) that received heterogenous interventions. Some TES-TI participants practiced meditation while others did not. Given known associations between meditation and sleep quality (e.g., doi: 10.1016/smrv.2015.12.001), the authors consider including a Group factor (meditation vs no meditation) as predictor and test interactions with stimulation effects. If underpowered for full interaction testing, descriptive subgroup summaries may be provided and a sensitivity analysis acknowledging the limitation.

Thank you for raising this concern and providing a reference. We have added a new section to the Methods called *Meditation Training* to provide more information about the meditation practice.

To address the appropriateness of pooling participants from different subgroups, we used a linear mixed effects (LME) model to assess for any differences across subgroups. The LME was

fit using with numerical maximum likelihood estimation using the 'lmer()' function of the 'lme4' package in R. In general, the model related the response variable (*y*) to experiment and the covariate of interest (*x*) as fixed effects with an interaction term, and participant as a random effect (that is, 'M1 = *y* ~ *x* * experiment + (1 | participant)'). We then tested the hypothesis that there was no interaction between *x* and the experiment by fitting a model with the interaction term removed (that is, 'M2 = *y* ~ *x* + experiment + (1 | participant)') and performing an asymptotic likelihood ratio test to compare the full and reduced models. In all cases, residual plots were examined for evidence against the assumptions of constant variance and normality. If no significant interaction was found, we tested for a main effect of the primary covariate by fitting a model with that term removed (that is, 'M3 = *y* ~ experiment + (1 | participant)') and performed an asymptotic likelihood ratio test to compare this model with the one including the covariate ('M2'). Specifically, for our analyses, SWA was the response variable, and TES-TI subgroup (Group 2, 3, or 4) and time period (PRE, STIM, POST) were fixed effects. Two separate models were applied, one for the first night and one for the last night. SWA was the response variable, TES^{15kHz}-TI^{1Hz} subgroup and time period (PRE, STIM, POST) were fixed effects. We found no significant interaction between subgroup and time period (First: f^2=0.16, p=0.079; Last: f^2=0.07, p=0.571) and found no main effect of subgroup (First: f^2 =0.23, p=0.482; Last: f^2=0.11, p=0.709). These results indicated that changes in SWA during and after stimulation were similar across subgroups and supported the decision to pool their data. Further supporting this approach was that a main effect of time period was found for both first and last nights (First: f^2=0.90, p=$1.42x10^{-5}$; Last: f^2=0.84, p=$2.66x10^{-6}$), in line with the analyses using aggregated subgroups. Thus, the qualitative conclusions were the same whether the subgroups were factored in with the LME or whether the subgroups were aggregated.
We have added these details to the Methods and Results.

4. To further support the authors' main claim that temporal interference is the relevant component for SWA modulation, it may help to present POST–PRE effects for the TES-only group in parallel to Fig. 2 for TES-TI as supplementary information.
We agree that it could be helpful to show the data as in Figure 3. (original Figure 2.) for TES-only. We have added Supplementary Figure 4. with this information. Changes in SWA during stimulation (STIM-PRE) were not significant for TES-only. Changes in SWA after stimulation (POST-PRE) showed two peripheral clusters of significant electrodes; however, the effect was not global as in TES-TI and did not extend to the all-channel average after correction for multiple comparisons. In the context the small sample size for the TES group, we have adjusted our language to emphasize the preliminary nature of these analyses.

5. SWA is defined as 0.5–4 Hz, whereas TES-TI targets a 1-Hz envelope difference. Specifically strong effects confined to slow oscillations (SOs; ~0.5–1.25 Hz) would be theoretically expected. Please provide analyses for SOs (0.5–1.25 Hz) alongside SW If feasible, the authors may consider an event-level SO detection and to test whether SO characteristics change from PRE to POST.
We appreciate the Reviewer's thoughtful consideration of additional analyses based on a potential mechanism of TES-TI. We would like to emphasize that the exact mechanism of TES-TI is still an area of active investigation, so it remains unclear whether a relevant mechanism may be entrainment and whether the effect should be expected to be stronger closer to the difference frequency (1 Hz in this case) as opposed to over a broader range. Regardless, appreciate the suggestion of repeating our main analyses using a narrower frequency band. We chose to use 0.5-2 Hz given variable frequency ranges reported in the literature and other Reviewer's emphasis on using frequency ranges specified in AASM guidelines. We have included the main results for 0.5-2 Hz in Supplementary Figure 3 and added them to the Results

section of the text. Results using the narrower band, 0.5-2 Hz, were highly similar to the results using the broader band, 0.5-4 Hz.
We agree with the Reviewer that the exploration of additional characteristics of slow waves via slow wave detection is an exciting avenue that could provide further insight. However, performing such analyses with respect to the effects of our TES-TI paradigm is non-trivial and beyond the scope of the current work. Nonetheless, we have started to do so and plan to present any results in future work.

Minor:
6. Fig. 3D suggests SWA increases from early to late STIM in TES-TI but not in TES. Please also test the Group × (Early vs Late STIM) interaction directly.
Given the small sample size of the TES group, we have moved these analyses to the Supplementary Information, qualified them as preliminary, and removed any previous conclusions drawn from these data from the main text. This includes the early v. late comparison for the TES group that did not detect an effect, which is now located in Supplementary Figure 4.
As the Reviewer suggested, we tested the Group x (Early vs. Late STIM) interaction directly, using a two-sided independent samples t-test to compare the difference in Late – Early STIM between TES-TI and TES. We found a small effect size and no significant difference between groups (TES-TI N=21, TES N=7, t=0.28, p=0.782, Cohen's d=0.12).
We did not include these comparisons in the updated manuscript because the relevant TES analysis was moved to the Supplement and we are not drawing conclusions from these data (see further detail in response to 1.).

7. The methods state that impedances were monitored and electrodes adjusted as needed. Please specify how adjustments were performed during sleep (criteria, timing relative to sleep stage, extent of manipulation) and whether any epochs were excluded due to adjustments.
If stimulating electrode impedances increased substantially, to greater than 6500 Ohm, then they were adjusted. Adjustments were targeted for when a participant entered a light stage of sleep (i.e., N1) or wake. Adjustments consisted of applying additional Ten20 conductive paste and/or additional measures to improve electrode adherence to the scalp (e.g., moving hair out of the way, applying small amounts of NuPrep gel). Analysis of hd-EEG data included only epochs staged as NREM 2 or 3; NREM 1 and wake epochs were excluded, so any adjustments during these time periods were also excluded. Overall, adjustments were infrequent, as out of the 49 stimulation nights analyzed, only 4 nights had adjustments, which occurred one or two times during periods of wake.
We have added the following details to the TES-TI section of the Methods.

- Electrode impedances were monitored throughout the experiment and served as an indicator for the need to adjust electrodes; substantial increases in impedance (to greater than 6500 Ohm) indicated poor positioning and a need for adjustment. Adjustments were targeted for when a participant entered a light stage of sleep (i.e., N1) or wake. Adjustments consisted of applying additional Ten20 conductive paste and/or measures to improve electrode adherence to the scalp. Overall, adjustments were infrequent, as out of the 49 stimulation nights analyzed, only 4 nights had adjustments, which occurred one or two times during periods of wake.

8. It could strengthen the mechanistic interpretation to relate stimulation-specific metrics (electrode impedances, delivered current or simulated vmPFC field strengths) to physiological effects (SWA changes STIM–PRE and POST–PRE).
We agree that these are important relationships to consider. Please see the below analyses

using the TES-TI group to compare changes in SWA (Top Row: STIM-PRE and Bottom Row: POST-PRE) to delivered current and electrode impedances as well as simulated vmPFC field strengths (mean electric field within the target regions and maximum electric field within the target regions). Spearman correlations were performed; p-values shown were unadjusted for multiple comparisons. We did not find any significant relationships for any metrics.

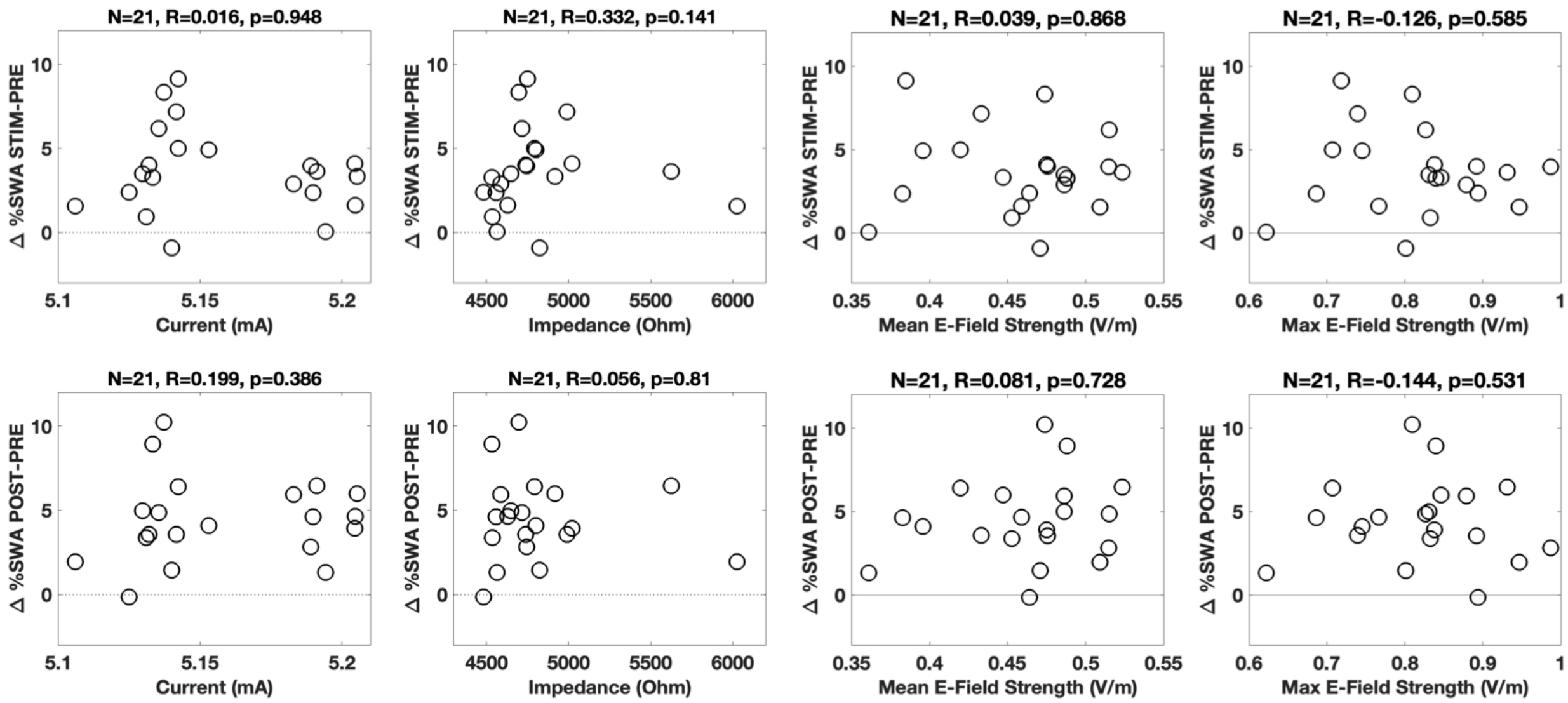

Additional studies that systematically vary the current and/or impedance to widen the possible range of values may be better able to identify potential relationships.
For simulated field strengths, while these initial analyses did not identify any relationships, we are currently working on more sophisticated approaches to evaluate potential relationships at the whole-brain level. We aim to more thoroughly investigate this question in future work.

Reviewer #3 (Remarks to the Author):

This manuscript investigates the effects of Transcranial Electrical Stimulation with Temporal Interference (TES-TI) on sleep slow wave activity (SWA). The research question is both timely and of significant interest. However, I have substantial concerns regarding the analytical methodology employed in the study. These methodological flaws are significant enough to undermine the validity of the results and the conclusions drawn. Therefore, despite the novelty of the topic, I cannot recommend this manuscript for publication in its current form and must advise rejection.
The TES15kHz-TI1Hz condition in the manuscript combines subjects from three distinct groups: (Group 2) TES15kHz-TI1Hz (1 Hz difference frequency) administered 2 nights per week with meditation; (Group 3) TES15kHz-TI1Hz administered 1 night per week with meditation; and (Group 4) TES15kHz-TI1Hz administered 2 nights per week with sham meditation. When comparing this combined condition to Group 1 (TES15kHz administered 2 nights per week with meditation), the heterogeneity within the TES15kHz-TI1Hz group is substantial, which prevents isolating the pure effects of TES-TI. To address the research question more effectively, the authors should limit the analysis to only Groups 1 and 2.
Thank you for raising this concern. To address the appropriateness of pooling participants from different subgroups, we used a linear mixed effects (LME) model to assess for any differences across subgroups. The LME was fit using with numerical maximum likelihood estimation using the 'lmer()' function of the 'lme4' package in R. In general, the model related the response variable (*y*) to experiment and the covariate of interest (*x*) as fixed effects with an interaction

term, and participant as a random effect (that is, 'M1 = *y* ~ *x* * experiment + (1 | participant)'). We then tested the hypothesis that there was no interaction between *x* and the experiment by fitting a model with the interaction term removed (that is, 'M2 = *y* ~ *x* + experiment + (1 | participant)') and performing an asymptotic likelihood ratio test to compare the full and reduced models. In all cases, residual plots were examined for evidence against the assumptions of constant variance and normality. If no significant interaction was found, we tested for a main effect of the primary covariate by fitting a model with that term removed (that is, 'M3 = y ~ experiment + (1 | participant)') and performed an asymptotic likelihood ratio test to compare this model with the one including the covariate ('M2').
Specifically, for our analyses, SWA was the response variable, and TES-TI subgroup (Group 2, 3, or 4) and time period (PRE, STIM, POST) were fixed effects. Two separate models were applied, one for the first night and one for the last night. SWA was the response variable, TES^{15kHz}-TI^{1Hz} subgroup and time period (PRE, STIM, POST) were fixed effects. We found no significant interaction between subgroup and time period (First: f^2=0.16, p=0.079; Last: f^2=0.07, p=0.571) and found no main effect of subgroup (First: f^2 =0.23, p=0.482; Last: f^2=0.11, p=0.709). These results indicated that changes in SWA during and after stimulation were similar across subgroups and supported the decision to pool their data. Further supporting this approach was that a main effect of time period was found for both first and last nights (First: f^2=0.90, p=1.42×10^{-5}; Last: f^2=0.84, p=2.66×10^{-6}), in line with the analyses using aggregated subgroups. Thus, the qualitative conclusions were the same whether the subgroups were factored in with the LME or whether the subgroups were aggregated.
We have added these details to the Methods and Results.

The interventions described for each group in the manuscript differ from those listed in the clinical trial registration. The registration specifies four groups as follows: (1) meditation practice alone; (2) meditation practice combined with high-dose TES-TI; (3) meditation practice combined with low-dose TES-TI; and (4) TES-TI alone. Please clarify these inconsistencies and explain any deviations from the registered protocol.
We apologize for the confusion created by the Group mix up as listed in the original draft of the manuscript (Groups 2 and 4 were swapped). We have corrected the text and added new Figure 1. Experiment overview to help clarify which groups received which interventions. Briefly, the correct groupings are as follows: Group 1: meditation + TES twice per week, Group 2: sham meditation + TES-TI twice per week, Group 3: meditation + TES-TI once per week, and Group 4: meditation + TES-TI twice per week.
As the Reviewer noted, the groups as listed under the Primary Objectives in the Study Overview section of ClinicalTrials.gov is not fully correct. We will work on rectifying this. However, when looking under the Study Plan, Participant Group / Arm section, the information is correct and agrees with the updated version of the manuscript with the exception being TES is referred to as "Sham Stimulation" and TES-TI is referred to as "Stimulation."

The introduction should elaborate on the importance of slow-wave activity (SWA), discussing its relationships with sleep architecture and cognitive performance. Additionally, clarify whether the primary aim is to enhance sleep quality to improve cognitive outcomes.
We have added the following information and references about SWA in relation to sleep architecture and the importance of SWA in cognitive performance, specifically memory.
The primary aim of this work was to enhance SWA using a novel neuromodulation technique. Cognitive performance was mentioned as an example of why SWA is important. Follow-up studies may be designed to specifically address relationships to cognitive outcomes.

- Sleep slow waves are homeostatically regulated. Thus, slow wave activity (SWA, electroencephalographic power in 0.5–4 Hz range), a combined measure of the number and amplitude of slow waves, is high in early sleep, even higher after sleep deprivation,

and declines in the course of sleep when sleep need is discharged [2-4]. SWA is maximal during the deepest stages of sleep. Slow waves are believed to mediate key restorative functions attributed to NREM sleep, including synaptic renormalization and memory consolidation [5-11]. Slow waves, in conjunction with spindles and sharp-wave ripples, may coordinate the reorganization of memories within the cortex [9-11]. Disrupted SWA is associated with worsened cognitive functioning, including poorer performance on visuomotor and perceptual learning tasks [12-14]. Conversely, the enhancement of SWA has been shown to improve cognitive performance in several domains, such as verbal fluency, working memory, and declarative memory [15-19].

9. Diekelmann, S., & Born, J. The memory function of sleep. *Nature reviews* **11**, 114-126 (2010).
10. Born, J. Slow-wave sleep and the consolidation of long-term memory. *World J. Biol. Psychiatry* **11**, 16-21 (2010).
17. Diep, C., *et al.* Acoustic slow wave sleep enhancement via a novel, automated device improves executive function in middle-aged men. *Sleep* **43**, zsz197 (2020).

.

As TES-TI is a relatively novel technique, the authors should provide a comprehensive yet accessible background, including its general effects, the rationale for selecting the carrier frequency, and the mechanism underlying the difference frequency.

We have added the following information and updated citations to the Introduction to expand on TES-TI and its general effects. We have also added the following details to the Introduction to clarify that the exact mechanism of TES-TI is still an area of active investigation.

- An innovative neuromodulatory tool potentially able to overcome these limitations is Transcranial Electrical Stimulation with Temporal Interference (TES-TI), also called Temporal Interference Stimulation (TIS)[22] or interferential current stimulation [23-26].
- While the exact mechanism by which TES-TI modulates neural activity is an area of active investigation [22,26,29,30], several studies have now implemented TES-TI with high frequency carriers (up to 20 kHz) in a safe and effective manner in humans [31-35]. For example, in healthy populations, reported effects include changes in connectivity [33,34,36] and/or behavior on various tasks, including motor learning, episodic memory, and spatial navigation [34-38]. Currently, less is known in clinical populations; however, preliminary evidence suggests potential benefits for individuals with Parkinson's disease or epilepsy [34,39]. To our knowledge, no study has reported on the use of TES-TI to promote sleep, and more specifically, to enhance SWA.

23. Ganne, J. M. Interferential therapy. *Aust. J. Physiother* **22**, 101-110 (1976).
24. Goats, G.C. Interferential current therapy. *Br. J. Sports Med* **24**, 87-92 (1990).
Ward, A.R. Electrical stimulation using kilohertz-frequency alternating current. *Phys. Ther* **89**, 181-190 (2009).
25. Ward, A.R. Electrical stimulation using kilohertz-frequency alternating current. *Phys. Ther* **89**, 181-190 (2009).
26. Opancar, A., *et al*. The same biophysical mechanism is involved in both temporal interference and direct kHz stimulation of peripheral nerves. *Nature Communications* **16**, 9006 (2025).
30. Caldas-Martinez, S., *et al*. Cell-specific effects of temporal interference stimulation on cortical function. *Communications biology* **7** 1076 (2024).
34. Mansourinezhad, P., *et al.* Systematic review of experimental studies in humans on transcranial temporal interference stimulation. *J Neural Eng* **22**, (2025).
35. Albantakis L. & Tononi G. Precision neuromodulation in psychiatry: focus on temporal interference stimulation. (in press at *Am. J. of Psychiatry*) (2026).
36. Kurtin, D. L., *et al*. Task-related changes in resting state connectivity are affected by temporal interference (TI) stimulation. *Brain stimulation* **18,** 937–947 (2025).

We agree that additional context for the choice of 15 kHz carrier frequency is helpful. The rationale for 15 kHz stems from the relationship between carrier frequency and impedance. A higher carrier frequency lowers the impedance at the electrode-scalp interface such that higher currents can be used without the participant feeling sensations. It was very important that TES-TI be sensation-free for participants to not risk waking them up from sleep. In addition, during an initial piloting phase for the study, several carrier frequencies were utilized, and 15 kHz had the greatest and most consistent effect.
We have added the following information to the TES-TI section of the Methods to clarify choice of 15 kHz carrier frequency:

- TES-TI protocols during NREM sleep had the following parameters: 15,000 Hz carrier frequency, 1 Hz difference frequency, sinusoidal waveform, 5 mA peak to peak, 3-minute duration with 15-second ramping up/down periods. Channel 1 used a 15,000 Hz frequency and channel 2 used a 15,001 Hz frequency. As a higher carrier frequency lowered impedance at the electrode-scalp interface, a 15,000 Hz carrier frequency was chosen to mitigate the possibility of sensations being felt by participants during sleep.

The writing lacks academic rigor in several places. For example, in the first sentence of the main text, "EEG" appears without its full name (electroencephalography) on first use. In the abstract, it states that 21 subjects received TES-TI, whereas the study includes 28 participants overall. These issues should be corrected for clarity and consistency.
We have added the full name for EEG in the first sentence and edited the manuscript to try to remove any other instances where an acronym is not pre-defined.
In the Abstract, we added the participant count for the TES group as written below to help clarify that there were N=21 TES-TI participants and N=7 TES participants for a total of 28 participants.

- Two high frequency carriers with 1Hz difference (TES^{15kHz}-TI^{1Hz}) produced amplitude-modulated temporal interference at 1Hz. Participants (N=21) received TES^{15kHz}-TI^{1Hz} during NREM sleep over repeated nights (~10 stimulation periods, 3-min each, first half of night, 1-2 nights/week, 4-week protocol), targeting left ventromedial prefrontal cortex. Seven other participants received TES^{15kHz} (15kHz carriers, 0Hz difference, 2 times/week).

We have also clarified this in the Methods and Results.

- In total, twenty-eight participants (30.5±9.9 years, 61.0% Female, N=21 TES^{15kHz}-TI^{1Hz}, N=7 TES^{15kHz}) from the first five cohorts of the STRENGTHEN study were included in the current study.

The manuscript reports multiple outcomes with varying sample sizes for comparisons, without applying corrections for multiple comparisons. This raises concerns about statistical validity and potential Type I errors. Please justify the approach or implement appropriate adjustments.
Thank you for raising this concern. The Reviewer is correct in that we did not originally apply a correction for multiple comparisons with respect to the analyses relating SWA to changes in sleep quality ratings. We apologize for this oversight. We have corrected this issue, and on the advice of another Reviewer, adopted a predictive modeling approach to these analyses that does not require correction for multiple comparisons; rather, training-testing split of the data via cross-validation plays the role of preventing overfitting and ensuring that results cannot be explained by random variation.
For all other analyses, the Benjamini-Yekutieli procedure was used to correct families of comparisons to a false discovery rate of 5% (*fdr_bh* in MATLAB). P-values reported in the text are adjusted according to this procedure; both original, unadjusted p-values and the reported adjusted p-values can be found in Supplementary Table 4.

Provide a clear timeline indicating when baseline assessments were conducted relative to the interventions and follow-ups.
In line with another Reviewer comments as well, we aimed to clarify the study timeline in a new figure. Figure 1. STRENGTHEN study overview now provides a depiction of when assessments, interventions, and follow-ups occurred. Additional details have been added to the Study Design section of the Methods.

Different outcomes are analyzed using different sample sizes. Please explicitly detail these variations, including the number of participants from each of the four groups for every outcome measure.
Thank you for raising this concern; we agree that which participants were included in which analyses could be a point of confusion for readers. We have added new Supplementary Figure 1. to provide details about which participants went into which analysis.

The authors mention that data were derived from five cohorts, but the meaning of "cohorts" is unclear. Please define and explain what these cohorts represent, how they were recruited, and their relevance to the study design.
We agree that the meaning of cohorts was ambiguous as written. A cohort is simply a set of 5-6 participants that, for logistical reasons (availability of rooms, devices, and personnel) went through the various phases of the study as a group. We have added a sentence to clarify this point.

- The current study reflects a mechanistic sub-study within the broader STRENGTHEN trial; it is an interim analysis that focused on the effects of TES^{15kHz}-TI^{1Hz} during NREM sleep using data collected from the first five cohorts ~~(out of nine)~~ of the STRENGTHEN trial (from February 19, 2024, to December 31, 2024). Each cohort contained approximately 5-6 participants who, for logistical reasons, went through the various phases of the study as a group.

Reviewer #4 (Remarks to the Author):

In this high-density EEG (hd-EEG) sleep study, Schaeffer and colleagues investigated the effects of TES15 kHz-TI1Hz on sleep slow wave activity (SWA) in healthy participants, comparing their responses to those of healthy control subjects who underwent TES alone. The TES-TI group showed a significant increase in both sleep slow wave and theta activity, whereas the TES-only group did not. The authors suggest that TES15 kHz-TI1Hz can enhance sleep SWA and theta activity, while TES alone does not.

General comments:
The manuscript has many strengths. The study is well-motivated and theoretically sound, and the combination of hd-EEG with transcranial electric stimulation is highly sophisticated. The findings are interesting, novel, and contribute meaningfully to the existing literature.

I have some comments and suggestions, most of which are minor or moderate:

Introduction:
The introduction provides a strong theoretical background. No specific comments.

Sleep scoring:
1. Awakenings: According to AASM (2023), arousals should be reported as both the number of arousals and as the arousal index (number of arousals × 60/TST). It is unclear what is meant by

"Number of awakenings" in Tables 2, 3, and Supplementary Table 1. Please use AASM-recommended terminology.

Thank you for raising this concern. We agree with the Reviewer that arousals are an important parameter to consider. We calculated both the number of arousals and the arousal index (number of arousals per hour) for each night. The average arousal index calculated across all nights (9.08 [7.9-10.2]) was in line with the values reported for typical polysomnography recordings, according to the below reference provided by the Reviewer (9.6 [8.8-10.5] for 18-34 year olds; 12.5 [10.7-14.2]). Number of arousals and arousal index values have been added to Tables 2, 3, and Supplementary Table 1. No statistically significant changes were found across baseline, first, and last nights for TES-TI or TES participants.

Number of awakenings refers to the number of times the stage transitioned from sleep (NREM 1,2,3 or REM) to wake. We believe the parameter helps indicate whether participants experienced more frequent wake episodes under the various conditions. No significant differences in the number of awakenings across baseline, first, and last nights for TES-TI or TES, contributed to our evidence that neither TES-TI nor TES interventions resulted in more frequent wake episodes, thereby having a deleterious effect on objective sleep quality. The addition of arousal metrics also supports to this conclusion, and so we thank the Reviewer for this contribution.

We have clarified the distinction between number of awakenings and new arousal metrics in the Methods.

- CountingSheepPSG (v1.4.5) MATLAB software was then used to perform staging in 30-second epochs and calculate sleep metrics such as Total Sleep Time, Sleep Onset Latency (to Stage 1), Wake After Sleep Onset, and others. Number of arousals and arousal index (number of arousals per hour) were quantified in line with AASM guidelines [54]. Number of awakenings was also included and refers to the number of times the stage transitioned from sleep to wake.

We have added the following lines to the Results and Discussion to incorporate the finding pertaining to arousals.

- Notably, there are no differences in metrics related to wake, including the number of arousals, arousal index, number of awakenings, or time spent awake after sleep onset.
- Indeed, when comparing general sleep architecture metrics, which were comparable to [65], across baseline, first and last intervention nights, we found that TES^{15kHz}-TI^{1Hz} did not affect the sleep architecture and, more specifically, did not increase the number of arousals, arousal index, number of awakenings, or the time spent awake after sleep onset (Table 3).

2. Sleep stage percentages: The percentages of sleep stages in Tables 2, 3, and Supplementary Table 1 deviate slightly from typical values reported in the literature (see e.g., Boulos et al., 2019, The Lancet Respiratory Medicine, 7(6), 533–3. https://doi.org/10.1016/S2213-2600(19)30057-8) and from clinical experience. The N1 stage appears reduced, and N2 increased, compared to typical values; N3 is on the lower side. Additionally, the number of awakenings (if this refers to arousals) is relatively high in Tables 2 and 3. These atypicalities raise questions about the scoring and suggest a need to clarify the quality of sleep scoring and the effects of TES-TI/TES on sleep architecture. Do the authors have an explanation for these discrepancies?

Thank you for your inspection of our sleep staging values and the cited reference. We agree that the mean and standard deviation for percentage of N1, N2, and N3 reported in our manuscript differs from the values obtained via polysomnography reported in the cited reference. The cited reference did report very high heterogeneity across studies (I^2 for sleep metrics ranging 81.6% to 98.3%), indicating high variability in the sleep metrics reported for each study.

We did find examples in which the values reported in our work are within the range of what can be found in healthy adults less than 50 years old. The cited reference shows mean N1 percentage versus age (Figure 3D) in which many studies report N1 percentages below 5%, and as low as ~1% in healthy adults less than 50 years old. N2 percentage and N3 percentage versus age were not reported in the cited reference but we found estimates in an older meta-analysis that suggest N2 percentages can be as high as ~65% and N3 percentages as low as ~5% in healthy adults less than 50 years old [a].
Although our values were in line with at least some studies reported in the literature, we sought to better understand why they did not align with most studies. We verified that our data was staged accurately, according to AASM guidelines, and confirmed that percentage calculations were computed correctly in relation to total sleep time. We reviewed the distributions for each metric and found no evidence of statistical outliers that could impact the reported values.
Given no issues found with the data, one possible explanation for the discrepancy may be the difference in data collection techniques used in the cited reference versus in our work. In the cited reference, values appear to be obtained from studies that used full, diagnostic polysomnography (PSG), including EEG (4-6 channels), EOG, Chin EMG, ECG, respiratory sensors / belts, SpO2 sensors, and limb EMG. In our study, we used high-density EEG (256-channels) and abridged polysomnography, including EOG, Chin EMG, and ECG because a full diagnostic PSG was found to not be necessary for the goals of the study.
We found several hd-EEG studies that reported percentages of various stages [65,b,c]. In [65], 256-channels were used and the sample population was comparable to ours ([b] was an all-male sample and [c] included adolescents). The study in [65] also used an abridged polysomnography like ours. The values reported in this study for the baseline night and those reported in our work showed overlapping distributions within one standard deviation, suggesting that the values were broadly comparable with overlapping variability. Specifically, N1 percentage in their work is listed as 4.3±2.6 and in ours averages to 4.0±3.1; N2 percentages were 52±5.9 in theirs and 61.2±7.0 in ours; N3 percentages were 21±5.8 in theirs and 13.6±6.2 in ours.
Given the same sleep staging approach used consistently across all nights in the study, we believe that the values reported for TES-TI and TES groups across the various nights are internally consistent; therefore, the findings that there were no changes in sleep staging metrics across nights cannot be explained by inconsistent staging.
We have added the following information to the Discussion to briefly contextualize the values we report as comparable to the hd-EEG study in [65].

- Indeed, when comparing general sleep architecture metrics, which were comparable to [65], across baseline, first and last intervention nights, we found that TES^{15kHz}-TI^{1Hz} did not affect the sleep architecture…

65. Stephan, A. M., *et al*. Conscious experiences and high-density EEG patterns predicting subjective sleep depth. *Current biology* **31**, 5487–5500 (2021).
a. Ohayon MM, Carskadon MA, Guilleminault C, Vitiello MV. Meta-analysis of quantitative sleep parameters from childhood to old age in healthy individuals: developing normative sleep values across the human lifespan. Sleep 2004; 27: 1255–73.
b. Riedner, B. A., Vyazovskiy, V. V., Huber, R., Massimini, M., Esser, S., Murphy, M., & Tononi, G. (2007). Sleep homeostasis and cortical synchronization: III. A high-density EEG study of sleep slow waves in humans. *Sleep*, *30*(12), 1643–1657. https://doi.org/10.1093/sleep/30.12.1643
c. Mayeli, A., Janssen, S. A., Sharma, K., & Ferrarelli, F. (2022). Examining First Night Effect on Sleep Parameters with hd-EEG in Healthy Individuals. *Brain sciences*, *12*(2), 233. https://doi.org/10.3390/brainsci12020233

3. REM latency: REM latency is missing from Tables 2, 3, and Supplementary Table 1. If possible, please include this parameter, as it would be interesting for readers to see whether the stimulations (which partially occurred during the first NREM phase) delayed or advanced the onset of REM sleep.
We agree that REM latency is an important parameter that was not initially included. REM Latency has been added to Tables 2, 3, and Supplementary Table 1. No statistically significant changes were found across baseline, first, and last nights.

4. Slow wave activity and arousals: How did the authors ensure that slow wave activity related to possible hypnopompic hypersynchrony (caused by brief arousals related to stimulation) did not confound the results?
Thank you for raising the issue of hypnopompic hypersynchrony. We did not initially consider this possibility and so we further investigated the concept in relation to our data. Hypnopompic hypersynchrony is characterized by rhythmic delta or theta waveforms, more prominent in fronto-central regions, with no evolution during sleep-wake transitions (https://onlinelibrary.wiley.com/doi/full/10.1002/epd2.20040).
We compared the example EEG traces of hypnopompic hypersynchrony with examples of our EEG data during and after stimulation. We identified a few key differences.
First, in our data, on video, participants did not show evidence of wake during or after stimulation, whereas, in the hypnopompic hypersynchrony examples, patients showed evidence of wake on video. Along these lines, technicians would have staged epochs during and/or after stimulation as wake, and this was not the case for most stimulation protocols.
Second, in our data, EEG traces showed heterogeneity (various slow waves, spindles) during and after stimulation, whereas, in the hypnopompic hypersynchrony examples, there was no evolution of the EEG data (i.e., there was a lot of homogeneous delta). Below we have provided an example of EEG data during hypnopompic hypersynchrony on the left (A) and our EEG data for two separate stimulation protocols start (B1, C1) and end (B2, C2). EEG data show 20 frontal channels filtered 0.5-40 Hz, 30-second epoch x-scale, 150 µV y-scale. Stimulation start markers are in green and stimulation end markers are in dark blue.
Finally, the increases in SWA during and after stimulation we identified were global, unlike the fronto-central prominence characteristic of hypnopompic hypersynchrony.
Although this was not a fully comprehensive evaluation, the example stimulations we reviewed provided evidence in favor of changes in genuine SWA opposed to hypnopompic hypersynchrony.

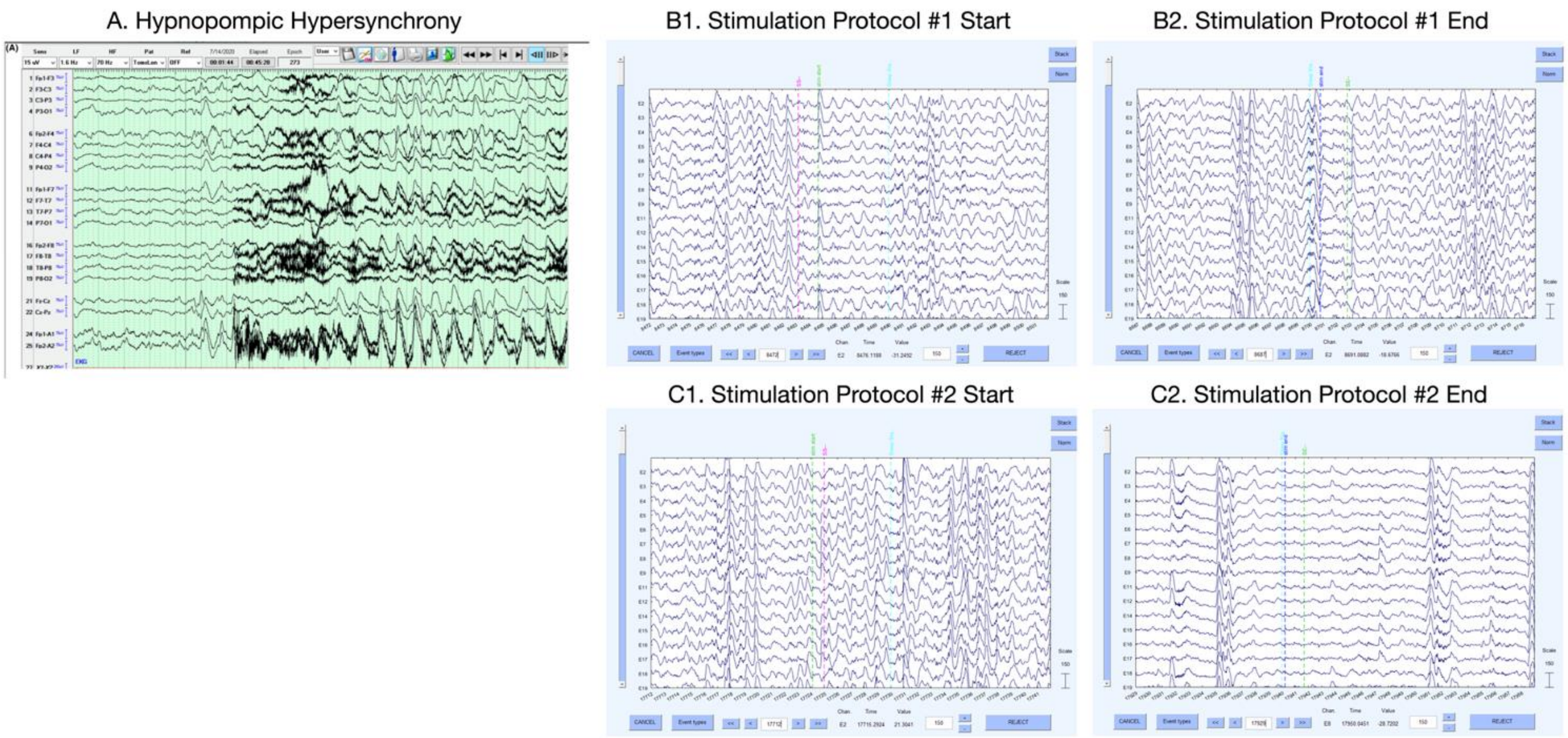

Figure 1 / Experimental overview / Methods section:
The study presents data from baseline, first, and last intervention nights, with the first and last nights separated by a 4-week intervention protocol.

1. It would be helpful for readers if the durations of the recording (in minutes) for the pre-, during-, and post-stimulation periods were indicated in Figure 1.

There are small horizontal lines to indicate time for the durations of pre-, during-, and post-stimulation periods in Panel D of Figure 2. (original Figure 1.) “1 m” is meant to represent 1-minute of time and “1 s” in the inset is meant to represent 1-second of time, where the inset is a snapshot of data from the greater 3-minute period. We tried several iterations of this figure and found a 3-minute marker to be too crowded. We have increased the font size for these markers and ensured that 3-minute periods are clarified in the figure legend.

2. What does the meditation intervention for 3 out of 4 groups signify, and could it have affected the results?

Thank you for raising this concern; we agree that information regarding the meditation intervention was lacking in the text. We have added the below information to a new section of the Methods called *Daytime Meditation Training*.

- Meditation and sham meditation interventions were delivered via the Healthy Minds Program (HMP) smartphone app, which provides a comprehensive curriculum for well-being training based upon four pillars of well-being: awareness, connection, insight, and purpose [42,43]. The program includes didactic mini podcasts that provide the background science underlying each of the four well-being pillars, along with specific mental exercises–forms of procedural learning–that complement the declarative scientific content [42,43]. Participants assigned to the meditation intervention were guided through brief, daily meditation practice (5-30 minutes) and provided regular reminders of key practice goals several times each day. Each week focused on one of the four pillars of well-being. Participants assigned to the sham meditation intervention listened to didactic material only, without meditation instruction.

42. Davidson, R. J. Mindfulness and more: toward a science of human flourishing. *Psychosom Med* **83**, 665-668 (2021).
43. Hirshberg M., *et al*. A randomized controlled trial of a smartphone-based well-being training in public school system employees during the COVID-19 pandemic. *J Educ Psychol*. **114**, 1895-1911 (2022).
To address if meditation could have affected the results, and to address other Reviewer concerns about the appropriateness of pooling participants from different subgroups, we used a linear mixed effects (LME) model to assess for any differences across subgroups. SWA was the response variable, and TES-TI subgroup (Group 2, 3, or 4) and time period (PRE, STIM, POST) were fixed effects. Two separate models were applied, one for the first night and one for the last night. SWA was the response variable, TES^{15kHz}-TI^{1Hz} subgroup and time period (PRE, STIM, POST) were fixed effects. We found no significant interaction between subgroup and time period (First: f^2=0.16, p=0.079; Last: f^2=0.07, p=0.571) and find no main effect of subgroup (First: f^2 =0.23, p=0.482; Last: f^2=0.11, p=0.709). These results indicated that changes in SWA during and after stimulation were similar across subgroups and supported the decision to pool their data. We have added these details to the Methods and Results.

Results / Number of subjects:
There was no main effect of night (baseline, first, or last) for SWA or for the first four hours in either group. Instead, SWA increased during and after TES-TI, but not in the TES-only condition. The TES-only group included 7 subjects (5 completed all three nights). While the statistical results for the TES-TI group are convincing, the authors should present calculations to justify the low number of subjects in the TES-only group, to assure readers that the sample size is sufficient to exclude false negative results.
In consultation with Dr. Marshall (a statistician now added to the author list), and based on the Reviewer's comment, we fully acknowledge the issue in reporting statistical power analyses using one-sided tests when there was no preregistered directional hypothesis. We apologize for the improper usage of this analysis.
As the Reviewer suggested, we calculated power based on the observed data using the full TES-TI sample with a two-sided alpha. We removed the prior analysis. For the main findings pertaining to the TES-TI group, our analyses were highly powered (e.g., Figure 3, STIM-PRE, N=21, Cohen's d = 1.45, β = 0.99, α = 0.05, two-sided paired samples t-test). For the comparison between TES-TI and TES groups, our analysis was moderately powered (Figure 4A, B, TES^{15kHz}-TI^{1Hz} N=21, TES^{15kHz} N=7, Cohen's d=1.08, β = 0.66, α = 0.05, two-sided independent samples t-test). Although statistical power for this comparison was moderate (β = 0.66), a significant group difference was nevertheless detected with a large effect size (Cohen's d = 1.08). Reduced power primarily increases the likelihood of a Type II error (failure to detect a true effect) rather than a Type I error (false positive). The presence of a significant, large effect is not negated by the moderately powered test. Given that potential effects of high-frequency-only TES are an area of active investigation, but still rarely reported in comparison to TES-TI, we viewed this comparison as a valuable addition to our work. For these reasons, we decided to keep these results as part of the main findings. Analyses pertaining to only the TES^{15kHz} group were statistically underpowered and mostly showed no differences; therefore, Type II errors could not be reliable discounted. For these reasons, the results for these analyses have been moved to the Supplementary Information, they have been clearly qualified as preliminary (i.e., should be interpreted as exploratory and hypothesis-generating rather than confirmatory), and any previous conclusions drawn from these data have been removed from main text. In general, we have made changes to the text centered on more transparency about statistical power and more cautious phrasing about our conclusions. Below are a few examples of these numerous changes.

Figure 4C. (original Figure 3C-E.) now reports the early v. late comparison during stimulation for only the TES-TI group; the TES group results have been moved to Supplementary Figure 4. The Results section has been modified accordingly.
Added to Methods Statistics and Reproducibility

- Statistical power calculations were performed using the *pwr* functions in R (v4.5.2) for t-tests with a two-sided alpha at 0.05. The primary findings that used the observed data from participants that received the TES^{15kHz}-TI^{1Hz} intervention were highly powered (e.g., Figure 3, STIM-PRE, N=21, Cohen's d = 1.45, β = 0.99, α = 0.05, two-sided paired samples t-test). The comparison performed between TES^{15kHz}-TI^{1Hz} participants and TES^{15kHz} participants was moderately powered, but a significant difference was nevertheless detected with a large effect size (Figure 4A, B, TES^{15kHz}-TI^{1Hz} N=21, TES^{15kHz} N=7, Cohen's d=1.08, β = 0.66, α = 0.05, two-sided independent samples t-test). Preliminary findings pertaining to only the TES^{15kHz} group, given the small sample size, were included in the Supplementary Information; the results of TES^{15kHz} group analyses should be considered exploratory and hypothesis-generating rather than confirmatory. Summary statistics for the main figures, including sample sizes and effect sizes (Cohen's d), can be found in Supplementary Table 4.

Added to Results, in several places

- Analyses were also repeated for the TES^{15kHz} group, but given the small samples size, results should be considered exploratory and hypothesis-generating (**Supplementary Table 3**).

Discussion:
The authors interpret the increase in theta power similarly to the increase in SW However, theta power is typical of N1 sleep, and this finding, together with changes in sigma power (i.e., sleep spindles, typical for N2 sleep), could also suggest lighter sleep (N2 → N1). Did the authors consider or wish to discuss the possibility that increases in SWA (deep sleep) were accompanied with some findings that might reflect lightening of sleep?
Thank you for raising this point. We agree with the Reviewer that theta power can be characteristic of N1 (lighter) sleep. Note we did not include any N1 epochs in our spectral power analyses and so any changes in theta power reported were from epochs staged as N2 or N3. As described above, we found no evidence of increased arousals, awakenings, or time spend awake that might indicate a lightening of sleep. Instead, the increase in theta activity appears continuous with the increase in delta activity, thereby suggesting an overall deepening of sleep. At the cellular level, this is consistent with our work showing that, during NREM sleep, alternations of ON/OFF periods spanning the delta range are locked to increases in theta power [63,a]. In the context of an increase of delta, then, a concomitant increase in theta is to be expected. On the other hand, as rightly pointed out, an increase in theta accompanied by a decrease in delta would indicate a lightening of sleep. We also note that, in the original two process model, sleep pressure builds with time spent awake and dissipates with time spent asleep; not only does SWA show this pattern but so too does theta power.
The finding of a decrease in sigma power is in line with deepening of sleep, specifically the transition from N2 to N3, in which there is lower sigma power, spindle density, and spindle frequency as well as higher SWA. As identified from human intracranial EEG recordings, these dynamics are especially evident in earlier NREM sleep [79-81], which is when most of our stimulation protocols were performed. While at the level of the thalamus, SWA and sigma power have been shown to follow a parallel time course, at the cortical level, as has been reported in many EEG studies, the two frequency bands demonstrate an inverse relationship within and across NREM episodes [79-81].
We have added the following sentences to the Discussion and provided additional references.

- Sleep onset is typically accompanied by an increase in both delta and theta power [70,71] and recovery sleep is characterized by increases in low frequency power that extend into the theta band [72-75]. An increase in theta activity continuous with an increase in SWA is an expected consequence of increased sleep pressure [2,3,72-75].
  63. Findlay, G., *et al*. A hippocampal 'sharp-wave sleep' state that is dissociable from cortical sleep. *Nature neuroscience* **29,** 399-410 (2026).
  73. Aeschbach, D., *et al*. Dynamics of the human EEG during prolonged wakefulness: evidence for frequency-specific circadian and homeostatic influences. *Neuroscience letters*, **239**, 121–124 (1997).
  74. Finelli, L. A., *et al*. Dual electroencephalogram markers of human sleep homeostasis: correlation between theta activity in waking and slow-wave activity in sleep. *Neuroscience*, **101**, 523–529 (2000).
  75. Cajochen, C., *et al*. Frontal predominance of a relative increase in sleep delta and theta EEG activity after sleep loss in humans. *Sleep research online : SRO* **2**, 65–69 (1999).
  a. Driessen, K., Squarcio, F., Tononi, G., & Cirelli, C. (2025). Induction of cortical ON/OFF periods in awake mice fulfills sleep functions. *bioRxiv : the preprint server for biology*, 2025.10.04.680459. https://doi.org/10.1101/2025.10.04.680459
- The decrease in power in the sigma range (12–16 Hz) after TES^{15kHz}-TI^{1Hz} (and a trend during TES^{15kHz}-TI^{1Hz}) may also reflect a deepening of sleep. In general, a reduction in spindles is seen with the transition to slow wave sleep (Stage N3), in recovery sleep after total sleep deprivation [72,75] and, in particular, fast spindle power has dynamics that run in the opposite direction of those of SWA [77-79]. In addition, as supported by intracranial EEG in humans, maximal SWA accompanied by decreases in spindle density and frequency are most evident in earlier NREM sleep, consistent with when most stimulation protocols were performed in this study [80,81].
  79. Uchida, S., *et al.* Sigma (12-15 Hz) and delta (0.3-3 Hz) EEG oscillate reciprocally within NREM sleep. *Brain research bulletin* **27,** 93–96 (1991).
  80. Andrillon, T., *et al*. Sleep spindles in humans: insights from intrancranial EEG and unit recordings. *J. Neurosci.* **31**, 17821-17834 (2011).
  81. Sarasso, S., *et al*. Thalamic and neocortical differences in the relationship between the time course of delta and sigma power during NREM sleep in humans. *Journal of sleep research* **30**, e13166 (2021).

Reviewer #5 (Remarks to the Author):

Authors present a study that aims to improve slow wave activity (SWA) by using Transcranial Electrical Stimulation with Temporal Interference (TES-TI, 15 kHz, 1 Hz difference). They performed overnight studies in laboratory, and recorded high density electroencephalography (EEG) to measure SWA and other frequency bands. Findings reveal that SWA increase in left ventromedial prefrontal cortex during TES-TI stimulation period and 3-minutes after, compared to baseline and traditional TES. Largest SWA effect correlated to sleep improvement (questionnaires).

Overall, the study addresses an important question, if SWA could be promoted by using novel technology. Although TES has longer history, TES-TI provides new insights for the field, as authors repurpose it to promote SW The study was interesting indeed, and I read it with high curiosity. Somewhat I lost the red line during the reading, and did not follow, especially when thinking about the actual protocol and the reasons why it was chosen. Also high amount of numbers and p-values in the text were not convenient for reader. In general, I liked the figures a

lot.

Key problems concern the presenting of the methods (how many laboratory nights, final subjects in the analysis, EEG processing, analysis, amount of data, starting and ending the recording, was there technician for the whole night following the data quality). During result and discussion parts of the text I had problems to follow, but also in the Methods chapter more details should be added.
Please, see my detailed comments belo

Abstract:
Please, define how many nights instead of "several nights".
We agree this important detail was lacking and have updated the text as follows.

- Participants (N=21) received TES^{15kHz}-TI^{1Hz} during NREM sleep over repeated nights (~10 stimulation periods, 3-min each, first half of night, 1-2 nights/week, 4-week protocol), targeting left ventromedial prefrontal cortex.

Introduction:
Authors could refer to novel suggestions that SWA enhancement could promote clearance of the brain. See review Hattem et al. 2025 doi: 10.1152/physiol.00019.2024
This would provide a direct link, that SWA improvement (also using TES-TI) could in future be tested as a therapy for prevention of Alzheimer's or Parkinson's disease.
Thank you for making this connection and providing the reference cited. We agree that the potential relationship between SWA enhancement and clearance has exciting implications for future therapies. However, it is still unclear whether enhanced clearance is favored by slow wave sleep as such, or rather, by micro-arousals and/or sleep-wake transitions. Given the complexity and importance of the topic, we felt like a rather detailed discussion would be necessary and that this was ultimately out of scope for the current work.

Authors could open that TES-TI is the same as TIS (the term used in some studies) to make the terminology clear.
We agree that it is important to clarify that TES-TI has been referred to with several names in the literature. We have added this information to the Introduction and included the associated references.

- An innovative neuromodulatory tool potentially able to overcome these limitations is Transcranial Electrical Stimulation with Temporal Interference (TES-TI), also called Temporal Interference Stimulation (TIS)[22] or interferential current stimulation [23-26].
  23. Ganne, J. M. Interferential therapy. *Aust. J. Physiother* **22**, 101-110 (1976).
  24. Goats, G.C. Interferential current therapy. *Br. J. Sports Med* **24**, 87-92 (1990).
  Ward, A.R. Electrical stimulation using kilohertz-frequency alternating current. *Phys. Ther* **89**, 181-190 (2009).
  25. Ward, A.R. Electrical stimulation using kilohertz-frequency alternating current. *Phys. Ther* **89**, 181-190 (2009).
  26. Opancar, A., *et al*. The same biophysical mechanism is involved in both temporal interference and direct kHz stimulation of peripheral nerves. *Nature Communications* **16**, 9006 (2025).

Results page 3:
Before figure 1, there should be a figure where a reader can clearly see the difference between groups, amount of laboratory nights and the full protocol in larger perspective than only one stimulation night as now presented in figure 1. Now it is really difficult to follow and try to understand the full picture of the study.

Thank you for raising this concern. We have added a new Figure 1. STRENGTHEN study overview that we believe addresses these points and helps clarify the study design.

61% Female: Would provide this information as actual amount how many.
The actual amount has been added as follows.

- Twenty-eight participants (30.5±9.9 years of age, 61% (N=17) Female)

Why did you choose the protocols for groups 1-4? Why did you end up with the subject amounts of 21 and only 7 in TES group?
The protocols for groups 1-4 were chosen as part of the experimental design for the larger STRENGTHEN clinical trial, which was aimed at improving overall well-being via enhancing cognitive flexibility and emotion regulation using two types of interventions: daytime meditation and nighttime brain stimulation. The groups were designed to control for the type and frequency of intervention.
For our analyses, N=21 in the TES-TI group came from pooling all the participants that received the TES-TI intervention (Groups 2,3,4) and N=7 in the TES group came from pooling all the participants that received the TES intervention (Group 1). We have added Supplementary Figure 1. that we believe helps to clarify how groupings were done in our analyses.

Page 4:
Please, explain the reason for the following and the reason why for some subjects it was not like that. “in most of the participants, stimulation were completed within the first four hours from sleep onset” As naturally there is more SWA during the first half of the night, this makes sense, to boost it but now it is not clear in the text, if this is the reason.
The Reviewer is right that the rationale for performing most all the stimulations in the first four hours from sleep onset was because of typically high endogenous SWA during this timeframe, even though the exact mechanism of TES-TI is still an area of ongoing investigation. We have added this point in the text:

- For most nights, stimulations were completed within the first four hours from sleep onset, as endogenous SWA was typically high during this timeframe.

On some nights, there were periods of wake during the first four hours after sleep onset such that not all ten NREM stimulations were completed in this timeframe.

I still don’t get what happened between first and last nights: how many nights there actually were and why?
What happened between the first and last nights depended on Group. During the day, every day, participants in Groups 1,3,4 performed brief, daily meditation trainings, and participants in Group 2 listened to didactic material with no meditation instruction. At night, either once (Group 3) or twice (Groups 1,2,4) a week, participants slept in the laboratory and received either TES stimulation (Group 1) or TES-TI stimulation (Groups 2,3,4). For Groups 1,2,4 there were 8 intervention nights and for Group 3 there were 4 intervention nights. As the intervention effects were hypothesized to be mediated, at least in part, by neuroplasticity, it was important to have several repeated sessions over the course of 4-weeks. We have added these details in new Figure 1. STRENGTHEN study overview and to a Daytime Meditation Training section of the Methods; we believe they will help clarify these questions.

Fig1. Very nice!
Page 5:
It is hard to follow how many datasets were included in the what analysis and why.

Thank you for raising this concern; we agree that which participants were included in which analyses could be a point of confusion for readers. We have added new Supplementary Figure 1. to provide details about which participants went into which analysis and why.

Really hard to read statistical analyses and their results in the text.
I would use TES-TI and TES for abbreviations, to be more readable. As the frequencies are presented, and are always the same, this would be fine.
We have moved some of the statistics from the text and instead placed them into tables. Specifically, values for whole-night SWA and SWA during the first four hours of sleep have been moved to Table 2, Table 3, and Supplementary Table 1. Statistics for additional frequency bands for the TES group have been placed in new Supplementary Table 3.
With respect to the other statistics, we believe it is important to report sample size, test statistic, p-values, effect sizes, and test type for each result in the text. We also have Supplementary Table 4 that perhaps more clearly summarizes statistical results.
We acknowledge that the format of the text may not be entirely reader-friendly; however, we believe that they provide valuable information to the reader as written. Similarly, other readers have communicated to us that referring to TES-TI and TES with the exact parameters used in this experiment was a welcome and informative convention.

Page 7:
You say “SWA was normalized”. Please, open in the text what you mean e.g. total SWA power/total of all frequency powers?
The hd-EEG Spectral Power Analyses section of the Methods provides details about how SWA was normalized, in line with Krugliakova et al., and as described below.

- Band power was normalized to account for differences between participants and night-to-night variability within participants [59]. For each participant and each night, the band power of interest (e.g., SWA) for each channel in PRE, STIM, and POST periods was divided by the average band power (e.g., SWA) during NREM sleep throughout the entire night to obtain a relative change (shown as a percent) from the mean overnight band power [59].
  56. Krugliakova, E., *et al.* Boosting Recovery During Sleep by Means of Auditory Stimulation. *Front Neurosci* **16**, 755958 (2022).

“Overall, we found a significant difference in the average SWA” please, be clear with these, average about what in SWA?
In this quoted instance, we mean average across channels. We have added this detail in the text as follows.

- Overall, we found a significant difference in the all-channel average SWA across PRE, STIM, and POST periods (N=21, df=2, F=30.97, p=7.48x10-9, one-way repeated measures ANOVA).

Also did you looked at the whole data, or cut the data? I realise that you got a lot of data in this study, but I think in the text, it should be more clear what part of the data you took, what channels you used, did you look at the same parts of the data for each subjects and so on, basically how did end up e.g. with the figures and the numbers that you got). As you were measuring the EEG for the whole nights, and for several nights, it was difficult to understand from which parts the actual figures were created.
Thank you for raising these points for clarification.
We used the baseline, first, and last intervention nights for the first approximate half of participants (N=28) collected as part of the STRENGTHEN study. Details in Datasets Included in the Methods, new Figure 1. STRENGTHEN study overview., and new Supplementary Figure

1. provide further information about which datasets from which participants were included in which analyses. We believe these new figures address most of the Reviewer's concerns.
We used the whole night of data for these nights except for spectral power analyses, in which we only used NREM epochs staged 2 or 3. The same procedures were employed for all subjects with datasets that were included.
We recorded from 256 high-density EEG channels using nets from MagStim Electrical Geodesics, Inc. To increase the signal-to-noise ratio, for analysis, we only used 185 scalp channels (excluded neck and face channels). We then removed any channels that had artifact and finally used interpolation for these channels to end up with a final count of 185 channels. Further details can be found in the hd-EEG Artifact Rejection section of the Methods.

Discussion:
Overall, I liked discussion a lot. In the first chapter of discussion, please refer to baseline, first night and last night, and wider look to the protocol. It should be clear that there were many laboratory nights per subject.
Thank you for this feedback. We have added a sentence with these details to the opening of the Discussion.

- We performed simultaneous hd-EEG recording and Transcranial Electrical Stimulation with Temporal Interference (TES-TI, 15 kHz, 1 Hz difference) during overnight sleep in a laboratory setting in healthy humans with the goal of enhancing SWA during NREM sleep. Baseline, first, and last nights over the course of the 4-week STRENGTHEN study protocol were included in this interim report. The results show that TES15kHz-TI1Hz targeting left ventromedial prefrontal cortical regions increased SWA during the stimulation period, and the effect outlasted the stimulation.

Study design page 16:
You mention here "daily meditation training" but I cannot see any other mention during the text about meditation. Please, provide detailed information about meditation training in this study.
Thank you for raising this concern; we agree that information regarding the meditation intervention was lacking in the text. We have added the below information to a new section of the Methods called *Daytime Meditation Training*.

- Meditation and sham meditation interventions were delivered via the Healthy Minds Program (HMP) smartphone app, which provides a comprehensive curriculum for well-being training based upon four pillars of well-being: awareness, connection, insight, and purpose [42,43]. The program includes didactic mini podcasts that provide the background science underlying each of the four well-being pillars, along with specific mental exercises–forms of procedural learning–that complement the declarative scientific content [42,43]. Participants assigned to the meditation intervention were guided through brief, daily meditation practice (5-30 minutes) and provided regular reminders of key practice goals several times each day. Each week focused on one of the four pillars of well-being. Participants assigned to the sham meditation intervention listened to didactic material only, without meditation instruction.

42. Davidson, R. J. Mindfulness and more: toward a science of human flourishing. *Psychosom Med* **83**, 665-668 (2021).
43. Hirshberg M., *et al*. A randomized controlled trial of a smartphone-based well-being training in public school system employees during the COVID-19 pandemic. *J Educ Psychol*. **114**, 1895-1911 (2022).

Participants page 16:

“Participants were excluded if they had any neuroradiologist-identified structural abnormalities of the brain.” Did you exclude any? You say 28 participants were included, but did you exclude some before ending up to this amount?
No participants were excluded based on neuroradiologist-identified structural abnormalities. We have updated the text in the Participants section of the Methods to clarify.

- Participants were to be excluded if they had any neuroradiologist-identified structural abnormalities of the brain; no participants in the current work were excluded for this reason.

We excluded two participants after their baseline nights because they extremely poor sleep that would have precluded experiment completion throughout the intervention period. Several datasets were excluded from analysis on either first or last nights because of technical issues or poor sleep. New Figure 1. and Supplementary Figure 1. detail participants included versus excluded in the study and in the analyses.

Although supplementary material includes detailed information about about inclusion and exclusion criteria, manuscript would benefit if you add shortly what you mean by “Medically healthy”. I’m not also sure, will the supplementary material be published as it is or is it openly available. If yes, authors should refer to The Strengthen study protocol, so that readers can find the details there.
Excluding the sleep disorders with overnight laboratory setting was good. However, it was unclear whether this is the same night as baseline night for the subjects. And were some subjects excluded based on the exclusion night?
By “Medically Healthy” we mean no acute illness at the time of participation and no diagnosed chronic illness or medications that could affect the outcome of the study. Additional information about exclusion criteria and the study protocol, beyond what is written in the Methods, is openly available on the ClinicalTrials.gov website. We have added clarifying information to the Participants section of the Methods.

- All participants were medically healthy, with no acute illness at the time of participation and no diagnosed chronic illness, including sleep disorders, or medications that may have influenced the outcome of the study.

We have added the following information about participant screening, with reference to where exclusion criteria can be found at the ClinicalTrials.gov website. This information also clarifies that there was a screening question about OSA and, separately, participants were evaluated for sleep quality during the pre-intervention baseline sleep visit. During the baseline sleep visit, two participants had loud snoring, breath holding and gasping accompanied by periods of wake. Given concern for OSA and overall poor sleep that would preclude experiment completion, these two participants were withdrawn after their baseline visit (see Figure 1.). We erred on the side of caution by removing these participants with indications of possible OSA. We have added the following information to the Participants section of the Methods to clarify screening procedures

- Participant recruitment began with an online survey. Potential participants were then further screened by phone call to see if they met eligibility criteria. They then underwent additional screening and an informed consent discussion in person prior to potential enrollment in the study. In general, screening consisted of questions regarding demographics, availability, medical and mental health, medications, and sleep. Participants with responses meeting exclusion criteria were not included in the study. As part of screening, for example, participants were asked about current or prior diagnoses of Obstructive Sleep Apnea (OSA); if yes, the participant was excluded. Full eligibility criteria can be found on the ClinicalTrials.gov website. As part of the pre-intervention baseline sleep visit, sleep technicians monitored for sleep quality. During the baseline

sleep visit, two participants had loud snoring, breath holding and gasping accompanied by periods of wake. Given concern for OSA and overall poor sleep that would preclude experiment completion, these two participants were withdrawn after their baseline visit (**Figure 1C**).

Figure about subject recruitment and exclusion would be helpful (could be the same chart where you include the excluded subjects based on the technical artifacts of the data).
We have added new Figure 1. and Supplementary Figure 1. to help clarify subject recruitment and exclusion.

Sleep quality surveys:
You could provide the information, why did you choose this REST-Q questionnaire.
We have added brief context as to the choice of the REST-Q to the Sleep Quality Surveys section of the Methods.

- After each overnight in the sleep lab, participants completed the 9-question Restorative Sleep Questionnaire (REST-Q) survey, which was designed by a panel of expert sleep specialists to define and measure restorative sleep [44,45] and chosen here for its potential to link the perceived restorative effects of sleep with the SWA that may underlie these restorative effects.

hd-EEG page 18:
Please, provide detailed information about your EEG (version), settings (e.g. was 0.1 Hz high-pass filtering on in recordings, electrode impedance limit). Also, you should mention somewhere how many hours of EEG-data was totally acquired (e.g. average+-std / night over subjects).
EEG version of the hardware (256-channel net) was MagStim Electrical Geodesics, Inc. (EGI), amplifier type NA400, and software was associated EGI Net Station Acquisition (v5.4.2). Further detail can be found in the hd-EEG Data Acquisition section of the Methods.
We have added the following details to the hd-EEG Data Acquisition section of the Methods.

- The hd-EEG system from MagStim Electrical Geodesics, Inc. (EGI) consisted of a hd-EEG net, an amplifier (NA 400), and a physiological input box.

The amplifier does not impose a hardware high-pass filter and so records from DC (near 0 Hz) to the sampling rate / 2. So, the data range recorded by the amplifier started as DC to 250 Hz. There was a separate, hardware low-pass filter placed on the initial recording with a cutoff of 100 Hz; therefore, the data range recorded including the hardware filter was DC to 100 Hz.
We then used software filters for online visualization during the experiment, for offline staging, and for offline analysis. All analyses were performed on hd-EEG data with the frequency range 0.5-40 Hz.
We have added a sentence about online filters for visualization; other filter cutoffs and ranges can be found in the hd-EEG Data Acquisition and hd-EEG Spectral Power Analyses sections of the methods.

- Then, the participant went to sleep, and a sleep technician performed stimulation protocols based on sleep features visible on the hd-EEG and PSG recordings (data visualization parameters in accordance with staging parameters found in the Sleep Staging section below).

We have added the recording electrode impedance limit as follows.

- Nets were then connected to EGI amplifiers that interface with EGI's Net Station Acquisition software (v5.4.2) for real-time data visualization. All recording electrode impedances were kept below 50,000 Ohm, with the reference electrode below 5,000 Ohm, as measured at the start of the recording.

We have added the mean and standard deviation for hour of EEG data totally acquired to the hd-EEG Data Acquisition section of the Methods.

- The total number of hours of EEG data acquired across the baseline, first, and last nights utilized in these analyses was 7.99 +/- 0.75 hours per night per participant.

MRI Data Acquisition page 19:
Please, provide information what head coil was used.
We have added the information about what head coil was used to the MRI Data Acquisition section of the Methods.

- MRI data were collected using a 3 Tesla MAGNUS (Microstructure Anatomy Gradient for Neuroimaging with Ultrafast Scanning, GE Healthcare) head-only MRI scanner. The head coil used was a 32-channel head radio frequency (RF) array (Nova Medical, Wakefield, MA). Structural images were acquired using T1- and T2-weighted images, with 0.8 mm isotropic voxels, and the following parameters.

Sleep staging page 19:
Why did you filter the data starting from 1 Hz and not 0.5 Hz as it is in American Academy of Sleep Medicine Guidelines for stage III delta waves? Please, shortly open in the text the criteria that used (e.g frequency ranges for different stages).
Thank you for this comment. We performed sleep staging in accordance with AASM guidelines as follows. Given the criteria for sleep staging is quite detailed and complex, here we will briefly describe frequencies for the most salient EEG events for the different stages, recognizing that the guidelines that were followed by our technicians were much more extensive.

Wake: alpha 8-13 Hz
N1: low-amplitude, mixed-frequency 4-7 Hz
N2: spindles 11-16 Hz and K-complexes (frequency not listed, but ≥0.5 seconds)
N3: slow waves 0.5-2 Hz
REM: sawtooth waves 2-6 Hz

Our hd-EEG data were bandpass filtered from 1–30 Hz. We recognize that this is a deviation from AASM guidelines, which recommend a lower high-pass cutoff for clinical scoring. On several nights' recordings, low-frequency signals (e.g., movement) interfered with the ability to stage data, and so a lower bound cutoff of 1 Hz was used to better visualize the data while preserving the canonical staging features listed above and allowing for reliable manual scoring. We would like to reassure the Reviewer that this does not impact the main results related to slow wave activity because, after sleep staging, all hd-EEG data were filtered 0.5-40 Hz and then artifact removal occurred. Given the order of these steps, low frequency slow wave activity, including between 0.5-1 Hz, without artifact, was what remained to be included in our spectral power analyses.

Hd-EEG preprocessing page 20:
How did you include "only" stage 2 and stage 3 epochs. Did you cut the data or do some other processing? Sometimes one stage can last only very short time (<30 s), so did you have some criteria that you only chose longer periods of the same sleep stage for analysis or what?
We agree that clarifying which epochs were included is important. Our sleep staging was performed in 30-second epochs, as in AASM guidelines, and so 30-second epochs with stage 2 or 3 were included. Data during time periods corresponding to other stages or data that contained artifact (i.e., a bad segment) was replaced with NaN values to maintain the temporal structure of the data; however, these NaN values were not incorporated into the final spectral power calculation.
We have added the following details about epoch inclusion to the Methods.
To hd-EEG Preprocessing:

- Only 30-second NREM epochs staged 2 or 3 were included in spectral power analyses.

To hd-EEG Artifact Rejection:

- All epochs exceeding the threshold were then removed. Removed epochs (due to artifact or a sleep stage that was not NREM 2 or 3) were replaced with NaN values to maintain the temporal structure of the data but were not included in the spectral power calculations.

Reviewer #6 (Remarks to the Author):

In this paper, TES-TI is applied for the first time with the aim of enhancing SW This approach offers several advantages over previous work using TES: stimulation can be delivered without being consciously perceived, it allows for more precise targeting of specific brain regions, and it enables concurrent EEG analysis during stimulation, which was not feasible with TES. The authors report that TES-TI increases SWA both during the 3-minute stimulation period and in the subsequent 3-minute post-stimulation window compared to the pre-stimulation baseline. While these findings are interesting and the methodological innovation is noteworthy, I am not fully convinced by some of the reported effects, particularly the claim that these findings translate directly to sleep-restorative processes. My major concerns are outlined belo

1) Concerns regarding the control group (15 kHz stimulation group):
If I understand correctly, certain effects are not reported for this group:
• The increase in SWA during and after stimulation relative to the pre-stimulation window is described for the 15 kHz–1 Hz group, but it remains unclear whether this effect was also present (or absent) in the 15 kHz group.
We agree that it could be helpful to show the data as in Figure 3. (original Figure 2.) for TES-only. We have added Supplementary Figure 4. with this information. Changes in SWA during stimulation (STIM-PRE) were not significant for TES-only. Changes in SWA after stimulation (POST-PRE) showed two peripheral clusters of significant electrodes; however, the effect was not global as in TES-TI and did not extend to the all-channel average after correction for multiple comparisons. In the context the small sample size for the TES group, we have adjusted our language to emphasize the preliminary nature of these analyses.

• similarly, the reported correlation between the change in SWA increase (T1 to T2) and changes in sleep quality ratings (T1 to T2) is only mentioned for the 15 kHz–1 Hz group. Was this relationship tested for the 15 kHz group?
Originally, we did assess for any correlation between the change in SWA increase and changes in overall REST-Q scores for the TES^{15KHz} group, and we did not find a significant correlation (N=5, R=0.70, p=0.233).
On the advice of another Reviewer, we adopted a new predictive modeling approach to the analyses comparing spectral power changes in STIM-PRE and POST-PRE periods to changes in overall REST-Q scores. This approach uses LASSO (Least Absolute Shrinkage and Selection Operator) estimation with 5-fold cross-validation to select model hyperparameters. With a sample size of 5 participants in the TES^{15KHz} group, this would mean only one observation per fold, which is not sufficient for cross-validation. For this reason, we have decided not to include analyses using the predictive modeling approach for the TES^{15KHz} group. We plan to perform this analysis once we have a greater number of participants in the TES^{15KHz} group.

In addition, the 15 kHz group has a very limited sample size. Although the authors note that a power analysis suggested that 5–9 participants would suffice, I am not convinced that such a

small group can reliably demonstrate the presence or absence of effects. And if such a small sample size is expected to yield meaningful effects, it would be important to clarify whether the effects reported for the full 15 kHz–1 Hz stimulation group are also observed when the original subgroups (2/meditation, 1/meditation, 2/sham meditation) are analyzed separately. Since these subgroups appear to have sample sizes comparable to the 15 kHz group, such an analysis would considerably strengthen the interpretation.
2) Potential confounding by original subgroup assignment:
In line with the previous point, I understand that for the baseline night and the first stimulation night (assuming neither meditation nor sham meditation had yet been administered) subgroup assignment is not relevant. However, since the majority of participants in the 15 kHz–1 Hz group appear to have undergone meditation training, I am concerned that effects observed on the last night may be substantially confounded. At a minimum, the statistical models should control for (a) whether participants received meditation training or not, and (b) whether they received 15 kHz–1 Hz stimulation once or twice per week

In consultation with Dr. Marshall (a statistician now added to the author list), and based on the Reviewer's comment, we fully acknowledge the issue in reporting statistical power analyses using one-sided tests when there was no preregistered directional hypothesis. We apologize for the improper usage of this analysis.

As the Reviewer suggested, we calculated power based on the observed data using the full TES-TI sample with a two-sided alpha. We removed the prior analysis. For the main findings pertaining to the TES-TI group, our analyses were highly powered (e.g., Figure 3, STIM-PRE, N=21, Cohen's d = 1.45, β = 0.99, α = 0.05, two-sided paired samples t-test). For the comparison between TES-TI and TES groups, our analysis was moderately powered (Figure 4A, B, TES^{15kHz}-TI^{1Hz} N=21, TES^{15kHz} N=7, Cohen's d=1.08, β = 0.66, α = 0.05, two-sided independent samples t-test). Although statistical power for this comparison was moderate (β = 0.66), a significant group difference was nevertheless detected with a large effect size (Cohen's d = 1.08). Reduced power primarily increases the likelihood of a Type II error (failure to detect a true effect) rather than a Type I error (false positive). The presence of a significant, large effect is not negated by the moderately powered test. Given that potential effects of high-frequency-only TES are an area of active investigation, but still rarely reported in comparison to TES-TI, we viewed this comparison as a valuable addition to our work. For these reasons, we decided to keep these results as part of the main findings. Analyses pertaining to only the TES^{15kHz} group were statistically underpowered and mostly showed no differences; therefore, Type II errors could not be reliable discounted. For these reasons, the results for these analyses have been moved to the Supplementary Information, they have been clearly qualified as preliminary (i.e., should be interpreted as exploratory and hypothesis-generating rather than confirmatory), and any previous conclusions drawn from these data have been removed from main text. In general, we have made changes to the text centered on more transparency about statistical power and more cautious phrasing about our conclusions. Below are a few examples of these numerous changes.

Figure 4C. (original Figure 3C-E.) now reports the early v. late comparison during stimulation for only the TES-TI group; the TES group results have been moved to Supplementary Figure 4. The Results section has been modified accordingly.

Added to Methods Statistics and Reproducibility

- Statistical power calculations were performed using the *pwr* functions in R (v4.5.2) for t-tests with a two-sided alpha at 0.05. The primary findings that used the observed data from participants that received the TES^{15kHz}-TI^{1Hz} intervention were highly powered (e.g., Figure 3, STIM-PRE, N=21, Cohen's d = 1.45, β = 0.99, α = 0.05, two-sided paired samples t-test). The comparison performed between TES^{15kHz}-TI^{1Hz} participants and TES^{15kHz} participants was moderately powered, but a significant difference was nevertheless detected with a large effect size (Figure 4A, B, TES^{15kHz}-TI^{1Hz} N=21, TES^{15kHz}

N=7, Cohen's d=1.08, β = 0.66, α = 0.05, two-sided independent samples t-test). Preliminary findings pertaining to only the TES^{15kHz} group, given the small sample size, were included in the Supplementary Information; the results of TES^{15kHz} group analyses should be considered exploratory and hypothesis-generating rather than confirmatory. Summary statistics for the main figures, including sample sizes and effect sizes (Cohen's d), can be found in Supplementary Table 4.

Added to Results, in several places

- Analyses were also repeated for the TES^{15kHz} group, but given the small samples size, results should be considered exploratory and hypothesis-generating (**Supplementary Table 3**).

To address the appropriateness of pooling participants from different subgroups, we used a linear mixed effects (LME) model to assess for any differences across subgroups. The LME was fit using with numerical maximum likelihood estimation using the 'lmer()' function of the 'lme4' package in R. In general, the model related the response variable (*y*) to experiment and the covariate of interest (*x*) as fixed effects with an interaction term, and participant as a random effect (that is, 'M1 = *y* ~ *x* * experiment + (1 | participant)'). We then tested the hypothesis that there was no interaction between *x* and the experiment by fitting a model with the interaction term removed (that is, 'M2 = *y* ~ *x* + experiment + (1 | participant)') and performing an asymptotic likelihood ratio test to compare the full and reduced models. In all cases, residual plots were examined for evidence against the assumptions of constant variance and normality. If no significant interaction was found, we tested for a main effect of the primary covariate by fitting a model with that term removed (that is, 'M3 = *y* ~ experiment + (1 | participant)') and performed an asymptotic likelihood ratio test to compare this model with the one including the covariate ('M2'). Specifically, for our analyses, SWA was the response variable, and TES-TI subgroup (Group 2, 3, or 4) and time period (PRE, STIM, POST) were fixed effects. Two separate models were applied, one for the first night and one for the last night. SWA was the response variable, TES^{15kHz}-TI^{1Hz} subgroup and time period (PRE, STIM, POST) were fixed effects. We found no significant interaction between subgroup and time period (First: f^2=0.16, p=0.079; Last: f^2=0.07, p=0.571) and found no main effect of subgroup (First: f^2 =0.23, p=0.482; Last: f^2=0.11, p=0.709). These results indicated that changes in SWA during and after stimulation were similar across subgroups and supported the decision to pool their data. Further supporting this approach was that a main effect of time period was found for both first and last nights (First: f^2=0.90, p=1.42x10^{-5}; Last: f^2=0.84, p=2.66x10^{-6}), in line with the analyses using aggregated subgroups. Thus, the qualitative conclusions were the same whether the subgroups were factored in with the LME or whether the subgroups were aggregated.
We have added these details to the Methods and Results.

3) Specificity of the correlation with subjective sleep ratings:
You report that the correlation with subjective sleep quality is specific to SWA, as no effect was observed for sigma power.
• How do the observed increases in theta power and decreases in beta power fit into this picture?
In line with other Reviewer comments, we adopted a predictive modeling approach to the analyses relating SWA to changes in sleep quality ratings. We performed a regression analysis using LASSO (Least Absolute Shrinkage and Selection Operator) with 5-fold cross-validation to identify which parameter inclusion improves the predicted outcome of REST-Q scores. Specifically, we used change in power in SWA, theta, beta, low sigma, and high sigma bands during STIM-PRE and POST-PRE windows as predictors (10 independent variables) for sleep quality (dependent variable). We ran this model for first and last nights as well as the difference between last and first nights. We found that only SWA during the STIM-PRE window improves

predictive performance for the difference between last and first nights in the TES-TI group. In line with our previous finding, increases in SWA during STIM-PRE on the last night relative to the first night were positively associated with increases in sleep quality ratings. Thus, the inclusion of theta and beta power did not improve predictive performance.

• I also find the rationale behind the change-score correlations somewhat unclear. What is the underlying hypothesis about what happens between the first and the last night (given that the night-intervention is the same) that would explain a change in how participants rate their sleep quality? Wouldn't it also be expected that simple change scores of other sleep parameters - such as overall SWA, SWA in the first half of the night etc. - would correlate with changes in subjective sleep quality ratings? Clarifying whether such analyses were performed would help in evaluating the specificity of the reported effect.
Thank you for raising this point. Part of our hypothesis includes the idea that the effects of TES-TI, especially during sleep, may be mediated by changes in neuroplasticity. The study was designed with this in mind, as TES-TI is delivered in repeated blocks across repeated nights, in which potential plastic changes can be reinforced. We hypothesized that some participants would demonstrate greater stimulation-associated changes in SWA on their last intervention night as compared to their first, and that these changes could be associated with improved sleep quality and, more broadly, well-being.
We agree that investigating the relationship between other sleep parameters and REST-Q ratings is an important topic. Given the initial relationship identified in this work, we are planning to perform such analyses in future work to better understand the relationship between objective and subjective sleep quality measures in our data. However, we felt these additional comparisons were not within the scope of the current work, which focused on TES-TI and SWA changes.

4) Wording in the abstract:
The final sentence of the abstract should be phrased more cautiously. The reported effects on SWA did not extend beyond the stimulation window and the immediate post-stimulation period; therefore, it is not justified to conclude that sleep itself was enhanced. Similarly, it cannot be claimed that 15 kHz–1 Hz stimulation increases restorative processes, as the evidence is primarily based on correlations.
Thank you for raising this concern. We agree that tempering our language is important given the findings reported. We have changed the final sentence of the abstract and edited the rest of the manuscript to include more cautious phrasing.

- Conclusions: To our knowledge, this is the first study demonstrating that TES-TI enhances SWA and potentially its restorative functions.

Minor:
- In the Methods section you state that all reported p-values are unadjusted unless otherwise specified. Could you please elaborate on the rationale for this choice?
Originally, we chose to report all unadjusted p-values given that the adjusted values can vary depending on the correction procedure used. In instances where we reported both unadjusted and adjusted values, the correction procedure made a difference in the interpretation.
In the updated manuscript, we have clarified how we are performing multiple comparison testing and now report all results using adjusted p-values. Both unadjusted and adjusted p-values for all analyses can be found in Supplementary Table 4.
As such, the Statistics and Reproducibility section of the Methods has been updated:

- To assess for statistical significance in the context of multiple comparisons, the Benjamini-Yekutieli procedure was used to correct families of comparisons to a false discovery rate of 5% (*fdr_bh* in MATLAB). Comparisons were grouped into families based on conceptual

similarity and corrections were applied within each family. P-values reported in the text are adjusted according to this procedure; if adjustment resulted in a number greater than 1, p-values are reported as $p > 0.999$. Both original, unadjusted p-values and the reported adjusted p-values can be found in Supplementary Table 4. For analyses that do not report adjusted p-values (e.g., age, sex, number of stimulation protocols, etc.), tests were conceptually isolated and not considered to be part of a family requiring correction for multiple comparisons.

Reviewers' comments:

Reviewer #2 (Remarks to the Author):

The authors have satisfactorily addressed all of my comments. As to my comment #2, they may optimize the revision concerning the LASSO regression, by reporting R^2 or MSE on the held-out test folds (not the full dataset) to assess whether SWA predicts sleep quality in new data, which is the purpose of the CV approach. Running the model 3 times (PRE,STIM,STIM-PRE) does reintroduce a multiplicity problem, just in a different form. Also, if I understand correctly, the authors are now topographically mapping the R2 values which is another implicit form of multiple comparisons (across electrodes). Or are the R^2 values in the topographic maps derived from the cross-validated model? Please clarify.
Thank you for raising these concerns; we agree with the Reviewer that these aspects of the LASSO regression can be reported more clearly.
We have added the MSE on the held-out test folds when reporting our results, as follows:

- Added to the **Methods**:
    - Mean squared error (MSE) for the held-out test folds is also reported.
- Added to the **Results**:
    - For first and last night data (λ_min = 2.98, cross-validated Mean Squared Error (MSE) = 313.0), no factors improve predictive performance. For the difference between last and first nights (λ_min = 9.44, cross-validated MSE = 298.6), only one factor improves predictive performance: the change in SWA during the STIM-PRE period.

We agree with the Reviewer that multiplicity is a concern when numerous analyses are performed on the same dataset. In this case, we respectfully suggest that the typical multiplicity problem, which concerns controlling the family-wise error rate for results across repeated null hypothesis significance tests, does not apply directly. The key difference is that in traditional null hypothesis testing, one targets a specific significance level (or error rate), and the correction for multiple comparisons is used to obtain the desired error rate across multiple tests. In the predictive modeling framework, there is no analogous concept of error rate, and thus, traditional correction for multiple comparisons does not map cleanly onto the analysis; and, to our knowledge, there is no single, standardized approach to the multiplicity issue.
That said, it is true that the likelihood of a spurious result (analogous to a false positive in hypothesis testing) is increased when evaluating multiple models. We acknowledge that the results should be interpreted with appropriate caution, and so we have adjusted our language around the interpretation of these analyses.

- Added to the **Methods**:
    - The predictive modeling approach does not use the null hypothesis testing framework; instead, a training-testing split of the data via cross-validation plays the role of preventing overfitting and ensuring that the results for each model cannot be explained by random variation. Nevertheless, in the context of multiple models, findings should be considered preliminary rather than confirmatory.
- Added to the **Discussion**:
    - A promising, albeit preliminary, indication of this study was the improvement in the restorative quality of sleep when comparing the first and last intervention nights of TES^{15kHz}-TI^{1Hz}, as reported by subjects through REST-Q scores.

We agree that the topographic mapping of R^2 values could be more clearly explained. The R^2 values plotted topographically are not derived from the cross-validated model. The R^2 values plotted come from simple linear regressions and are meant as descriptive statistics, a way to visualize the spatial patterns in the data. We have made the following changes to more clearly

convey where the R^2 values come from and that they are intended to be descriptive rather than inferential.

- Added to the **Methods**:
  - To visualize potential predictive relationships, linear regression models were used, and the line of best fit ($y = \beta_1 * x + \beta_0$) and R^2 values are reported. To explore whether the predictive power in the observed data was spatially localized, for descriptive purposes, R^2 values from the linear regression models were plotted topographically.
- Added to the **Results**:
  - To further investigate whether the predictive power in the observed data was spatially localized, R^2 values are plotted topographically (**Figure 6A**, Right). We find relatively high R^2 values globally, with the greatest values in the left frontal region. However, we note that these topographies are purely descriptive.
