# Supplementary material for: Enhancement of sleep slow wave activity using transcranial electrical stimulation with temporal interference: an interim analysis of the STRENGTHEN study
Source: Commun Med (Lond). 2026 Jul 1;6:549. doi: 10.1038/s43856-026-01744-x (PMC13687101; doi:10.1038/s43856-026-01744-x)
Supplement: Supplementary file 2 [file 43856_2026_1744_MOESM2_ESM.pdf]

Supplementary Information (4 figures, 4 tables)

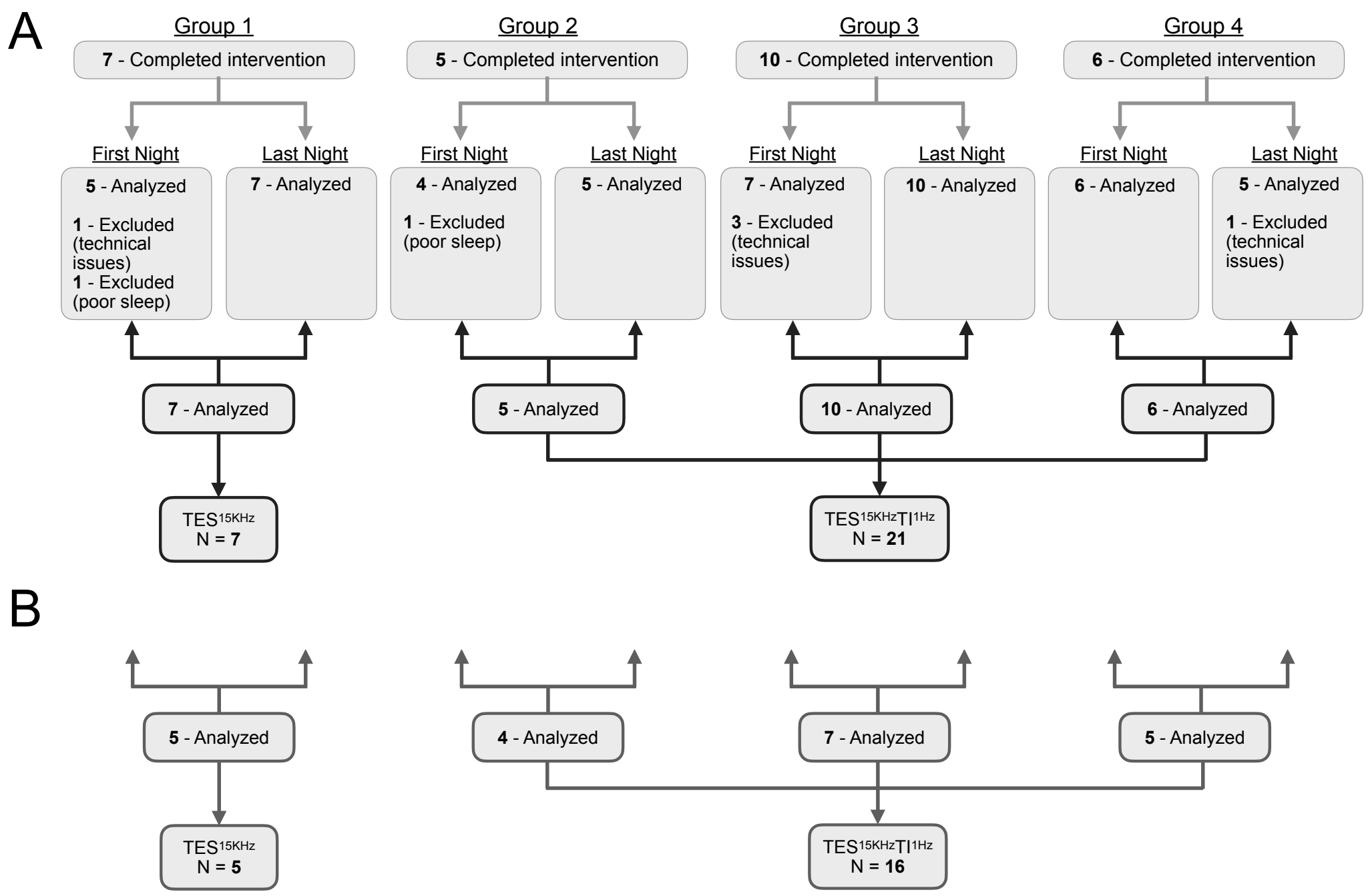

Supplementary Figure 1. Participant flow diagram for analyses. A. *Analyses using the average across first and last nights included 21 participants in the TES^{15kHz}-TI^{1Hz} group and 7 participants in the TES^{15kHz} group.* ***B.*** *Analyses for within-participant comparisons (i.e., comparing across nights) included 16 participants in the TES^{15kHz}-TI^{1Hz} group and 5 participants in the TES^{15kHz} group given that the data from some nights was not usable.*

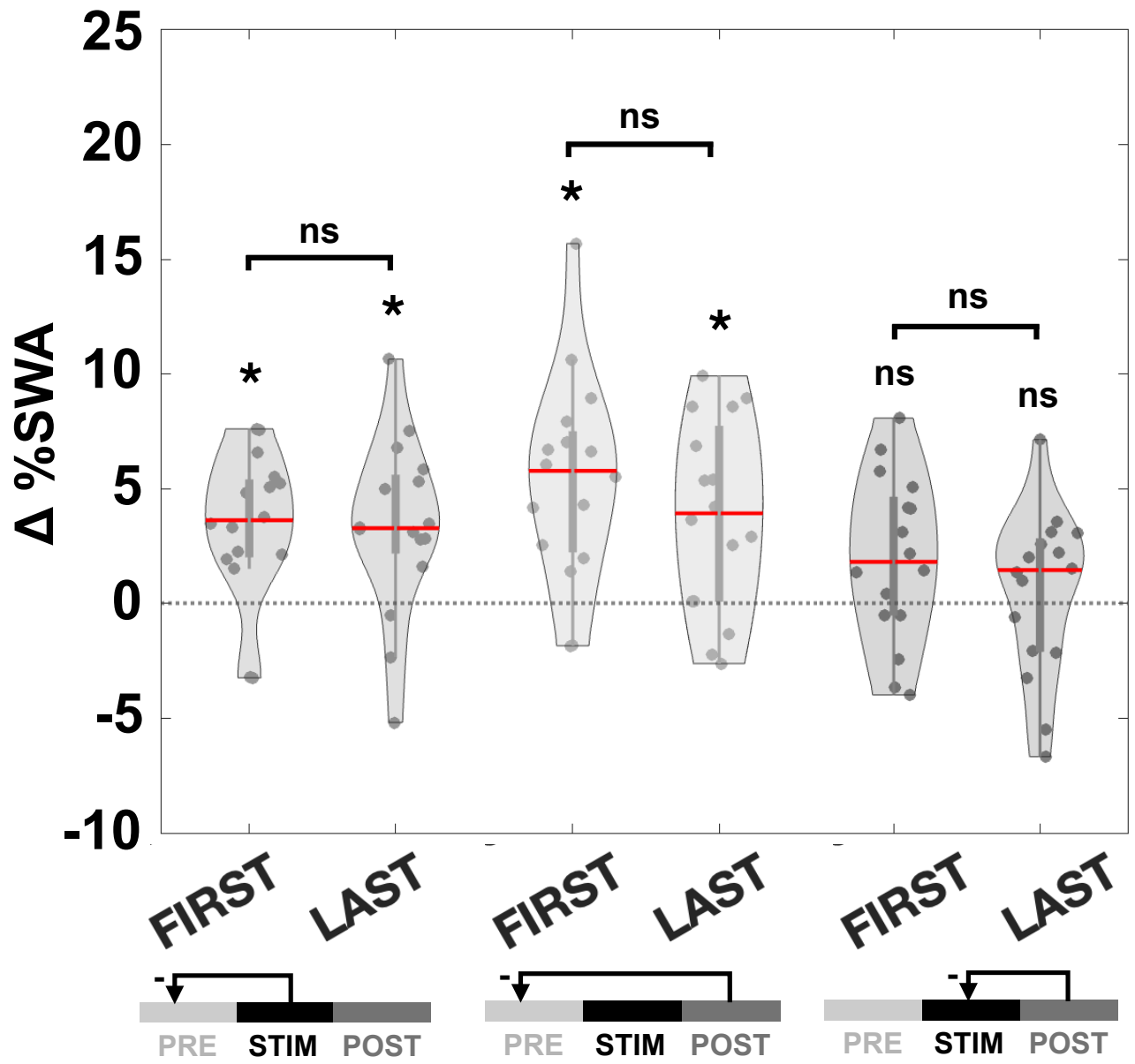

Supplementary Figure 2. Consistent TES^{15kHz}-TI^{1Hz} effects on first and last intervention nights*. For participants with usable data during TES^{15kHz}-TI^{1Hz} stimulation on both first and last nights (N = 16), across all channels, there is a main effect with a significant difference in the average normalized SWA (%SWA) across PRE, STIM, and POST periods (df = 2, F = 25.16, p = 3.84x10^{-7}), but there is no main effect across nights (df = 1, F = 0.31, p = 0.589) and there is no interaction effect between time period and night (df = 2, F = 0.83, p = 0.448) when evaluated with a two-way repeated measures ANOVA. (Left) %SWA during stimulation compared to before stimulation (STIM-PRE) shows similar increases on the first and last intervention night, with no significant difference between nights (STIM-PRE, N = 16; First: t = 4.24, unadjusted p = 7.08x10^{-4}, adjusted p = 5.20x10^{-3}, d = 1.06; Last: t = 3.50, unadjusted p = 3.23x10^{-3}, adjusted p = 0.012, d = 0.87; First v. Last: t = 0.04, unadjusted p = 0.966, adjusted p >0.999, d = 0.01). (Middle) Increases in %SWA after stimulation compared to before stimulation (POST-PRE) are also similar on the first and last intervention night, with no significant difference between nights (POST-PRE, N = 16; First: t = 4.78, unadjusted p = 2.42x10^{-4}, adjusted p = 3.60x10^{-3}, d = 1.20; Last: t = 3.68, unadjusted p = 2.25x10^{-3}, adjusted p = 0.011, d = 0.92; First v. Last: t = 1.02, unadjusted p = 0.318, adjusted p = 0.873, d=0.25). (Right) %SWA does not differ after stimulation compared to during stimulation either on first or last intervention nights, with no significant difference between nights (POST-STIM, N = 16; First: t = 2.17, unadjusted p = 0.046, adjusted p = 0.136, d = 0.54; Last: t = 0.52, unadjusted p = 0.612, adjusted p >0.999, d = 0.13; First v. Last: t = 1.18, unadjusted p = 0.249, adjusted p = 0.873, d=0.29). Two-sided paired samples t-tests used, Benjamini-Yekutieli procedure to correct for multiple comparisons.*

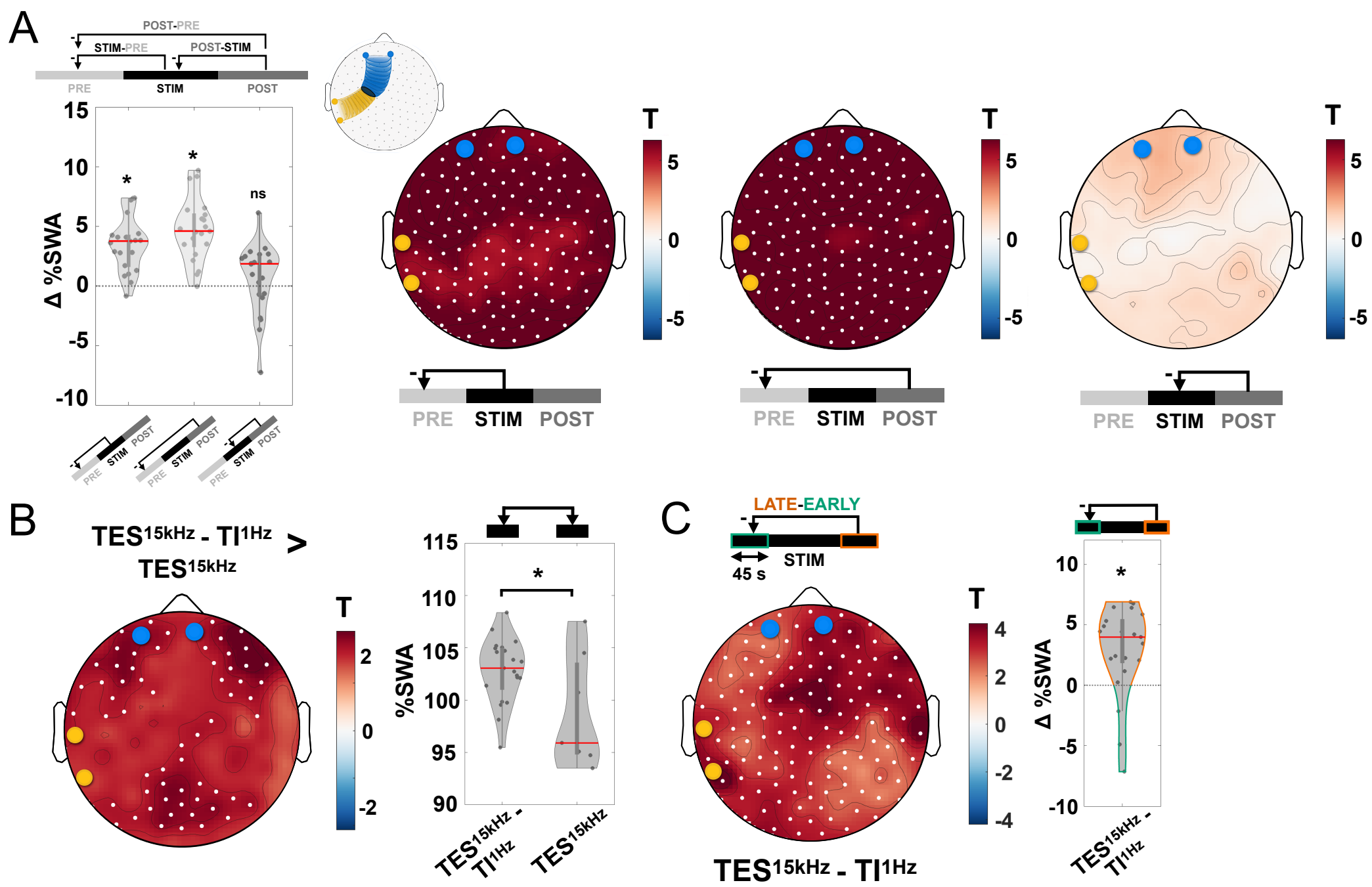

Supplementary Figure 3. Main results using narrower-band SWA. *Results using a narrower 0.5-2 Hz band are similar to findings in the broader 0.5-4 Hz band.* ***A.*** *(Left) Across all channels, normalized SWA (%SWA) increases during and after TES^{15kHz}-TI^{1Hz} compared to before (N=21; STIM-PRE, t = 6.95, unadjusted p = $9.63x10^{-7}$, adjusted p = $2.65x10^{-6}$, d = 1.52; POST-PRE, t = 8.30, unadjusted p = $6.57x10^{-8}$, adjusted p = $3.61x10^{-7}$, d = 1.81). %SWA does not change after stimulation compared to during stimulation (POST-STIM, t = 0.99, unadjusted p = 0.333, adjusted p = 0.610, d = 0.22). Two-sided paired samples t-tests, Benjamini-Yekutieli procedure to correct for multiple comparisons. Median shown as solid red line. (Right) Increases in %SWA during and after stimulation are global (STIM-PRE: 1 positive cluster, 185 significant electrodes, $\bar{p}$ = $7.55x10^{-6}$; POST-PRE: 1 positive cluster, 185 significant electrodes, $\bar{p}$ = $1.19x10^{-6}$). No channels have statistically significant changes in %SWA after stimulation compared to during stimulation (POST-STIM: 0 clusters, 0 significant electrodes, $\bar{p}$ = 0.379).* ***B****. (Left) During stimulation, %SWA is increased across multiple channels in TES^{15kHz}-TI^{1Hz} as compared to TES^{15kHz} (2 positive clusters, 80 significant electrodes, $\bar{p}$ = 0.033). (Right) Average %SWA across channels with statistically significant differences between TES^{15kHz}-TI^{1Hz} and TES^{15kHz} is shown. %SWA is higher during TES^{15kHz}-TI^{1Hz} as compared to TES^{15kHz} (TES^{15kHz}-TI^{1Hz} N = 21, TES^{15kHz} N = 7, t = 2.40, p = 0.024, d = 1.05, two-sided independent samples t-test). Median shown as solid red line.* ***C****. Within the three-minute period during stimulation, EARLY and LATE forty-five second intervals are considered; the difference in %SWA between the LATE and EARLY stimulation intervals is compared during TES^{15kHz}-TI^{1Hz}. (Left) %SWA is greater in the LATE interval during TES^{15kHz}-TI^{1Hz}. Increases in %SWA later in stimulation are global (1 positive cluster, 184 significant electrodes, $\bar{p}$ = $8.51x10^{-3}$). (Right) Averaged across all channels with statistically significant increases, %SWA is greater in the LATE interval (N = 21, t = 3.45, p =$2.56x10^{-3}$, d = 0.75, two-sided paired samples t-tests). Topography of T-statistic shown; channels with statistically significant changes are indicated by white dots. Approximate locations of stimulating electrodes are shown in blue (E_1) and yellow (E_2).*

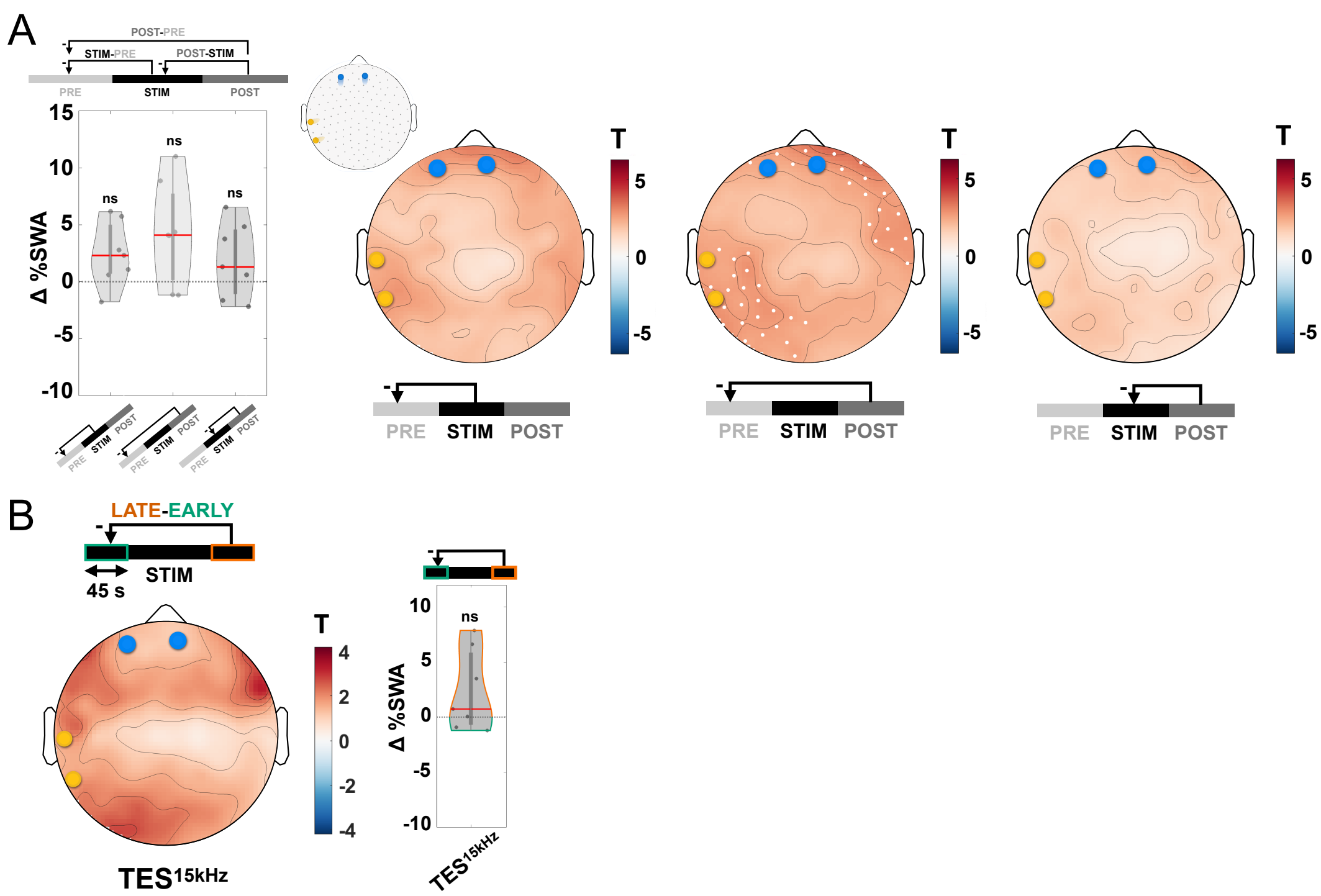

Supplementary Figure 4. Preliminary results for SWA during and after TES^{15kHz}. *As the TES^{15kHz} group sample size was small (N = 7), results here should be considered exploratory and hypothesis-generating rather than confirmatory.* ***A****. Across all channels, normalized SWA (%SWA) does not change during or after stimulation compared to before, after correction for multiple comparisons (N = 7; STIM-PRE, t = 2.26, unadjusted p = 0.064, adjusted p = 0.177, d=0.85; POST-PRE, t = 2.49, unadjusted p = 0.047, adjusted p = 0.177, d = 0.94). %SWA does not change after stimulation compared to during stimulation (POST-STIM, t = 1.52, unadjusted p = 0.180, adjusted p = 0.330, d = 0.57). Two-sided paired samples t-tests, Benjamini-Yekutieli procedure to correct for multiple comparisons. Median shown as solid red line.* ***B.*** *No channels have statistically significant changes in %SWA during stimulation compared to before stimulation or after stimulation compared to during stimulation (STIM-PRE: 0 clusters, 0 significant electrodes, $\bar{p}$ = 0.104; POST-STIM: 0 clusters, 0 significant electrodes, $\bar{p}$ = 0.218). Two separate, peripheral clusters of channels show statistically significant increases in %SWA after stimulation compared to before (POST-PRE: 2 positive clusters, 69 significant electrodes, $\bar{p}$ = 0.034). Topography of T-statistic shown; channels with statistically significant changes are indicated by white dots. Approximate locations of stimulating electrodes are shown in blue (E_1) and yellow (E_2).*

	Baseline Night	First Night	Last Night	Test Statistic (X^2 or F)	Unadjusted p-value, Adjusted p-value
Total Sleep-Wake Period (hr)	7.74 ± 1.16	7.62 ± 0.52	7.49 ± 0.23	(X^2) 0.40	0.819, p > 0.999
Total Sleep Time (hr)	6.49 ± 1.48	6.45 ± 1.40	6.76 ± 0.82	(X^2) 1.20	0.549, p > 0.999
Sleep Onset Latency (min)	29.50 ± 13.52	18.30 ± 13.67	11.90 ± 5.53	(X^2) 8.40	0.015, 0.747
REM Latency (hr)	2.53 ± 0.60	2.12 ± 0.74	2.16 ± 0.90	(X^2) 0.40	0.819, p > 0.999
Sleep Efficiency (%)	83.46 ± 12.35	84.38 ± 15.96	90.13 ± 8.61	(X^2) 3.60	0.165, p > 0.999
Number of Arousals	38 ± 17	36 ± 22	70 ± 41	(X^2) 4.80	0.091, 0.906
Arousal Index (arousals / hr)	6.06 ± 2.94	5.40 ± 2.68	10.73 ± 6.74	(X^2) 4.80	0.091, 0.906
Number of Awakenings	11 ± 6	14 ± 5	18 ± 7	(X^2) 2.80	0.247, p > 0.999
Wake After Sleep Onset (min)	45.10 ± 43.64	51.50 ± 58.02	31.30 ± 31.57	(X^2) 0.40	0.819, p > 0.999
Stage N1 (%)	2.45 ± 2.16	2.90 ± 1.80	3.97 ± 4.49	(X^2) 0.40	0.819, p > 0.999
Stage N2 (%)	61.38 ± 4.47	64.87 ± 7.52	66.85 ± 4.46	(X^2) 1.60	0.449, p > 0.999
Stage N3 (%)	16.72 ± 4.00	10.74 ± 4.11	9.81 ± 6.49	(X^2) 5.20	0.074, 0.906
Stage REM (%)	19.45 ± 4.19	21.49 ± 7.06	19.38 ± 2.81	(X^2) 1.20	0.549, p > 0.999
Whole-Night SWA (dB / Hz)	11.96 ± 2.77	11.41 ± 2.61	11.37 ± 2.78	(F) 3.58	0.077, 0.906
First 4hr SWA (dB / Hz)	12.15 ± 2.98	11.72 ± 2.84	11.60 ± 3.18	(F) 2.30	0.163, p > 0.999

Supplementary Table 1. Preliminary results for sleep characteristics across nights in TES^{15kHz}. *As the TES^{15kHz} group sample size was small, results here should be considered exploratory and hypothesis-generating rather than confirmatory. Across baseline, first, and last nights, TES^{15kHz} (N = 5) participants show no significant differences in any sleep characteristics after correction for multiple comparisons (Friedman test or one-way repeated measures ANOVA, Benjamini-Yekutieli procedure to correct for multiple comparisons). Mean ± standard deviation. REM: Rapid Eye Movement, SWA: Slow Wave Activity.*

	STIM-PRE		POST-PRE	
	Test Statistic (T)	Unadjusted p-value, Adjusted p-value	Test Statistic (T)	Unadjusted p-value, Adjusted p-value
Theta (4–8 Hz)	4.28	3.65×10^{-4}, 0.011	3.89	9.00×10^{-4}, 0.011
Alpha (8–12 Hz)	1.74	0.097, 0.450	1.34	0.195, 0.805
Sigma (Low, 9–12 Hz)	0.68	0.505, p > 0.999	0.80	0.433, p > 0.999
Sigma (High, 12–16 Hz)	-2.75	0.012, 0.092	-3.95	7.88×10^{-4}, 0.011
Beta (16–25 Hz)	-3.30	3.60×10^{-3}, 0.034	-1.97	0.063, 0.337
Gamma (25–40 Hz)	-2.06	0.053, 0.326	-1.23	0.232, 0.862

Supplementary Table 2. Statistics for TES^{15kHz}-TI^{1Hz} effects on other frequency bands. *For each canonical frequency band, average power spectral density (PSD) values were compared during (STIM-PRE) and after (POST-PRE) stimulation as compared to before stimulation for TES^{15kHz}-TI^{1Hz} participants (N = 21). Two-sided paired samples t-tests used, Benjamini-Yekutieli procedure to correct for multiple comparisons. T-statistics, unadjusted p-values, and adjusted p-values reported. As compared to before stimulation, during (STIM-PRE), in addition to SWA (Figure 3), theta power increases, and beta power decreases. There is also a decrease in high sigma power, but it is not significant after correction for multiple comparisons. Similarly, after stimulation (POST-PRE), in addition to SWA (Figure 3), theta power increases, and high sigma power decreases.*

	STIM-PRE		POST-PRE	
	Test Statistic (T)	Unadjusted p-value, Adjusted p-value	Test Statistic (T)	Unadjusted p-value, Adjusted p-value
Theta (4–8 Hz)	1.01	0.352, p > 0.999	1.95	0.099, p > 0.999
Alpha (8–12 Hz)	-0.75	0.483, p > 0.999	-0.74	0.486, p > 0.999
Sigma (Low, 9–12 Hz)	-0.98	0.363, p > 0.999	-0.94	0.386, p > 0.999
Sigma (High, 12–16 Hz)	-1.43	0.202, p > 0.999	-2.74	0.034, p > 0.999
Beta (16–25 Hz)	-0.46	0.662, p > 0.999	-0.84	0.431, p > 0.999
Gamma (25–40 Hz)	-0.96	0.375, p > 0.999	-1.59	0.164, p > 0.999

Supplementary Table 3. Preliminary statistics for other frequency bands during and after TES^{15kHz}. *As the TES^{15kHz} group sample size was small, results here should be considered exploratory and hypothesis-generating rather than confirmatory. For each canonical frequency band, average power spectral density (PSD) values were compared during (STIM-PRE) and after (POST-PRE) stimulation as compared to before stimulation for TES^{15kHz} participants ($N = 7$). No significant changes in power for any frequency band are found during or after stimulation, after correction for multiple comparisons. Two-sided paired samples t-tests used, Benjamini-Yekutieli procedure to correct for multiple comparisons. T-statistics, unadjusted p-values, and adjusted p-values reported.*

Analysis	Figure	Number of Participants	Median, [Lower CI, Upper CI]	Statistical Test	Degrees of Freedom	Test Statistic	Unadjusted p-Value	Adjusted p-Value	Effect Size (Cohen's d)
PRE, STIM, POST (%SWA)	Fig 3A	TES^{15KHz}-TI^{1Hz} = 21		One-Way Repeated Measures ANOVA	2	F = 30.95	7.54×10^{-9}		
STIM-PRE (Δ%SWA)	Fig 3A	TES^{15KHz}-TI^{1Hz} = 21	3.48, [2.52, 4.83]	Paired T-Test	20	T = 6.64	1.81×10^{-6}	4.97×10^{-6}	1.45
POST-PRE (Δ%SWA)	Fig 3A	TES^{15KHz}-TI^{1Hz} = 21	4.61, [3.47, 6.16]	Paired T-Test	20	T = 7.46	3.38×10^{-7}	1.86×10^{-6}	1.63
POST-STIM (Δ%SWA)	Fig 3A	TES^{15KHz}-TI^{1Hz} = 21	1.42, [-1.03, 1.92]	Paired T-Test	20	T = 0.63	0.536	0.982	0.14
STIM (%SWA)	Fig 4B	TES^{15KHz}-TI^{1Hz} = 21<br>TES^{15KHz} = 7	TES^{15KHz}-TI^{1Hz} = 104.73, [103.14, 106.35]<br>TES^{15KHz} = 98.89, [95.73, 105.24]	Independent T-Test	26	T = 2.47	0.020		1.08
STIM LATE-EARLY (Δ%SWA)	Fig 4C	TES^{15KHz}-TI^{1Hz} = 21	3.73, [1.15, 4.61]	Paired T-Test	20	T = 3.47	2.41×10^{-3}		0.76
STIM-PRE (Theta ΔPSD)	Fig 5A	TES^{15KHz}-TI^{1Hz} = 21	0.15, [0.10, 0.29]	Paired T-Test	20	T = 4.28	3.65×10^{-4}	0.011	0.93
STIM-PRE (Beta ΔPSD)	Fig 5A	TES^{15KHz}-TI^{1Hz} = 21	-4.40×10^{-3}, [-9.90×10^{-3}, -2.20×10^{-3}]	Paired T-Test	20	T = -3.30	3.60×10^{-3}	0.034	-0.72
POST-PRE (Theta ΔPSD)	Fig 5C	TES^{15KHz}-TI^{1Hz} = 21	0.17, [0.11 0.37]	Paired T-Test	20	T = 3.89	9.00×10^{-4}	0.011	0.85
POST-PRE (Sigma ΔPSD)	Fig 5C	TES^{15KHz}-TI^{1Hz} = 21	-3.12×10^{-2}, [-6.94×10^{-2}, -2.14×10^{-2}]	Paired T-Test	20	T = -3.95	7.88×10^{-4}	0.011	-0.86

Supplementary Table 4. Summary of Figure Statistics. *95% Confidence Intervals (CI) shown. All tests were two-sided. The Benjamini-Yekutieli procedure was used to correct families of comparisons to a false discovery rate of 5%.*
