# Supplementary material for: Enhancement of sleep slow wave activity using transcranial electrical stimulation with temporal interference: an interim analysis of the STRENGTHEN study
Source: Commun Med (Lond). 2026 Jul 1;6:549. doi: 10.1038/s43856-026-01744-x (PMC13687101; doi:10.1038/s43856-026-01744-x)
Supplement: Supplementary file 3 [file 43856_2026_1744_MOESM3_ESM.docx]

**Description of Additional Supplementary Files**

File name: Supplementary Data 1-5

Description: Source data
